# One-Step Photoreduction of Nitroarenes to Nitrosoarenes

**DOI:** 10.1021/acs.orglett.6c02016

**Published:** 2026-06-25

**Authors:** Justice A. Keech, Narva Deshwar Kushwaha, James R. Bour

**Affiliations:** Department of Chemistry, 2954 Wayne State University, 5101 Cass Avenue, Detroit, Michigan 48202, United States

## Abstract

Nitrosoarenes are versatile synthons, but their selective synthesis from abundant feedstocks remains challenging because conventional methods often require harsh conditions that can result in over-reduction and over-oxidation. Here, we report a photomediated deoxygenation of nitroarenes to nitrosoarenes using electron-poor phosphines as oxygen atom acceptors. Careful selection of the oxygen atom acceptor enables selective arrest at the nitroso oxidation state, affording nitrosoarenes directly from nitroarenes in good yields under mild conditions.

## Graphical abstract

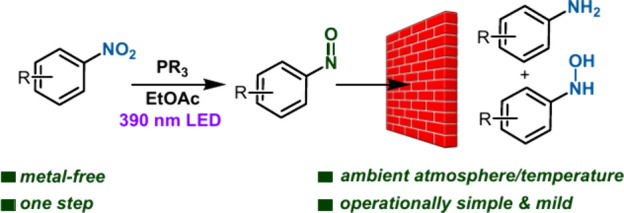

Nitrosoarenes are useful building blocks that participate in a wide range of transformations,^1^ including nitroso–ene,^2–4^ hetero-Diels–Alder reactions,^5–8^ radical additions,^9–12^ coordination to transition metals,^13–15^ and reversible dimerization to form azodioxy motifs.^15–18^ Their diverse reactivity stems from their unique electronic structure, which allows them to function as electrophiles, pronucleophiles, and even efficient radical acceptors.^3,19^ The products of these reactions have been utilized in the synthesis of important materials and molecules from complex heterocycles to crystalline framework materials.^8,16,20–26^ As such, simple, general routes to aryl nitroso compounds from abundant feedstocks remain broadly important.

Despite their synthetic utility, the practical synthesis of nitrosoarenes is still nontrivial.^1,19,27^ They are generally formed through three routes: direct nitrosylation using NO^+^ equivalents (Scheme 1a),^28–31^ oxidation of anilines or hydroxylamines (Scheme 1b),^32–35^ and reduction of nitroarenes to the corresponding nitrosoarenes (Scheme 1c).^36^ Each of these routes is complicated by the narrow stability window of the nitrosoarene products, which are susceptible to condensation reactions and/or further reduction or oxidation under the conditions that form them. For instance, oxidation of anilines often forms other products, including nitro, azo, or azoxy products.^32,33^ Oxidations of *N*-aryl hydroxylamines are generally more efficient,^37,38^ but these compounds are generally less available than nitroarenes. Ultimately, that which makes nitrosoarenes useful synthons, their wide-ranging reactivity, also makes them difficult to synthesize.

**Scheme 1:**
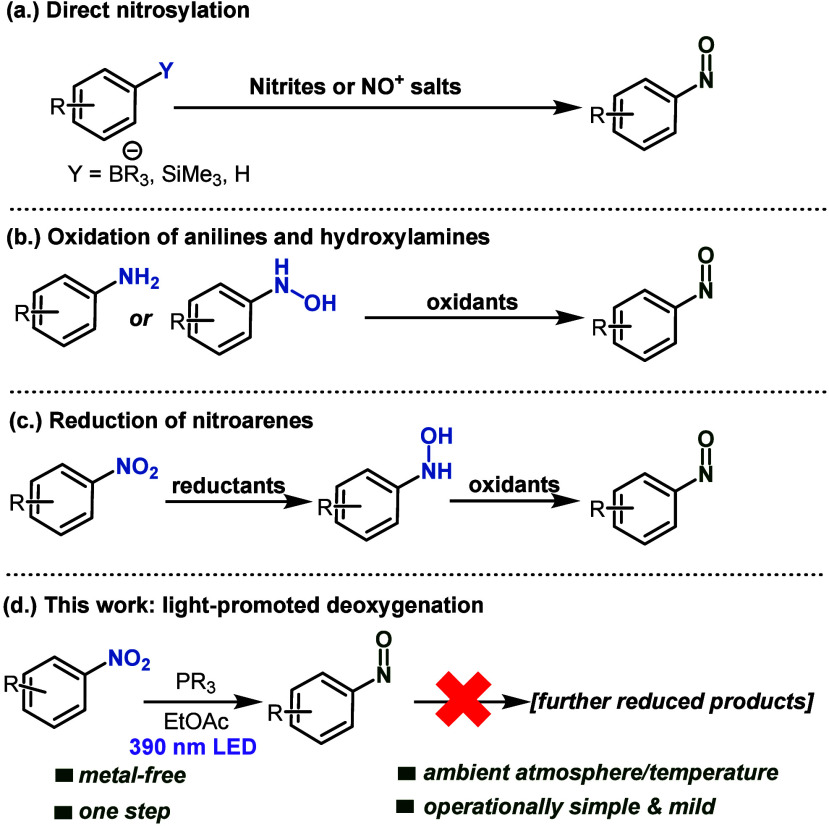
Synthetic Routes toward Nitrosoarenes

Among potential nitrosoarene precursors, nitroarenes are especially attractive because they are broadly available and differ from nitrosoarenes by only a single oxygen atom. However, as with other precursor classes, selectively engaging nitroarene without further reaction of the nitrosoarene product remains challenging.^1,16,17,19,39^ One strategy to address this problem is to exploit the distinct reactivity of photoexcited nitroarenes. Recent advances in nitroarene photochemistry have shown that n to π* excitation of nitroarenes generates a potent oxidant that readily reacts with a wide range of mild reducing partners, including alcohols,^40–42^ thiols,^43,44^ alkenes,^45–50^ borane derivatives,^51,52^ hydrazines,^53^ and phosphines.^38,50,54–56^ In principle, the potent oxidizing power of photoexcited nitroarenes could be paired with reductants that are sufficiently mild so as to avoid activating the nitrosoarene product. In practice, however, arresting the reaction at the nitrosoarene oxidation state remains challenging outside of substrates with intramolecular H atom donors or oxygen atom acceptors. Mechanistic studies of light-promoted nitroarene reductions often provide strong evidence for nitrosoarene intermediates, but general methods that exploit this activation mode to isolate nitrosoarenes have not yet been reported.^38^ Collectively, these examples establish photochemical activation of nitroarenes as a promising platform for nitrosoarene formation while highlighting that general, isolable methods must combine excited-state selectivity with suppression of downstream nitrosoarene reactivity.

Here, we report a single-step conversion of nitroarenes to nitrosoarenes that selectively arrests at the nitroso oxidation state (Scheme 1d). Realization of this transformation was facilitated by the identification of an oxygen atom acceptor that does not react with nitrosoarene products. We show that electron-poor phosphines do not degrade model nitrosoarenes at room temperature but still serve as competent oxygen atom acceptors with photoexcited nitroarenes, enabling isolation of nitrosoarene or, more typically for the solid state, as the corresponding azodioxy dimer. Because they are typically in rapid equilibrium in solution phases, we use the terms interchangeably here.^57^ This method proceeds under ambient conditions, does not require an intramolecular hydrogen atom donor or oxygen atom acceptor, and tolerates unprotected aldehydes, ketones, amides, esters, aliphatic alcohols, and select heteroarenes.

On the basis of previous examples of photomediated nitroarene reductions, we anticipated that the solvent and reductant would need to be carefully chosen to limit the availability of hydrogen atoms and protons. Phosphines in polar aprotic solvents meet these criteria. Radosevich and co-workers recently showed that phosphines serve as clean intermolecular oxygen atom acceptors with photoexcited nitroarenes (Scheme 2a). However, they also showed that phosphines reduce nitrosoarenes through mechanistically distinct thermal pathways, i.e., dark reactions, that are speculated to occur through phosphine-mediated activation of nitrosoarenes (Scheme 2b).^38,56^ Though the exact mechanisms of these dark reactions are unknown, mitigation of these reactions is likely to be important for cleanly arresting at the nitroso state when using P^III^-based oxygen atom acceptors. We reasoned that the efficiency of these dark reactions would be contingent on the nucleophilicity of the P^III^ center and that electron-poor P^III^ atoms should react more slowly with nitrosoarenes relative to more electron-rich congeners.

**Scheme 2:**
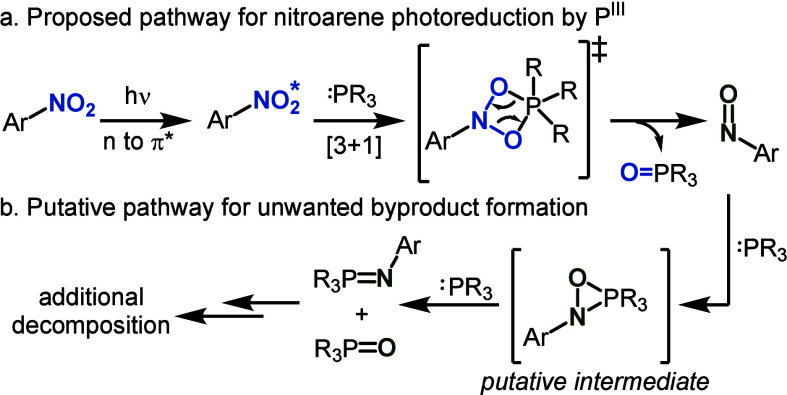
P^III^-Mediated Generation and Decomposition of Nitrosoarenes

To narrow the set of phosphines evaluated as oxygen atom acceptors, we first assayed the thermal reactivity of a panel of commercially available phosphines with nitrosobenzene (**1a**, Table 1). Each phosphine (2 equiv) was stirred with **1a** in THF, a representative polar aprotic solvent, at 23 °C for 16 h under N_2_. Residual nitrosobenzene was then trapped as **1b**, the [4 + 2] cycloadduct with 1,3-cyclohexadiene. This trapping protocol cleanly quenches the reduction and enables convenient evaluation by ^1^H NMR, avoiding resonance overlap among free nitrosoarenes, nitroso dimers, and aromatic phosphines. The cycloaddition yield was independently verified to be >93% under these conditions, validating this approach for approximating the nitrosobenzene concentration.

**Table 1:**
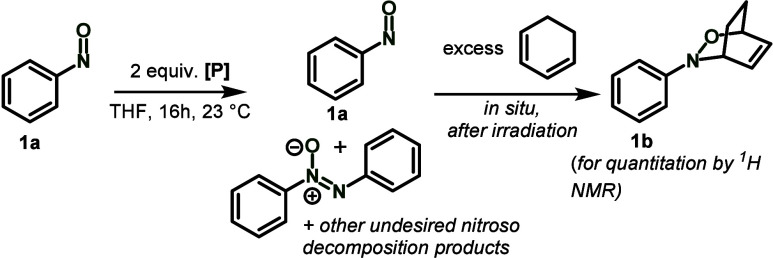
Nitrosobenzene–Phosphine Reactivity Assay

entry	[P]	**1b** by NMR (%)
1	**A**	92
2	**B**	81
3	**C**	<2
4	**D**	28
5	**E**	14
6	**F**	<2
7	**G**	93
8	**H**	31
9	**I**	<2
10	**J**	91

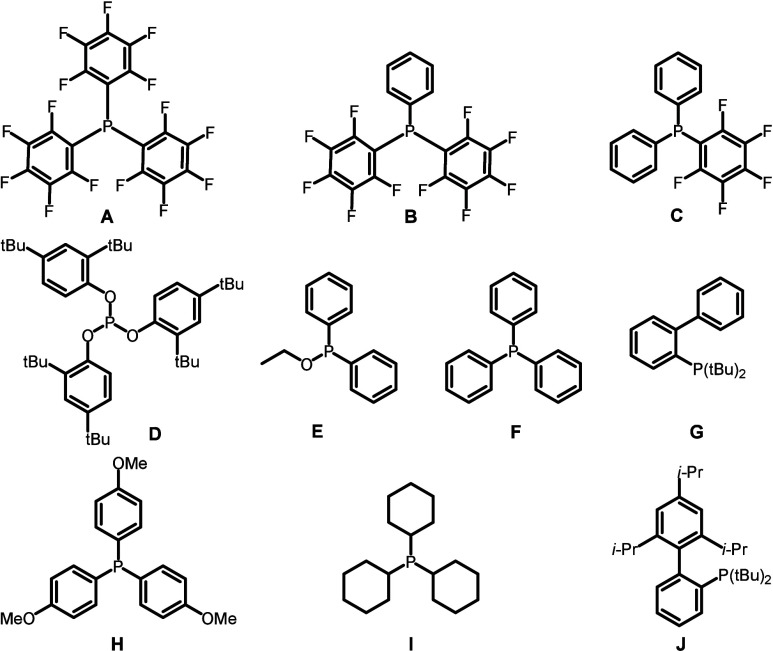

The assay is consistent with the expected stereoelectronic trends for nitrosoarene degradation processes initiated by nucleophilic activation of nitrosobenzene by P^III^ ([P], Table 1). Electron-poor P^III^ centers, including perfluorophenyl-substituted phosphines (**A** and **B**), reacted slowly or not at all with nitrosobenzene in the dark. In contrast, electron-neutral phosphines (e.g., **F**) and electron-rich phosphines (e.g., **I,** PCy_3_) consumed nitrosoarene, producing aniline and azoxybenzene as the primary products detected by GC–MS. Steric congestion also attenuated this reactivity regardless of electronics: electron-rich but highly hindered biaryl phosphines **G** and **J** both failed to decompose nitrosobenzene under these conditions. A similar tolerance of *t*-Bu XPhos toward O_2_ reduction has been attributed to steric inhibition of irreversible decomposition through transition states involving two phosphines.^58^

Having identified phosphines that resist side reactions with nitrosobenzene, we next evaluated the competency of these phosphines in photomediated deoxygenation of nitroarenes. We focused our studies on phosphines that preserved at least 80% of the starting nitrosobenzene over the course of the 16 h assay. Phosphines **A**, **B**, **D**, **G**, and **J** were irradiated at 365 nm with model nitroarene, 4-nitrobiphenyl (**2a**), for 6 h in EtOAc.^59^ Again, the product nitrosoarene was trapped as a cycloadduct after irradiation. As shown in Table 2, all phosphines except **G** afforded significant quantities of cycloadduct **2c**, with phosphine **B** performing the best. Further analysis of the crude reaction mixture showed the presence of the corresponding phosphine oxide of **B**, supporting the proposed role of **B** as an oxygen atom acceptor.^60^ Control reactions involving the exclusion of light or phosphine confirmed that both light and phosphine are required to to bring about this deoxygenation reaction.

**Table 2:**
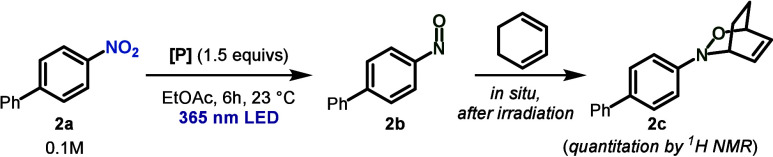
Photoreduction Efficacy across Phosphines

entry	[P]	**2c** yield (%)
1	**A**	13
2	**B**	56
3	**D**	31
4	**G**	<2
5	**J**	27
6	no [P]	<2
7	**B** (no light)	<2

The reaction was further optimized by evaluating the solvent, irradiation wavelength, and reaction time (Table 3). Polar aprotic solvents, such as dioxane and fluorobenzene, performed comparably to ethyl acetate but are less environmentally friendly; therefore, EtOAc was retained as the solvent of choice (entries 7 and 9). The irradiation wavelength had a larger effect. Cycloadduct yields increased across wavelengths from 365 to 390 and 420 nm but decreased sharply at 450 nm, consistent with prior reports and the rapid drop in nitroarene molar absorptivity above 400 nm.^47^ Shorter wavelengths gave higher conversion over shorter time scales but lower yields, consistent with byproduct formation. Reducing the concentration from 0.10 to 0.025 M provided an additional 8% yield increase. Though the origin of this concentration effect is not totally clear, it may be due to changes in light penetration affected by a reduction in the azodioxy dimer equilibrium, which is expected to be more favorable at higher concentrations. Under these conditions, 420 nm irradiation delivered the highest yield for **2c** (93%, entry 2, Table 3). However, subsequent evaluation across additional model substrates showed that 390 nm irradiation afforded higher yields (Table S4). This wavelength was used for the substrate scope, despite the lower yield for conversion of **2a** to **2c**.

**Table 3:**
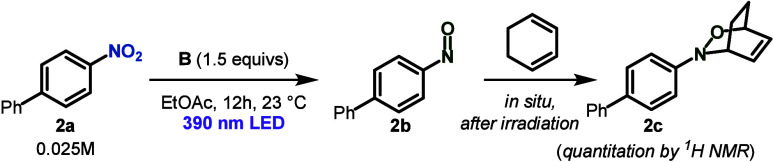
Synthetic Optimization of 4-Nitrosobiphenyl

entry	variation of optimized conditions	**2c** yield (%)
1	none	78
2	420 nm LED	93
3	0.10 M	70
4	0.10 M, 450 nm LED	28
5	0.10 M, 420 nm LED	76
6	0.10 M, 365 nm LED	50
7	0.10 M, 365 nm LED, dioxane	45
8	0.10 M, 365 nm LED, MeCN	17
9	0.10 M, 365 nm LED, PhF	52
10	**A** instead of **B**, 0.10 M, 365 nm	28
11	no light	<2
12	no **B**	<2

With optimized conditions in hand, we evaluated the generality of the photomediated nitroarene-to-nitrosoarene conversion across a range of electronically and sterically diverse substrates (Scheme 3). It should be noted that because nitrosoarenes generally reversibly dimerize at room temperature, isolated compounds are likely to be azodioxy dimers of nitrosoarenes and not the free nitroso. The reaction tolerates electron-neutral to -rich aryl halides (**3b**, **8b**, and **15b**), ethers (**4b** and **5b**), a boronate ester (**6b**),^61^ and an unprotected aliphatic alcohol (**10b**), furnishing the corresponding nitrosoarenes in moderate to good yields. Notably, *ortho*-methyl substrate **9b** could be isolated in a good yield. Nitroarenes with *ortho*-alkyl groups can undergo H atom transfer with subsequent decomposition to other products. Heterocycles, including benzothiazole (**16b**), pyrrole (**17b**), and indole (**19b**) derivatives, were furnished in moderate to good yields as well.

**Scheme 3:**
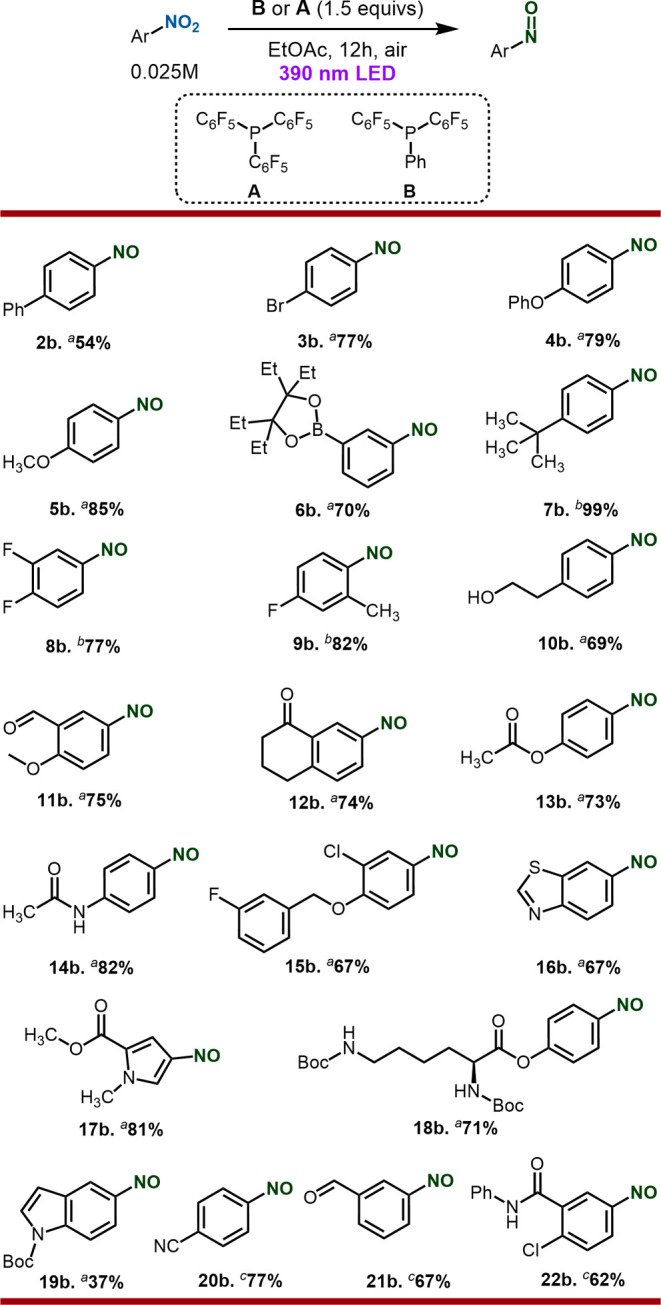
Substrate Scope of Nitroarenes

a General procedure A: nitroarene (0.50 mmol, 1.0 equiv) and **B** (0.75 mmol, 1.5 equiv) were irradiated with a 390 nm LED for 12 h in EtOAc (20.0 mL).
b Due to the volatility of these products, these compounds were synthesized through procedure A and then isolated as the cycloadduct with 1,3-cyclohexadiene.
c General procedure B: same as procedure A, except that phosphine **A** was used instead.

Some nitroarenes with strong electron-withdrawing groups were not well-tolerated under the standard conditions with phosphine **B**, despite the complete consumption of starting materials. Instead, these substrates gave complex product mixtures consistent with the activation of the nitrosoarene product by phosphine. We hypothesized that electron-withdrawing substituents increase the susceptibility of nitrosoarene to dark reactions with phosphine **B**. To address this scope limitation, we evaluated phosphine **A** as an alternative terminal oxygen atom acceptor. The anticipated lower nucleophilicity of **A** relative to that of **B** should mitigate any background reactions involving nucleophilic activation of the nitrosoarene product. Indeed, replacing **B** with **A** improved the yields of electron-poor nitroso products. Notably, a nitrile (**20b**), unprotected aldehyde (**21b)**, and an amide (**22b**) were tolerated in moderate to good yields under these conditions. Such functional groups are potentially reactive under reductive routes to nitrosoarenes or hydroxylamines.^62^ As such, this method complements the existing approaches.

We reasoned that the relatively unreactive byproducts of the reaction, namely, the phosphine oxide of **B**, would minimally interfere with subsequent elaboration reactions of nitrosoarenes. As such, we next evaluated the synthetic applications of this approach in multi-step, one-pot derivatization sequences. Following photochemical deoxygenation via procedure A and subsequent derivatization reactions, nitroarene **3a** was converted into an *N*-aryl 1,4-amino alcohol (compound **23**, Scheme 4a),^63^ a diaryl amine via phosphite-mediated C–N coupling (compound **24**, Scheme 4b),^64,65^ a hetero-Diels–Alder cycloadduct (compound **25**, Scheme 4c), and an unsymmetric azo-arene via condensation with aniline (compound **26**, Scheme 4d).^32^ As shown in Scheme 4, these transformations proceeded in moderate yields (39–64%) without optimization or purification between steps. These results highlight the utility of this approach for accessing structurally diverse nitrosoarene-derived products.

**Scheme 4:**
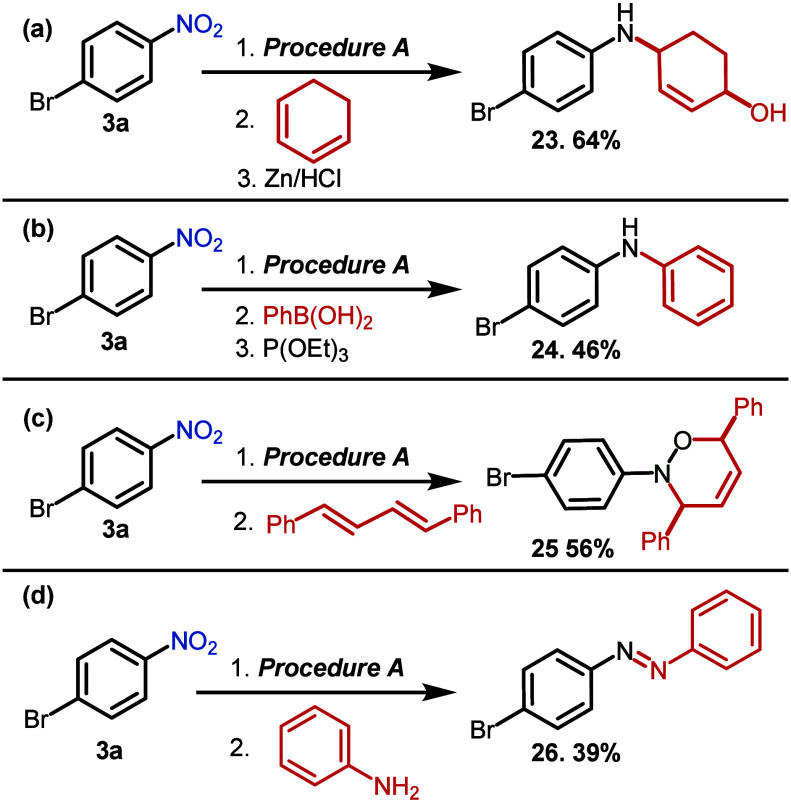
Synthetic Applications

In summary, this paper describes the development of a photomediated deoxygenation of nitroarenes that arrests cleanly at the nitroso oxidation state. With a careful choice of the P^III^ oxygen atom acceptor, this approach circumvents over-reduction and condensation pathways that sometimes limit access to this motif. Electron-poor phosphines serve as competent oxygen atom acceptors under photoexcitation yet are thermally inert toward the nitroso product. The reaction tolerates a wide range of functionality, including boronate esters, aliphatic alcohols, Boc-protected amines, and select heterocycles. Tailoring the phosphine to mitigate potential interactions of the nitrosoarene products extends the scope to substrates bearing strongly electron-withdrawing substituents, such as nitriles and unprotected aryl aldehydes. A limitation of this approach is the necessity of specialized fluoroaryl phosphines. Although these were commercially available at the time of this study, the failure of less expensive phosphines to efficiently mediate this reaction is a significant challenge facing the larger scale implementation of this approach. Nonetheless, we expect that this mild approach will improve access to nitrosoarenes in discovery chemistry contexts and expand their use in the synthesis of complex molecules and materials.

## Supplementary Material



## Data Availability

The data underlying this study are available in the published article and its Supporting Information.

## Notes

Additional experimental details, characterization, and methods, including photographs of the photoreactor (PDF)
