# Supplementary material for: One-Step Photoreduction of Nitroarenes to Nitrosoarenes
Source: Org Lett. 2026 Jun 25;28(27):8576–81. doi: 10.1021/acs.orglett.6c02016 (PMC13366691; doi:10.1021/acs.orglett.6c02016)
Supplement: Supplementary file 1 [file ol6c02016_si_001.pdf]

Supporting Information

One-Step Photoreduction of Nitroarenes to Nitrosoarenes

Justice A. Keech, Narva Deshwar Kushwaha, James R. Bour*

Department of Chemistry, Wayne State University, 5101 Cass Ave. Detroit, MI 48202

E-mail: bour@wayne.edu

Table of Contents

1. General Information ... S2

2. Safety Considerations ... S2

3. Materials & Methods ... S2

4. Selection of P^{III} Compounds via Dark Reaction Screening ... S4

5. Photoreduction Using Suitable Phosphines & Phosphites ... S7

6. Optimization of General Nitrosoarene Synthesis ... S9

7. General Procedure A ... S11

8. General Procedure B ... S11

9. Synthetic Applications ... S12

10. Recovery of Phosphine Oxides ... S13

11. 3D-Printed Photoreactor Setup ... S14

12. Optimization of LED Wavelength ... S15

13. Limiting Factors ... S16

14. Characterization Data ... S17

15. References ... S30

16. NMR Spectra ... S31

1. General Information

Thin layer chromatography (TLC) was performed using Sorbtech silica G w/UV254 thin layer plates. All final compounds (nitrosoarenes or cycloadducts) were purified by column chromatography using Combi*F*lash Nextgen 300. NMR spectra were obtained on Bruker AV NMR (500 MHz for ^{1}H and 125 MHz for ^{13}C) and an Agilent DD2-600 MHz (600 MHz for ^{1}H) spectrometer. ^{1}H NMR chemical shifts are reported in parts per million (ppm) relative to tetramethylsilane (TMS), with sample residual solvent peak used as an internal reference. Abbreviations used to report the NMR data: br, broad resonance; s, singlet; d, doublet; t, triplet; q, quartet; p, quintet; h, sextet; sept, septet; dd, doublet of doublets; dt, doublet of triplets; m, multiplet, dm doublet of multiplets. High-resolution mass spectrometry was performed by the Lumigen Instrument Center, Wayne State University. High-resolution mass spectra (HRMS) were acquired using atmospheric pressure chemical ionization (APCI) on an orbitrap mass spectrometer. Positive mode electrospray ionization (ESI) was used on an orbitrap mass spectrometer for compounds **23-26**. Gas chromatography/mass spectrometry using 70 eV electron impact (EI) ionization was utilized for **3b**, due to difficulty with other ionization techniques. Melting points were acquired using a Thermo Scientific MEL-TEMP 3.0. Abbreviations used for chemicals: DMSO (dimethyl sulfoxide), DCM (dichloromethane), MeCN (acetonitrile), EtOAc (ethyl acetate), THF (tetrahydrofuran), PhF (fluorobenzene).

2. Safety Considerations

Nitro and nitroso compounds are potentially toxic, carcinogenic, energetic, and may exhibit thermal instability. All manipulations were performed on a small scale inside of a fume hood with appropriate personal protective equipment. Newly synthesized compounds should be treated as having unknown toxicological properties.

3. Materials and Methods

3.1. Materials

Unless stated otherwise, all commercial reagents were used as received, except for 4,4,5,5-tetraethyl-2-(3-nitrophenyl)-1,3,2-dioxaborolane (**6a**), which was synthesized according to the literature.[1] Bis(perfluorophenyl)(phenyl)phosphine and Tris(perfluorophenyl)phosphine were

purchased from Ambeed, Inc. and stored at room temperature in an N_2-filled glovebox. Nitroarenes were purchased from Ambeed, Inc., Combi-Blocks, and Oakwood Chemical, ensuring proper storage conditions based on chemical labels. 1,3-cyclohexadiene was purchased from Combi-Blocks and stored at 2-8 °C. Ethyl acetate (EtOAc) was HPLC grade and purchased from Fisher Chemicals. Aluminum-backed 200 µm particle size thin layer chromatography (TLC) plates were purchased from Sorbtech; RediSep Silver Silica Gel Disposable Flash Columns (24 gram; 40-60 µm particle size; 230-400 mesh size) were purchased from Teledyne ISCO.

3.2. Photoreactors

Reactions using 420 nm and 365 nm light were conducted inside a Penn Photoreactor, stirring at 500 rpm at 75% intensity. Fan speed was set to 1500 rpm to ensure reaction vials were adequately cooled. Note that the efficiency of this cooling mechanism was verified by checking the temperature of a representative sample via multimeter after irradiation for 6 h. In two separate tests, probes were placed inside and outside of reaction vials. The temperatures did not exceed 28 ºC over this period. Reactions at 390 nm were conducted in the 3D printed photoreactor outlined in section 11,[2] using lab air for cooling and stirring at 500 rpm. Light intensity was set to 75%. This reactor showed similar results (+/-5%) to the Penn reactor in a head-to-head comparison at 365 nm. Temperatures were measured in an identical temperature test, with the temperatures inside and outside of the vials not exceeding 30 ºC over a 12 h period.

3.3. ^{1}H NMR Yield Determination

To determine substrate viability and the amount of surviving nitrosobenzene (**1a**) in solutions of phosphines and phosphites, 1,3-cyclohexadiene nitroso Diels-Alder adduct yields were quantified using ^{1}H NMR. 32 scans were performed for each species on an Agilent DD2-600 MHz using a 90º pulse angle and 5 s relaxation delay. Integration of alkene resonances relative to a 1 equiv. 1,3,5-trimethoxybenzene standard indirectly gave the concentration of aryl nitroso species present in solution at the end of the reaction. More information can be found in section 4.

4. Selection of P^{III} Compounds via Dark Reaction Screening

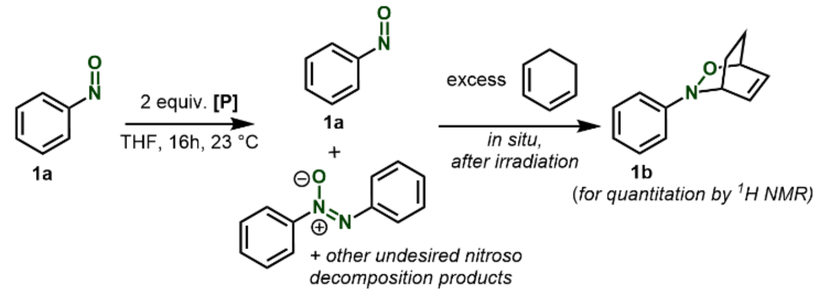

Development of the cycloadduct trapping assay. Direct quantitation of aryl nitroso species in solution by ^{1}H NMR is complicated by their complex equilibria with cis and trans dimers and signal overlap with other aromatic species in solution. As such, we targeted an assay that exploits the efficient reactivity of nitrosoarenes with 1,3-cyclohexadiene to form cycloadducts with well resolved alkene resonances that could be quantified by ^{1}H NMR. The efficiency of the cycloadduct trapping was verified through the following procedure. Under air, a 4 mL vial was charged with a 1 mL of a 0.05 M solution of nitrosobenzene in THF (0.05 mmol) and a 6 mm magnetic stir bar. The solution was stirred for one minute before 3 drops of 1,3-cyclohexadiene were added via pipette. The solution was stirred at 23 ºC for 1 h before the volatile solvent was evaporated in vacuo using a vacuum oven (23 ºC, ~2 torr). Next, 1 mL of a 0.05 M 1,3,5-trimethoxybenzene solution in $CDCl_3$ (0.05 mmol) was added to the sample before transfer to an NMR sample tube. Quantitation by NMR indicated that the cycloadduct was formed in a yield of 93% (Figure S1) against the internal standard. Because this assay is meant to give approximate stabilities of nitrosobenzene to various phosphines, this efficiency was sufficient for determining the viability of P^{III} species for photolytic experiments.

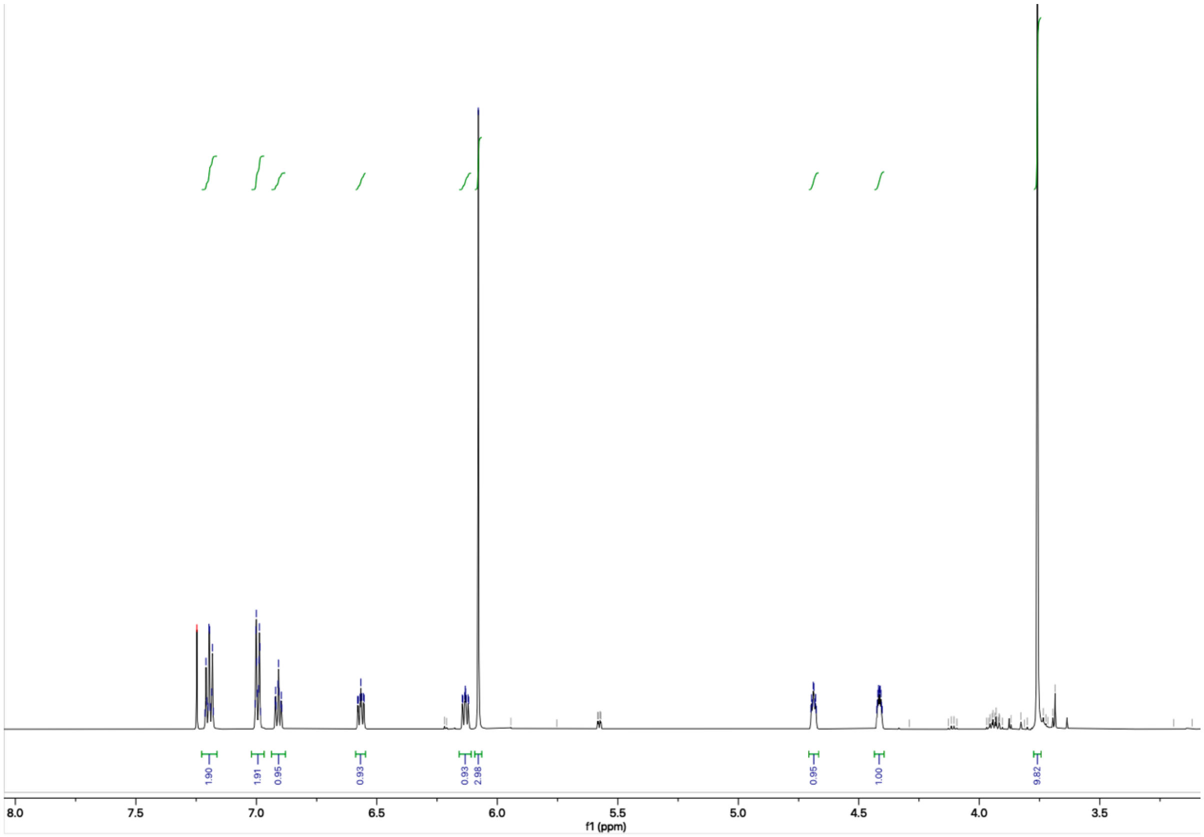

Figure S1: ^{1}H NMR spectra of nitrosobenzene cycloadduct with 1,3-cyclohexadiene (**1c**). Note relative integrals of 1,3,5-trimethoxybenzene (6.08 ppm) and **1b** alkene resonances (6.13 ppm & 6.57 ppm) showing 93% yield.

Nitrosobenzene stability assay. With a quick assay for measuring nitrosobenzene in solution in hand, we next evaluated the reactivity between nitrosobenzene and a panel of commercially available P^{III} compounds using the following procedure. To 4 mL vials containing 0.1 mmol of each P^{III} compound (2 equiv.), 1 mL of a 0.05 M nitrosobenzene/THF solution (0.05 mmol, 1 equivalent) was added and allowed to sit for 16 h at 23 ºC. Next, 3 drops of 1,3-cyclohexadiene (~30 mg, 0.38 mmol, 6 equiv.) were then added to each vial and allowed to stir for 1 h before concentrating in vacuo using a vacuum oven (23 ºC, ~2 torr) until a sticky residue was obtained. 1 mL of 0.05 M 1,3,5-trimethoxybenzene in $CDCl_3$ was added to the vials before analysis by ^{1}H NMR. Because of the efficiency of the cycloadduct trapping, the yield of the cycloadduct (**1b**) was taken to be an approximate measure of nitrosobenzene (**1a**) remaining at the end of the reaction with the P^{III} compound. The results of this assay are summarized below.

Table S1. Stability of Nitrosobenzene in the Presence of P^{III} Compounds

entry	P^{III} Compound	% 1b by NMR
**1**	**A**	92
**2**	**B**	81
**3**	**C**	<2
**4**	**D**	28
**5**	**E**	14
**6**	**F**	<2
**7**	**G**	93
**8**	**H**	31
**9**	**I**	<2
**10**	**J**	91
**11**	**K**	<2
**12**	**L**	<2
**13**	**M**	<2
**14**	**N**	<2
**15**	**O**	16
**16**	**P**	<2
**17**	**Q**	<2
**18**	**R**	<2
**19**	**S**	24

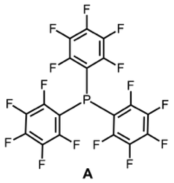
A

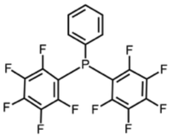
B

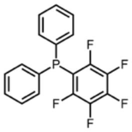
C

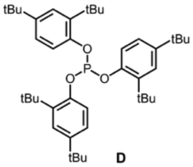

D

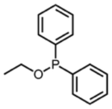
E

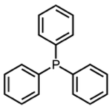
F

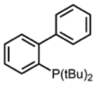

G

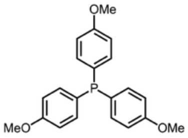

H

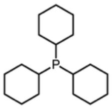
I

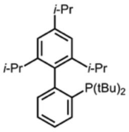

J

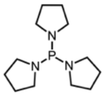
K

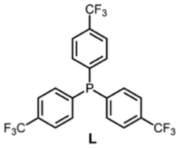

L

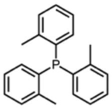
M

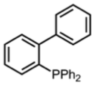

N

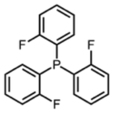
O

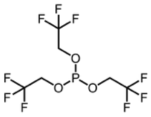
P

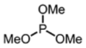

Q

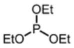

R

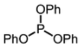

S

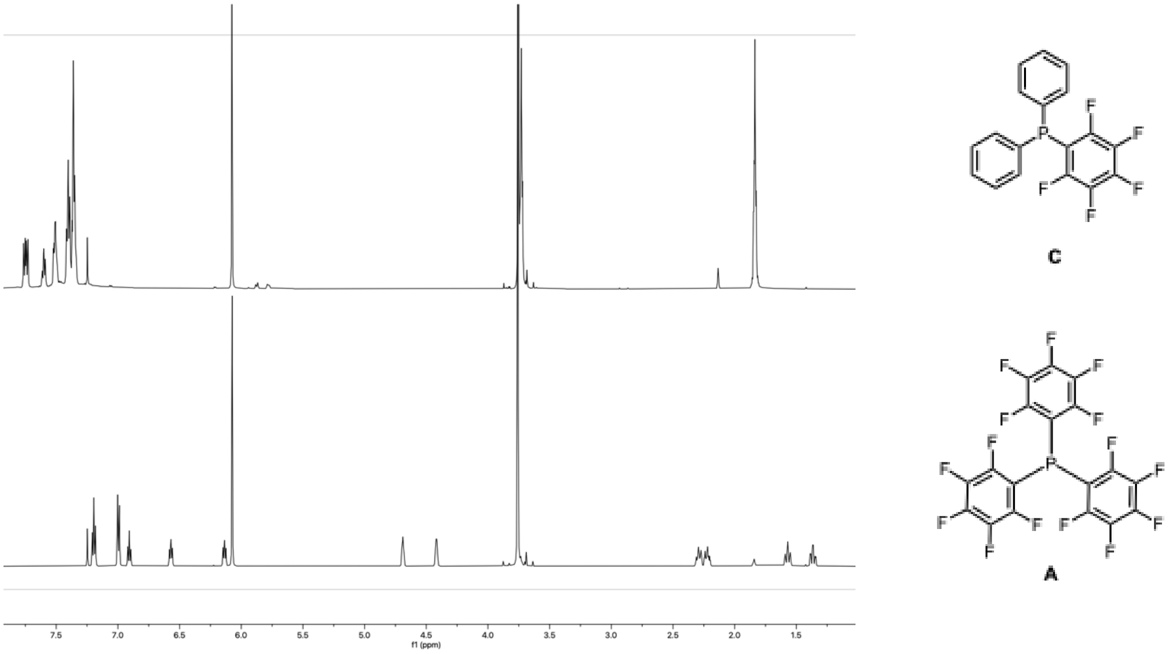

Figure S2: ^{1}H NMR spectra of decomposed nitroso in the presence of phosphine **C** (top) and the nitroso Diels-Alder adduct of surviving nitroso in the presence of phosphine **A** (bottom). Note distinctive characteristic cycloadduct resonances between 6.0ppm-6.75ppm and 4.25ppm-4.75ppm for **A**.

5. Photoreduction Using Suitable P^{III} Oxygen Atom Acceptors

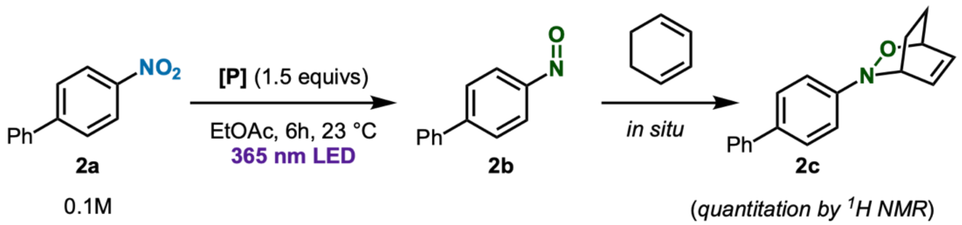

Using the P^{III} compounds that resulted in the least nitroso decomposition, photoreduction was attempted with each to determine their viability for nitrosoarene synthesis. 4 mL vials equipped with magnetic stir bars were charged with 4-nitrobiphenyl (**2a**) (0.05 mmol, 1.0 equiv.). P^{III} compounds **A**, **B**, **D**, **G**, and **J** (0.075 mmol, 1.5 equiv.) were added to their respective vials with EtOAc (2.0 mL each). The vials were sealed and irradiated at 365 nm in a photoreactor under ambient atmosphere with stirring and lab air flow for cooling. After 6 h, the reaction mixture was treated with 3 drops of 1,3-cyclohexadiene, and solvent was evaporated in a vacuum oven. Vials were charged with 1,3,5-trimethoxybenzene (0.05 mmol, 1 equiv.) via stock solution in $CDCl_3$ for determination of the ^{1}H NMR yield.

Table S2. % Yield of 2c under Photolytic Conditions

entry	[P]	2c yield (%)
1	**A**	13
2	**B**	56
3	**D**	31
4	**G**	<2
5	**J**	27
6	None	<2
7	**B**, (no light)	<2

Nitroarene **2a** (10.0 mg, 0.05 mmol, 1.0 equiv.) and **A**, **B**, **D**, **G**, and **J** (0.075 mmol, 1.5 equiv.) were irradiated with 365 nm LED for 6 h in EtOAc (2.0 mL) with stirring and lab air for cooling; 3 drops 1,3-cyclohexadiene were added upon reaction completion and vials were dried in a vacuum oven. ^{1}H NMR yield of cycloadduct was determined against a 1,3,5-trimethoxybenzene standard.

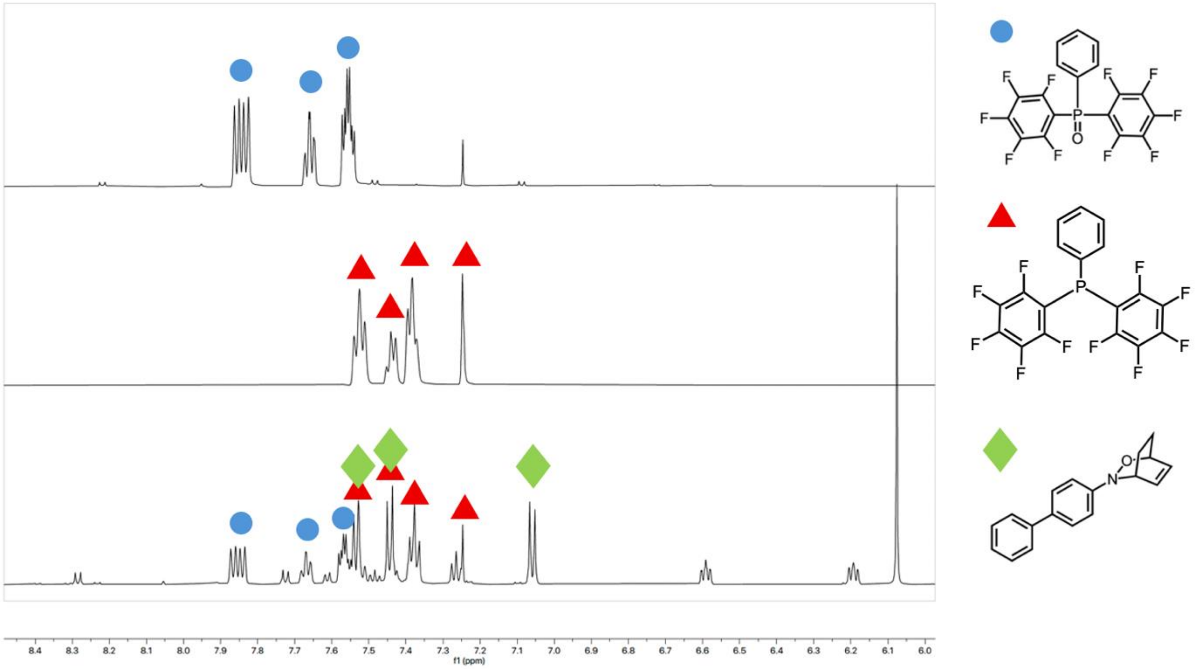

Figure S3: ^{1}H NMR spectra of phosphine **B** oxide (top), phosphine **B** (middle), and a crude reaction mixture containing both (bottom).

6. Optimization of General Nitrosoarene Synthesis

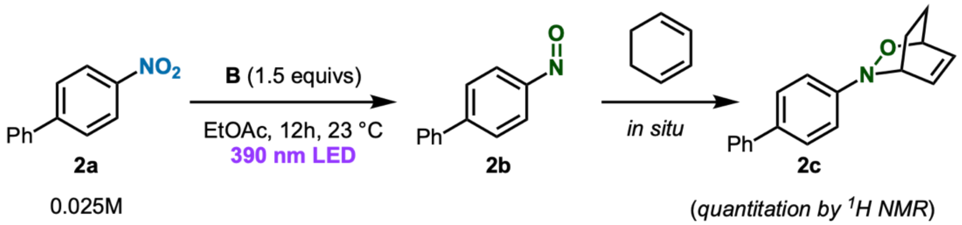

Initial experiments using 0.05 mmol of nitroarene and 0.075 mmol of phosphine in 4 mL vials showed promise at a concentration of 0.1 M under 365 nm LEDs. However, yields were lower than desired. After probing the effects of different solvents and wavelengths, 12-hour 420 nm trials using EtOAc were determined to be the highest yielding reactions for **2a**. Solvents shown in the table below were selected for their lack of weak C–H bonds, which cause unwanted side reactivity. Varying concentrations were later tested, with lower concentrations (0.025 M & 0.05 M) proving to be the most fruitful. These conditions were very effective for conversion of **2a** to **2b**, but after applying them to other substrates, it was determined that they were not as widely applicable as expected. Use of a 390 nm light source in the reactor outlined in **S3** proved to be the most activating across the entire scope of nitroarenes and was used for all substrates moving forward.

Table S3. Optimization of 2c Yields

entry	variation of optimized conditions	yield 2c (%)
1	none	78
2	420 nm LED	93
3	0.10 M	70
4	0.10 M, 450 nm LED	28
5	0.10 M 420 nm LED	76
6	0.10 M 365 nm LED	50
7	0.10 M, 365 nm LED, Dioxane	45
8	0.10 M, 365 nm LED, MeCN	17
9	0.10 M, 365 nm LED, PhF	52
10	**A** instead of **B**, 0.10 M, 365 nm	28
11	No light	<2
12	No **B**	<2

Nitroarene **2a** (10.0 mg, 0.05 mmol, 1.0 equiv.) and **B** bis(perfluorophenyl)phenyl phosphine (0.075 mmol, 1.5 equiv.) were irradiated with LED lights in varying solvents and concentrations with stirring and air flow for cooling; 3 drops 1,3-cyclohexadiene were added upon reaction completion and vials were dried in a vacuum oven. ^{1}H NMR yield of cycloadduct was determined against a 1,3,5-trimethoxybenzene standard.

7. General Procedure A:

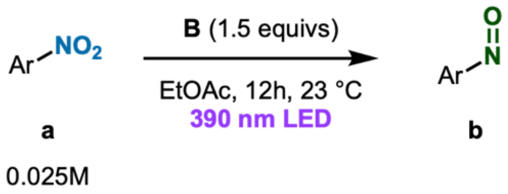

A 20 mL vial equipped with a magnetic stir bar was charged with nitroarene (0.50 mmol, 1.0 equiv.), **B** bis(perfluorophenyl)phenyl phosphine (331.6 mg, 0.75 mmol, 1.5 equiv.), and EtOAc (20.0 mL). The vial was sealed and irradiated at 390 nm (75% intensity) in a photoreactor (*vide infra*) under ambient atmosphere with stirring and lab air flow for cooling. After 12 h, the reaction mixture was transferred to a round-bottom flask containing 3 g of silica powder before concentrating to dryness using rotary evaporation. Product-adsorbed silica was then loaded onto a CombiFlash column using the solid-loading method. Flash column chromatography was performed over 40-50 minutes using a gradient of 0-40% EtOAc in hexanes. All nitroso products eluted between 0.5-25% EtOAc in hexanes. Fractions containing the desired product, as determined by TLC analysis, were combined and concentrated under rotary evaporation to afford the title product.

8. General Procedure B:

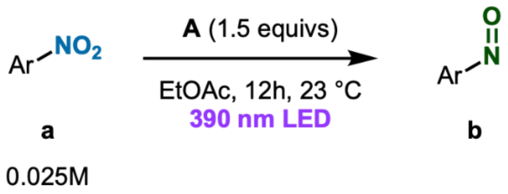

A 20 mL vial equipped with a magnetic stir bar was charged with nitroarene (0.50 mmol, 1.0 equiv.), **A** tris(perfluorophenyl)phosphine (399.1 mg, 0.75 mmol, 1.5 equiv.), and EtOAc (20.0 mL). The reaction vial was sealed and irradiated at 390 nm (75% intensity) in a photoreactor (*vide infra*) under ambient temperature for 12 h with continuous stirring and lab air flow for cooling. After 12 h, the reaction mixture was transferred to a round-bottom flask containing 3g of silica powder before concentrating to dryness using rotary evaporation. Product-adsorbed

silica was then loaded onto a CombiFlash column using the solid-loading method. Flash column chromatography was performed over 40-50 minutes using a gradient of 0-40% EtOAc in hexanes. The products eluted between 0.5-25% EtOAc in hexanes. Fractions containing the desired product, as determined by TLC analysis, were combined and concentrated under rotary evaporation to afford the title product.

9. Synthetic Applications

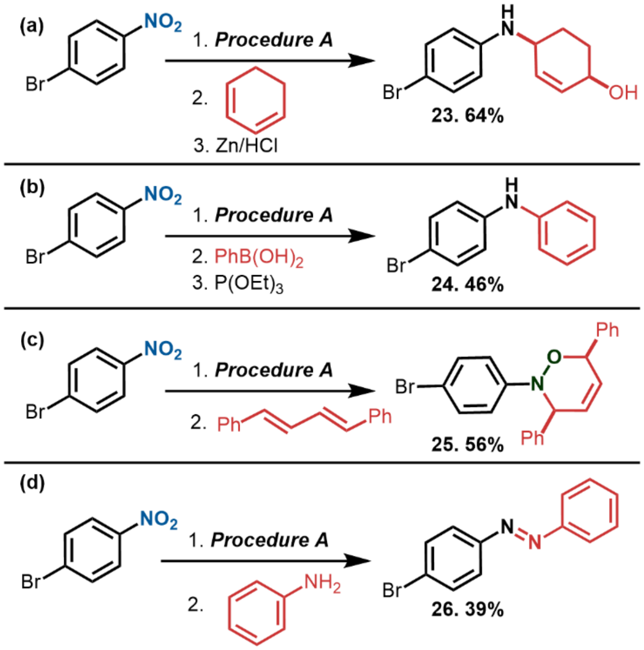

Treatment of nitroarenes using procedure A was followed with several subsequent transformations to emphasize the utility of this method. In one reaction vial containing crude **3b**, was added five drops of 1,3-cyclohexadiene. After one hour, the volatiles were removed using rotary evaporation before addition of DCM (5 mL), glacial acetic acid (5 mL), and water (5 mL). Zn powder (653.9 mg, 10 mmol, 20 equiv.) was then added and the mixture stirred at 500 rpm for 2 h at room temperature. The crude product was then washed with saturated aqueous $NaHCO_3$ (10 mL x 3) and saturated aqueous NaCl (10 mL x 3), before drying with Na_2SO_4 and concentrating via rotary evaporation. 86 mg of **23** was isolated using flash column chromatography, representing a 64% yield.

Another vial containing the same crude nitroso product was concentrated to an oil via rotary evaporation and dissolved in THF (1.07 mL), before addition of phenylboronic acid (91.4 mg,

0.75 mmol). Triethylphosphite (102 μL, 0.6 mmol) was then added, and the mixture was left to stir at 500 rpm for 4 h at room temperature. The mixture was concentrated to dryness via rotary evaporation and 57 mg of C–N coupling product **24** was isolated using flash column chromatography, representing a 46% yield.

The synthesis of **25** began with removal of ~10 mL of EtOAc from the **3b** crude reaction mixture using a gentle stream of N_2. Next, (1E,3E)-1,4-diphenylbuta-1,3-diene (123.8 mg, 0.6 mmol, 1.2 equiv.) was added and left to stir at 500 rpm under ambient temperature for 24 h. The mixture was then concentrated to dryness via rotary evaporation and **25** was isolated using flash column chromatography affording 110 mg of product representing a 56% yield.

Lastly, another crude reaction mixture of **3b** was concentrated to dryness via rotary evaporation, before addition of ethanol (0.3 mL), glacial acetic acid (1.2 mL), and aniline (55 μL, 0.6 mmol, 1.2 equiv.). The reaction mixture was stirred at 500 rpm for 16 h at 40 ºC. This mixture was then concentrated to dryness via rotary evaporation and **26** was isolated via flash column chromatography, affording 51 mg of product representing a 39% yield.

More information on product elution and chromatography mobile phase gradients for **23**, **24**, **25**, and **26** can be found in the characterization data section (S23-S24).

10. Recovery of Phosphine Oxides

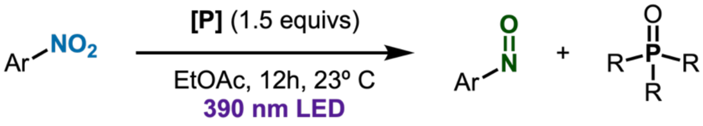

To explore cost mitigation of phosphine **B** use, its oxide (**S1**) was isolated (220 mg, 64% yield) from the crude nitroso mixture via flash chromatography from the reduction of **5a**. The recovery of the phosphine oxide is also consistent with the role of the phosphine reagent as a reductant and oxygen acceptor during the conversion of the nitro substrate. The isolated phosphine oxide could, in principle, be reduced by known methods, which may partially mitigate reagent cost. See page S29 for isolation and characterization data.

11. 3D-Printed Photoreactor Setup

Using the design outlined by the Aubineau group,[2] the photoreactor used in these experiments was printed using a Bambu P1S 3D printer equipped with eSUN brand 1.75 mm PLA+ filament. The inside of the reactor frame was then covered in reflective tape and mounted onto an IKA brand hot plate/magnetic stirrer. A PR160L-390 nm Kessil LED lamp (55W max, used at 75% max intensity) was fitted into the mount, and a small hose was connected to a lab air source and fed into the reactor chamber for cooling. The reactions were conducted in 4 mL (NMR scale) or 20 mL glass scintillation vials which were stirred at 500 rpm. Due to the potential background reactivity of nitrosoarenes with residual phosphines, the reactions were initiated on a timer such that they were worked up immediately following irradiation.

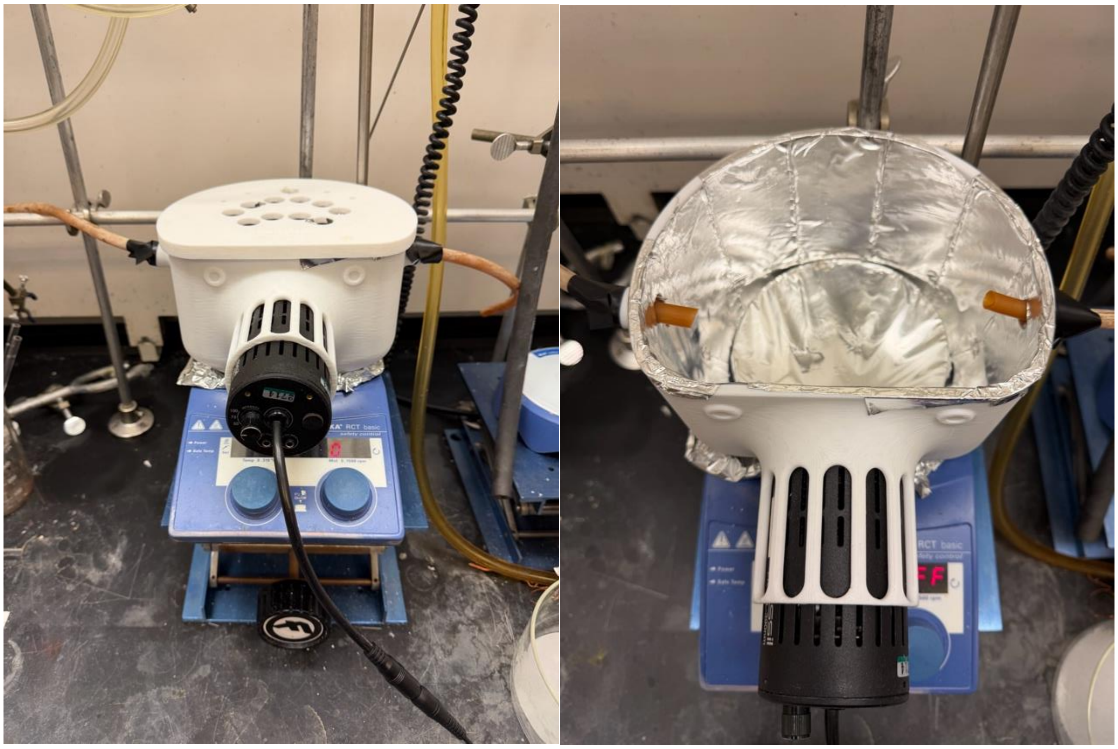

Figure S4: 3D-printed photoreactor setup mounted on an IKA hotplate and fitted with a 390 nm Kessil LED. Note lab-air ingress on left side of reactor, egress on the right.

12. Optimization of LED Wavelength

To determine the effects of different wavelengths of light on functional groups of differing electronic character, a model reaction was performed for three substrates at both 420 nm and 390 nm.

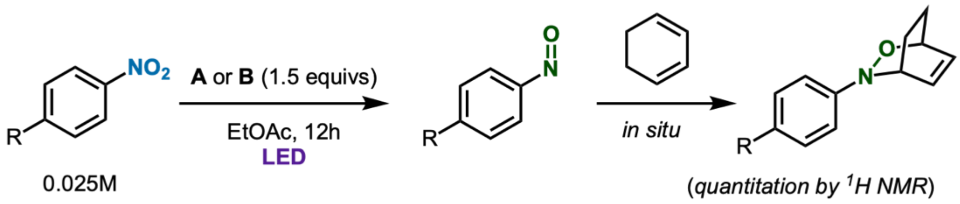

Table S4. Wavelength Effects on Different Functional Groups

Entry	Phosphine [P]	R	Wavelength (nm)	% Yield
**1**	[a]**A**	CN	420	37
**2**	**B**	OMe	420	15
**3**	**B**	Ph	420	93
**4**	[a]**A**	CN	390	77
**5**	**B**	OMe	390	52
**6**	**B**	Ph	390	78

[a] Phosphine selected due to preferable electronic interactions with noted substrate

Nitroarenes **2a**, **5a**, and **21a** (0.05 mmol, 1.0 equiv.) and either **A** tris(perfluorophenyl) phosphine (0.75 mmol, 1.5 equiv.) or **B** bis(perfluorophenyl)phenyl phosphine (0.075 mmol, 1.5 equiv.) were irradiated with either 420 nm or 390 nm LED for 12 h in EtOAc (2.0 mL) with stirring and lab air for cooling; 3 drops 1,3-cyclohexadiene were added after reaction completion and vials were dried in a vacuum oven. ^{1}H NMR yield of cycloadduct was determined against a 1,3,5-trimethoxybenzene standard.

13. Limiting Factors

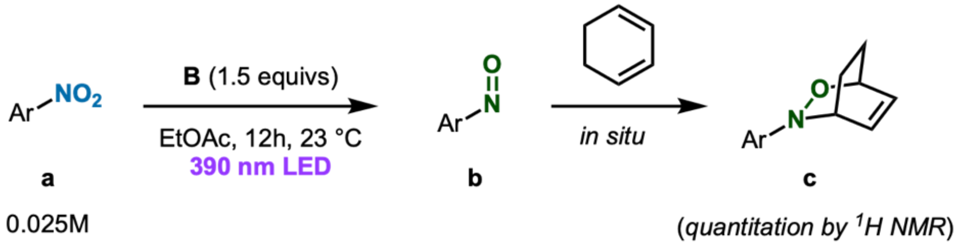

The optimized conditions were ineffective for the following substrates

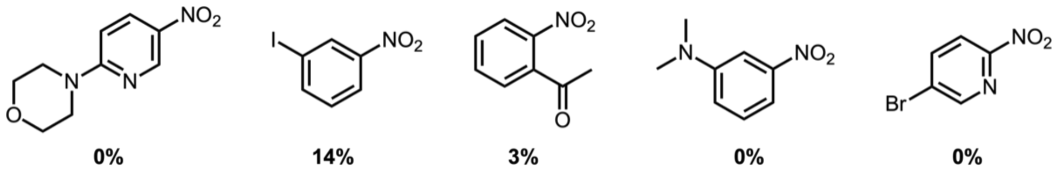

Pyridines were particularly challenging despite high conversions of the starting nitro-pyridine derivatives. Unprotected primary and secondary amines were not widely evaluated based on their anticipated reactivity with the nitroso products. Interestingly, tertiary aromatic amines on the nitroaromatic ring were also poorly tolerated despite low likelihood of reacting directly with nitroso products. Select ortho substituted motifs and select aryl iodides also resulted in low yields. The mechanistic basis for these failures is not clear.

14. Characterization Data

4-nitroso-1,1'-biphenyl **(2b) – $C_{12}H_9NO$**:

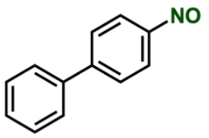

Purification was performed by CombiFlash column chromatography on silica gel using a gradient of 0-10% EtOAc/hexanes over 30 minutes. The product eluted at 4% EtOAc/hexanes, affording a yellow solid (49 mg, 54% yield) upon rotary evaporation; Melting point: 71.3 ºC-72.7 ºC. TLC: 10% EtOAc in hexanes, R_f = 0.4; visualized with UV. ^{1}H NMR (500 MHz, $CDCl_3$, 25 °C): δ 7.99 (d, J = 8.5 Hz, 2H), 7.84 (d, J = 8.7 Hz, 2H), 7.68 (dd, J = 8.3, 1.4 Hz, 2H), 7.56 – 7.49 (m, 2H), 7.49 – 7.41 (m, 1H). ^{13}C NMR (125 MHz, $CDCl_3$, 25 °C): δ 165.2, 148.3, 139.4, 129.3, 129.1, 128.0, 127.6, 121.8. HRMS (APCI) m/z: $[M–H]^-$ calcd for $C_{12}H_8NO$, 182.0611; found 182.0619. Analytical data are consistent with the literature.[3]

1-bromo-4-nitrosobenzene **(3b) – C_6H_4BrNO**:

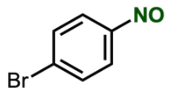

Purification was performed by CombiFlash column chromatography on silica gel using a gradient of 0-5% EtOAc in hexanes over 30 minutes. The product eluted at 0.9% EtOAc in hexanes, affording a yellow solid (72 mg, 77% yield) upon rotary evaporation; Melting point: 98.9 ºC-100.2 ºC. TLC: 5% EtOAc in hexanes, R_f = 0.4; visualized with UV. ^{1}H NMR (500 MHz, $CDCl_3$, 25 °C): δ 7.82 – 7.78 (m, 4H).^{13}C NMR (125 MHz, $CDCl_3$, 25 °C): δ 164.0, 132.9, 131.9, 122.3. GC/MS (EI) m/z: $[M]^{+\bullet}$ calcd for C_6H_4BrNO, 184.95; found 185.00; Due to stability problems during traditional ionization HRMS, GC/MS with 70 eV electron impact ionization was used. Analytical data are consistent with the literature and subsequent elaboration to other products further supports the structural assignment (section 8).[3]

***1-nitroso-4-phenoxybenzene* (4b) – $C_{12}H_9NO_2$:**

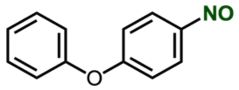

Purification was performed by CombiFlash column chromatography on silica gel using a gradient of 0-10% EtOAc in hexanes over 30 minutes. The product eluted at 1% EtOAc in hexanes, affording a green oil (79 mg, 79% yield) upon rotary evaporation; TLC: 10% EtOAc in hexanes, R_f = 0.5; visualized with UV. ^{1}H NMR (500 MHz, $CDCl_3$, 25 °C): δ 7.87 (d, J = 8.4 Hz, 2H), 7.42 (dd, J = 8.5, 7.4 Hz, 2H), 7.29 – 7.22 (m, 1H), 7.11 – 7.08 (m, 2H), 7.06 – 7.03 (m, 2H). ^{13}C NMR (125 MHz, $CDCl_3$, 25 °C): δ 164.3, 163.9, 154.6, 130.4, 125.5, 124.2, 120.9, 116.8. HRMS (APCI) m/z: $[M–H]^-$ calcd for $C_{12}H_8NO_2$, 198.0561; found 198.0567. Analytical data are consistent with the literature.[4]

***1-methoxy-4-nitrosobenzene* (5b) – $C_7H_7NO_2$**

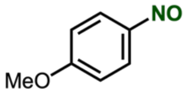

Purification was performed by CombiFlash column chromatography on silica gel using a gradient of 0-50% EtOAc in hexanes over 40 minutes. The product eluted at 19% EtOAc in hexanes, affording a blue oil (58 mg, 85% yield) upon rotary evaporation; TLC: 50% EtOAc in hexanes, R_f = 0.5; visualized with UV. ^{1}H NMR (500 MHz, DMSO-d_6, 25 °C): δ 7.96 – 7.94 (m, 2H), 7.23 – 7.21 (m, 2H), 3.95 (s, 3H). ^{13}C NMR (125 MHz, DMSO-d_6, 25 °C): δ 165.8, 164.1, 124.3, 114.4, 56.3. HRMS (APCI) m/z: $[M–H]^-$ calcd for $C_7H_6NO_2$, 136.0404; found 136.0410. Analytical data are consistent with the literature. [3]

4,4,5,5-tetraethyl-2-(3-nitrosophenyl)-1,3,2-dioxaborolane (**6b**) **– $C_{16}H_{24}BNO_3$**:

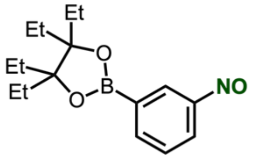

Purification was performed by CombiFlash column chromatography on silica gel using a gradient of 0-5% EtOAc in hexanes over 30 minutes. The product eluted at 1% EtOAc in hexanes, affording a red oil (101 mg, 70% yield) upon rotary evaporation; TLC: 5% EtOAc in hexanes, R_f = 0.6; visualized with UV. ^{1}H NMR (500 MHz, $CDCl_3$, 25 °C): δ 8.46 (t, J = 1.6 Hz, 1H), 8.14 (dt, J = 7.3, 1.2 Hz, 1H), 7.82 (dd, J = 7.9, 1.3 Hz, 1H), 7.58 (t, J = 7.6 Hz, 1H), 1.85 -1.74 (m, 8H), 0.99 (t, J = 7.5 Hz, 12H). ^{13}C NMR (125 MHz, $CDCl_3$, 25 °C): δ 165.8, 141.9, 128.7, 128.6, 128.4, 121.9, 89.6, 26.6, 8.9. HRMS (APCI) m/z: $[M]^{-}$• calcd for $C_{16}H_{24}BNO_3$, 289.1849; found 289.1862.

3-(4-(tert-butyl)phenyl)-2-oxa-3-azabicyclo[2.2.2]oct-5-ene (**7b**) **– $C_{16}H_{21}NO$**:

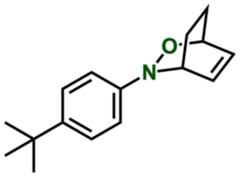

Purification was performed by CombiFlash column chromatography on silica gel using a gradient of 0-20% EtOAc in hexanes over 30 minutes. The product eluted at 8% EtOAc in hexanes, affording an off-white solid (113 mg, 93% yield) upon rotary evaporation; Melting point: 115.0 ºC-116.5 ºC. TLC: 20% EtOAc in hexanes, R_f = 0.3; visualized with UV. ^{1}H NMR (500 MHz, DMSO-d_6, 25 °C): δ 7.17 (d, J = 8.8 Hz, 2H), 6.87 (d, J = 8.7 Hz, 2H), 6.54 (ddd, J = 7.9, 5.9, 1.7 Hz, 1H), 6.16 (ddd, J = 8.2, 5.8, 1.5 Hz, 1H), 4.66 (ddd, J = 5.8, 3.9, 1.8 Hz, 1H), 4.62 – 4.43 (m, 1H), 2.14 – 1.89 (m, 2H), 1.60 – 1.40 (m, 1H), 1.30 – 1.26 (m, 1H), 1.22 (s, 9H). ^{13}C NMR (125 MHz, DMSO-d_6, 25 °C): δ 149.9, 143.1, 131.5, 130.5, 124.8, 116.3, 68.0, 54.7, 33.6, 31.2, 23.7, 20.8. HRMS (APCI) m/z: $[M–H]^-$ calcd for $C_{16}H_{20}NO$, 242.1550; found 242.1557. Analytical data are consistent with the literature.[5]

3-(3,4-difluorophenyl)-2-oxa-3-azabicyclo[2.2.2]oct-5-ene (**8b**) – **$C_{12}H_{11}F_2NO$**:

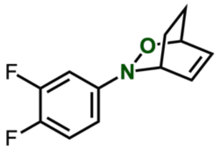

Purification was performed by CombiFlash column chromatography on silica gel using a gradient of 0-20% EtOAc in hexanes over 30 minutes. The product eluted at 9% EtOAc in hexanes, affording a yellow solid (86 mg, 77% yield) upon rotary evaporation; Melting point: 98.1 ºC - 98.6 ºC. TLC: 20% EtOAc in hexanes, $R_f = 0.2$; visualized with UV. ^{1}H NMR (500 MHz, DMSO-d_6, 25 °C): δ 7.2 (dt, J = 10.5, 9.0 Hz, 1H), 6.9 (ddd, J = 13.3, 7.3, 2.7 Hz, 1H), 6.8 (dddd, J = 9.1, 4.1, 2.7, 1.6 Hz, 1H), 6.6 (ddd, J = 7.9, 5.9, 1.7 Hz, 1H), 6.2 (ddd, J = 8.2, 5.8, 1.5 Hz, 1H), 4.7 (tt, J = 4.2, 1.5 Hz, 1H), 4.7 – 4.6 (m, 1H), 2.2 – 1.9 (m, 2H), 1.5 – 1.4 (m, 1H), 1.3 – 1.2 (m, 1H). ^{13}C NMR (125 MHz, DMSO-d_6, 25 °C): δ 149.9 (dd, $J_{C\text{-}F}$ = 6.9, 2.3 Hz), 149.2 (dd, $J_{C\text{-}F}$ = 243.1, 13.2 Hz), 144.1 (dd, $J_{C\text{-}F}$ = 238.7, 12.9 Hz), 131.6, 130.2, 116.9 (d, $J_{C\text{-}F}$ = 17.7 Hz), 112.6 (dd, $J_{C\text{-}F}$ = 5.9, 3.1 Hz, 1C), 105.8 (d, $J_{C\text{-}F}$ = 21.0 Hz), 68.6, 54.9, 23.5, 20.5. ^{19}F NMR (470 MHz, DMSO-d_6, 25 °C): δ -133.5 (dd, J = 23.0, 1.6 Hz, 1F), -143.8 (dd, J = 23.0, 1.6 Hz, 1F). HRMS (APCI) m/z: $[M+H]^+$ calcd for $C_{12}H_{12}ONF_2$, 224.0887; found 224.0880.

3-(4-fluoro-2-methylphenyl)-2-oxa-3-azabicyclo[2.2.2]oct-5-ene (**9b**) – **$C_{13}H_{14}FNO$**:

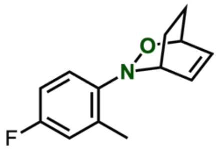

Purification was performed by CombiFlash column chromatography on silica gel using a gradient of 0-10% EtOAc in hexanes over 30 minutes. The product eluted at 0.5% EtOAc in hexanes, affording a yellow solid (90 mg, 82% yield) upon rotary evaporation; Melting point: 88.1 ºC-89.4 ºC. TLC: 10% EtOAc in hexanes, $R_f = 0.2$; visualized with UV. ^{1}H NMR (500 MHz, $CDCl_3$, 25 °C): δ 7.03 (dd, J = 8.9, 5.6 Hz, 1H), 6.80 – 6.77 (m, 1H), 6.75 – 6.71 (m, 2H), 6.05 (dd, J = 7.9, 1.5 Hz, 1H), 4.70 (dd, J = 5.8, 1.7 Hz, 1H), 3.98 – 3.76 (m, 1H), 2.32 (s, 3H), 2.29 – 2.24 (m, 2H), 1.54 – 1.49 (m, 1H), 1.43 – 1.39 (m, 1H). ^{13}C NMR (125 MHz, $CDCl_3$, 25 °C): δ 159.5 (d, $J_{C\text{-}F}$ = 241.5 Hz), 145.6 (d, $J_{C\text{-}F}$ = 2.5 Hz), 132.7, 131.05 (d, $J_{C\text{-}F}$ = 7.8 Hz), 129.2, 122.5 (d, $J_{C\text{-}F}$ = 8.3 Hz,), 116.8 (d, $J_{C\text{-}F}$ = 22.1 Hz), 112.1 (d, $J_{C\text{-}F}$ = 21.7 Hz), 69.4, 55.2, 23.9, 22.3, 18.1.

^{19}F NMR (470 MHz, $CDCl_3$, 25 °C): δ -120.74 (td, J = 8.8, 5.6 Hz, 1F). HRMS (APCI) m/z: $[M–H]^-$ calcd for $C_{13}H_{13}FNO$, 218.0987; found 218.0993.

2-(4-nitrosophenyl)ethan-1-ol **(10b) – $C_8H_9NO_2$**:

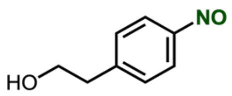

Purification was performed by CombiFlash column chromatography on silica gel using a gradient of 0-50% EtOAc in hexanes over 30 minutes. The product eluted at 18% EtOAc in hexanes, affording a brown oil (52 mg, 69% yield) upon rotary evaporation; TLC: 50% EtOAc in hexanes, R_f = 0.4; visualized with UV. ^{1}H NMR (500 MHz, $CDCl_3$, 25 °C): δ 7.86 (d, J = 8.3 Hz, 2H), 7.48 (d, J = 8.2 Hz, 2H), 3.95 (t, J = 6.4 Hz, 2H), 2.97 (t, J = 6.4 Hz, 2H). *Note, the OH hydrogen signal was not observed.* ^{13}C NMR (125 MHz, $CDCl_3$, 25 °C): δ 165.7, 147.8, 130.0, 121.5, 63.0, 39.4. HRMS (APCI) m/z: $[M]^{-\bullet}$ calcd for $C_8H_9NO_2$, 151.0633; found 151.0645.

2-methoxy-5-nitrosobenzaldehyde **(11b) – $C_8H_7NO_3$**:

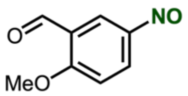

Purification was performed by CombiFlash column chromatography on silica gel using a gradient of 0-50% EtOAc in hexanes over 30 minutes. The product eluted at 17% EtOAc in hexanes, affording a green solid (62 mg, 75% yield) upon rotary evaporation; Melting point: 85.6 ºC-86.5 ºC. TLC: 50% EtOAc in hexanes, R_f = 0.5; visualized with UV. ^{1}H NMR (500 MHz, $CDCl_3$, 25 °C): δ 10.53 (s, 1H), 8.53 (d, J = 2.5 Hz, 1H), 8.01 (dd, J = 9.2, 2.5 Hz, 1H), 7.17 (d, J = 8.9 Hz, 1H), 4.10 (s, 3H). ^{13}C NMR (125 MHz, $CDCl_3$, 25 °C): δ 188.7, 166.3, 162.2, 127.2, 124.7, 123.9, 111.8, 56.9. HRMS (APCI) m/z: $[M–H]^-$ calcd for $C_8H_6NO_3$, 164.0353; found 164.0360.

7-nitroso-3,4-dihydronaphthalen-1(2H)-one **(12b) – $C_{10}H_9NO_2$**:

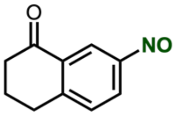

Purification was performed by CombiFlash column chromatography on silica gel using a gradient of 0-20% EtOAc in hexanes over 30 minutes. The product eluted at 8% EtOAc in hexanes, affording a yellow solid (65 mg, 74% yield) upon rotary evaporation; Melting point: 109.1 ºC-110.2 ºC. TLC: 20% EtOAc in hexanes, $R_f = 0.5$; visualized with UV. ^1H NMR (500 MHz, $CDCl_3$, 25 °C): δ 8.87 (d, J = 2.1 Hz, 1H), 7.64 (dd, J = 8.1, 2.1 Hz, 1H), 7.45 (d, J = 8.2 Hz, 1H), 3.05 (t, J = 6.1 Hz, 2H), 2.76 (dd, J = 7.3, 5.9 Hz, 2H), 2.27 – 1.92 (m, 2H). ^{13}C NMR (125 MHz, $CDCl_3$, 25 °C): δ 197.1, 164.9, 151.9, 133.7, 130.0, 123.4, 121.2, 39.0, 30.3, 22.7. HRMS (APCI) m/z: $[M–H]^-$ calcd for $C_{10}H_8NO_2$, 174.0561; found 174.0567.

4-nitrosophenyl acetate **(13b) – $C_8H_7NO_3$**:

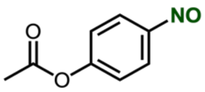

Purification was performed by CombiFlash column chromatography on silica gel using a gradient of 0-20% EtOAc in hexanes over 30 minutes. The product eluted at 5% EtOAc in hexanes, affording a black solid (60 mg, 73% yield) upon rotary evaporation; Melting point: 57.8 ºC-59.8 ºC. TLC: 20% EtOAc in hexanes, $R_f = 0.4$; visualized with UV. ^1H NMR (500 MHz, $CDCl_3$, 25 °C): δ 7.95 (d, J = 8.2 Hz, 2H), 7.36 (d, J = 8.7 Hz, 2H), 2.36 (s, 3H). ^{13}C NMR (125 MHz, $CDCl_3$, 25 °C): δ 168.5, 163.9, 156.1, 122.8, 122.6, 21.3. HRMS (APCI) m/z: $[M]^{-\bullet}$ calcd for $C_8H_7NO_3$, 165.0426; found 165.0415.

N-(4-nitrosophenyl)acetamide (**14b**) – **$C_8H_8N_2O_2$**:

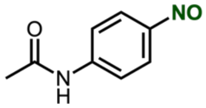

Purification was performed by CombiFlash column chromatography on silica gel using a gradient of 0-50% EtOAc in hexanes over 30 minutes. The product eluted at 25% EtOAc in hexanes, affording a green solid (67 mg, 82% yield) upon rotary evaporation; Melting point: 179.5 ºC-181.8 ºC. TLC: 50% EtOAc in hexanes, $R_f = 0.5$; visualized with UV. ^{1}H NMR (500 MHz, DMSO-d_6, 25 °C): δ 10.60 (s, 1H), 8.01 – 7.74 (m, 4H), 2.13 (s, 3H). ^{13}C NMR (125 MHz, DMSO-d_6, 25 °C): δ 169.6, 164.1, 146.8, 123.2, 118.2, 24.4. HRMS (APCI) m/z: $[M–H]^-$ calcd for $C_8H_7N_2O_2$, 163.0513; found 163.0520.

2-chloro-1-((3-fluorobenzyl)oxy)-4-nitrosobenzene (**15b**) – **$C_{13}H_9ClFNO_2$**:

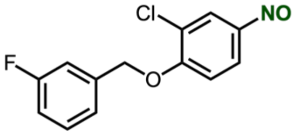

Purification was performed by CombiFlash column chromatography on silica gel using a gradient of 0-20% EtOAc in hexanes over 30 minutes. The product eluted at 4% EtOAc in hexanes, affording a blue solid (89 mg, 67% yield) upon rotary evaporation; Melting point: 90.4 ºC-91.1 ºC. TLC: 20% EtOAc in hexanes, $R_f = 0.3$; visualized with UV. ^{1}H NMR (500 MHz, $CDCl_3$, 25 °C): δ 8.33 – 8.15 (m, 1H), 7.62 (d, J = 2.4 Hz, 1H), 7.40 (td, J = 8.0, 5.8 Hz, 1H), 7.25 – 7.23 (m, 1H), 7.20 (dt, J = 9.4, 2.0 Hz, 1H), 7.18 (d, J = 8.7 Hz, 1H), 7.06 (td, J = 8.5, 2.6 Hz, 1H), 5.30 (s, 2H). ^{13}C NMR (125 MHz, $CDCl_3$, 25 °C): δ 163.2 (d, J = 246.5 Hz), 162.4, 159.6, 137.7 (d, J = 7.5 Hz, 1C), 130.6 (d, J = 8.3 Hz), 126.69, 125.04, 122.5 (d, J = 3.0 Hz), 120.1, 115.6 (d, J = 21.1 Hz), 114.1 (d, J = 22.5 Hz), 112.6, 70.6 (d, J = 2.1 Hz,). ^{19}F NMR (470 MHz, $CDCl_3$, 25 °C): δ -111.96 (td, J = 8.9, 5.7 Hz, 1F). HRMS (APCI) m/z: $[M–H]^-$ calcd for $C_{13}H_8ClFNO_2$, 264.0233; found 264.0240.

6-nitrosobenzo[d]thiazole **(16b) – $C_7H_4N_2OS$**:

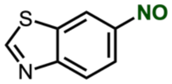

Purification was performed by CombiFlash column chromatography on silica gel using a gradient of 0-50% EtOAc in hexanes over 30 minutes. The product eluted at 12% EtOAc in hexanes, affording a yellow solid (55 mg, 67% yield) upon rotary evaporation; Melting point: 112.7 ºC-114.0 ºC. TLC: 50% EtOAc in hexanes, $R_f = 0.5$; visualized with UV. ^{1}H NMR (500 MHz, $CDCl_3$, 25 °C): δ 9.32 (s, 1H), 8.88 (d, J = 1.9 Hz, 1H), 8.26 (d, J = 8.7 Hz, 1H), 7.80 (dd, J = 8.7, 1.9 Hz, 1H). ^{13}C NMR (125 MHz, $CDCl_3$, 25 °C): δ 163.3, 160.4, 156.9, 134.9, 124.4, 120.7, 116.5. HRMS (APCI) m/z: $[M]^{-\bullet}$ calcd for $C_7H_4N_2OS$, 164.0044; found 164.0056.

methyl 1-methyl-4-nitroso-1H-pyrrole-2-carboxylate **(17b) – $C_7H_8N_2O_3$**:

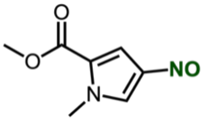

Purification was performed by CombiFlash column chromatography on silica gel using a gradient of 0-50% EtOAc in hexanes over 30 minutes. The product eluted at 19% EtOAc in hexanes, affording a blue solid (68 mg, 81% yield) upon rotary evaporation; Melting point: 113.6 ºC-115.8 ºC. TLC: 50% EtOAc in hexanes, $R_f = 0.5$; visualized with UV. ^{1}H NMR (400 MHz, DMSO-d_6, 80 °C): δ 8.78 (s, 1H), 6.60 (s, 1H), 4.03 (s, 3H), 3.81 (s, 3H). ^{13}C NMR (125 MHz, DMSO-d_6, 25 °C): δ 163.6, 160.9, 124.4, 51.8, 37.6. *Note that the dynamic nature of this molecule made detection of all carbons difficult.* However, these carbons could be detected by HSQC. Variable-temperature ^{1}H NMR spectra corroborate the dynamic nature, likely via reversible dimerization. HRMS (APCI) m/z: $[M+H]^+$ calcd for $C_7H_9N_2O_3$, 169.0613; found 169.0623.

4-nitrosophenyl N2,N6-bis(tert-butoxycarbonyl)-L-lysinate (**18b**) – **$C_{22}H_{33}N_3O_7$**:

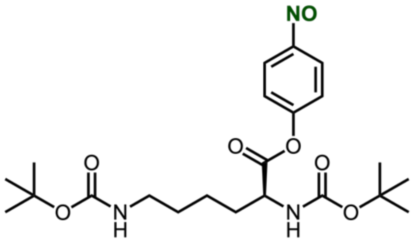

Purification was performed by CombiFlash column chromatography on silica gel using a gradient of 0-50% EtOAc in hexanes over 30 minutes. The product eluted at 16% EtOAc in hexanes, affording a brown oil (160 mg, 71% yield) upon rotary evaporation; TLC: 50% EtOAc in hexanes, $R_f = 0.4$; visualized with UV. ^1H NMR (500 MHz, $CDCl_3$, 25 °C): δ 7.95 (d, J = 8.5 Hz, 2H), 7.37 (d, J = 8.8 Hz, 2H), 5.23 (s, 1H), 4.53 - 4.49 (m, 2H), 3.16 (s, 2H), 1.99 (t, J = 7.0 Hz, 1H), 1.93 – 1.76 (m, 1H), 1.64 – 1.55 (m, 2H), 1.52 (m, 2H), 1.47 (s, 9H), 1.45 (s, 9H). ^{13}C NMR (125 MHz, $CDCl_3$, 25 °C): δ 170.9, 163.9, 156.4, 156.0 , 155.8, 122.8, 122.4, 80.5, 79.5, 54.0, 39.8, 31.6, 29.9, 28.6, 28.4, 22.6. HRMS (APCI) m/z: $[M–H]^-$ calcd for $C_{22}H_{32}N_3O_7$, 450.2246; found 450.2258.

tert-butyl 5-nitroso-1H-indole-1-carboxylate (**19b**) – **$C_{13}H_{14}N_2O_3$**:

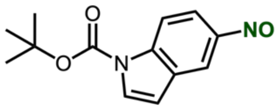

Purification was performed by CombiFlash column chromatography on silica gel using a gradient of 0-10% EtOAc in hexanes over 30 minutes. The product eluted at 2% EtOAc in hexanes, affording a green solid (45 mg, 37% yield) upon rotary evaporation; Melting point: 103.5 ºC-104.4 ºC. TLC: 10% EtOAc in hexanes, $R_f = 0.4$; visualized with UV. ^1H NMR (500 MHz, $CDCl_3$, 25 °C): δ 8.51 – 8.41 (m, 1H), 8.27 (d, J = 8.9 Hz, 1H), 7.73 (d, J = 3.7 Hz, 1H), 7.67 – 7.59 (m, 1H), 6.84 (dd, J = 3.8, 0.8 Hz, 1H), 1.70 (s, 9H). ^{13}C NMR (125 MHz, $CDCl_3$, 25 °C): δ 164.9, 149.2, 139.0, 130.6, 128.8, 119.7, 115.3, 115.2, 109.4, 85.3, 28.3. HRMS (APCI) m/z: $[M]^{-\bullet}$ calcd for $C_{13}H_{14}N_2O_3$, 246.1004; found 246.1015. Analytical data are consistent with the literature. [3]

4-nitrosobenzonitrile (**20b**) – **$C_7H_4N_2O$**:

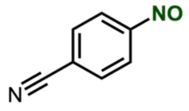

Purification was performed by CombiFlash column chromatography on silica gel using a gradient of 0-5% EtOAc in hexanes over 30 minutes. The product eluted at 1% EtOAc in hexanes, affording a yellow solid (51 mg, 77% yield) upon rotary evaporation; Melting point: 135.0 ºC-135.7 ºC. TLC: 5% EtOAc in hexanes, $R_f = 0.4$; visualized with UV. ^{1}H NMR (500 MHz, $CDCl_3$, 25 °C): δ 7.97 (apparent dd, J = 8.0, 1.3 Hz, 4H). ^{13}C NMR (125 MHz, $CDCl_3$, 25 °C): δ 162.3, 134.1, 120.9, 118.5, 117.6. HRMS (APCI) m/z: $[M]^{-}$• calcd for $C_7H_4N_2O$, 132.0324; found 132.0336. Analytical data are consistent with the literature. [3]

3-nitrosobenzaldehyde (**21b) – $C_7H_5NO_2$**:

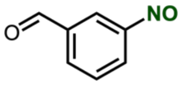

Purification was performed by CombiFlash column chromatography on silica gel using a gradient of 0-10% EtOAc in hexanes over 30 minutes. The product eluted at 4% EtOAc in hexanes, affording a yellow solid (45 mg, 67% yield) upon rotary evaporation; Melting point: 108.1 ºC-108.9 ºC. TLC: 10% EtOAc in hexanes, $R_f = 0.4$; visualized with UV. ^{1}H NMR (500 MHz, $CDCl_3$, 25 °C): δ 10.21 (s, 1H), 8.39 (d, J = 1.8 Hz, 1H), 8.27 (dt, J = 7.5, 1.4 Hz, 1H), 8.16 (dd, J = 7.9, 1.2 Hz, 1H), 7.84 (t, J = 7.7 Hz, 1H).^{13}C NMR (125 MHz, $CDCl_3$, 25 °C): δ 190.9, 164.8, 137.5, 135.0, 130.5, 125.8, 121.8. HRMS (APCI) m/z: $[M+H]^+$ calcd for $C_7H_6NO_2$, 136.0399; found 136.0412. Analytical data are consistent with the literature. [3]

2-chloro-5-nitroso-N-phenylbenzamide (**22b**) – **$C_{13}H_9ClN_2O_2$**:

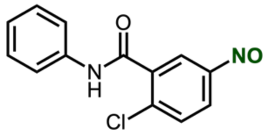

Purification was performed by CombiFlash column chromatography on silica gel using a gradient of 0-50% EtOAc in hexanes over 30 minutes. The product eluted at 11% EtOAc in hexanes, affording a green solid (81 mg, 62% yield) upon rotary evaporation; Melting point:

127.3 ºC-127.9 ºC. TLC: 50% EtOAc in hexanes, $R_f = 0.5$; visualized with UV. ^{1}H NMR (500 MHz, $CDCl_3$, 25 °C): δ 8.33 (d, J = 2.3 Hz, 1H), 7.86 (s, 1H), 7.84 (dd, J = 8.5, 2.3 Hz, 1H), 7.71 (d, J = 8.5 Hz, 1H), 7.67 – 7.61 (m, 2H), 7.41 (t, J = 8.0 Hz, 2H), 7.25 – 7.11 (m,1H). ^{13}C NMR (125 MHz, $CDCl_3$, 25 °C): δ 163.1, 162.6, 138.4, 137.2, 136.8, 131.9, 129.4, 125.6, 123.3, 122.1, 120.5. HRMS (APCI) m/z: $[M–H]^-$ calcd for $C_{13}H_8ClN_2O_2$, 259.0280; found 259.0287.

(1R,4S)-4-((4-bromophenyl)amino)cyclohex-2-en-1-ol **(23) – $C_{12}H_{14}BrNO$**:

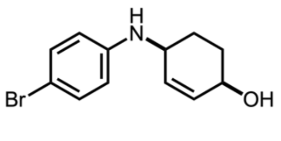

Purification was performed by CombiFlash column chromatography on silica gel using a gradient of 0-50% EtOAc in hexanes over 30 minutes. The product eluted at 31% EtOAc in hexanes, affording a yellow solid (86 mg, 64% yield) upon rotary evaporation; TLC: 50% EtOAc in hexanes, $R_f = 0.2$; visualized with UV. ^{1}H NMR (500 MHz, DMSO-d_6, 25 °C): δ 7.17 (d, J = 8.8 Hz, 2H), 6.56 (d, J = 8.9 Hz, 2H), 5.81 (d, J = 8.2 Hz, 1H), 5.78 – 5.75 (m, 1H), 5.67 - 5.64 (m, 1H), 4.77 (d, J = 4.5 Hz, 1H), 4.01 - 3.99 (m, 1H), 3.79 – 3.77 (m, 1H), 1.71 - 1.59 (m, 4H). ^{13}C NMR (125 MHz, DMSO-d_6, 25 °C): δ 147.1, 133.4, 131.4, 129.1, 114.3, 106.0, 63.5, 46.8, 28.6, 24.4. HRMS (ESI) m/z: $[M+H]^+$ calcd for $C_{12}H_{15}BrNO$, 268.0337; found 268.0325.

4-bromo-N-phenylaniline **(24) – $C_{12}H_{10}BrN$**:

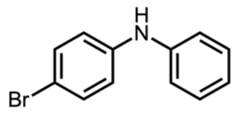

Purification was performed by CombiFlash column chromatography on silica gel using a gradient of 0-10% EtOAc in hexanes over 30 minutes. The product eluted at 1% EtOAc in hexanes, affording a pink solid (57 mg, 46% yield) upon rotary evaporation; TLC: 10% EtOAc in hexanes, $R_f = 0.3$; visualized with UV. ^{1}H NMR (500 MHz, DMSO-d_6, 25 °C): δ 8.28 (s, 1H), 7.35 (d, J = 8.8 Hz, 2H), 7.25 (dd, J = 8.5, 7.3 Hz, 2H), 7.15 – 7.04 (m, 2H), 7.00 (d, J = 8.8 Hz, 2H), 6.92 – 6.77 (m, 1H). ^{13}C NMR (125 MHz, DMSO-d_6, 25 °C): δ 143.0, 142.6,

131.8, 129.2, 120.4, 118.1, 117.4, 110.0. HRMS (ESI) m/z: $[M+H]^+$ calcd for $C_{12}H_{11}BrN$, 248.0075; found 248.0065.

(3S,6R)-2-(4-bromophenyl)-3,6-diphenyl-3,6-dihydro-2H-1,2-oxazine (**25**) – $\mathbf{C_{22}H_{18}BrNO}$:

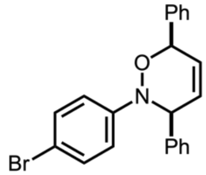

Purification was performed by CombiFlash column chromatography on silica gel using a gradient of 0-10% EtOAc in hexanes over 30 minutes. The product eluted at 2.5% EtOAc in hexanes, affording a brown solid (110 mg, 56% yield) upon rotary evaporation; TLC: 5 % EtOAc in hexanes, R_f = 0.3; visualized with UV. ^{1}H NMR (500 MHz, DMSO-d_6, 25 °C): δ 7.54 – 7.52 (m, 2H), 7.49 - 7.46 (m, 2H), 7.44 – 7.41 (m, 3H), 7.31 – 7.29 (m, 2H), 7.27 – 7.24 (m, 2H), 7.20 – 7.17 (m, 1H), 6.96 (d, J = 9.0 Hz, 2H), 6.23 -6.20 (m, 1H), 6.18 -6.15 (m, 1H), 5.67 (d, J = 1.9 Hz, 1H), 5.53 – 5.51 (m, 1H). ^{13}C NMR (125 MHz, DMSO-d_6, 25 °C): δ 147.5, 138.2, 138.1, 131.2, 128.8, 128.7, 128.7, 128.1, 127.9, 127.9, 127.5, 127.4, 117.3, 112.2, 78.8, 61.0. HRMS (ESI) m/z: $[M+H]^+$ calcd for $C_{22}H_{19}BrNO$, 392.0650; found 392.0636.

(E)-1-(4-bromophenyl)-2-phenyldiazene (**26**) – $\mathbf{C_{12}H_9BrN_2}$:

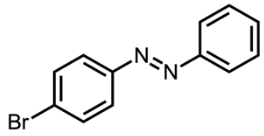

Purification was performed by CombiFlash column chromatography on silica gel using a gradient of 0-10% EtOAc in hexanes over 30 minutes. The product eluted at 0.4% EtOAc in hexanes, affording an orange solid (51 mg, 39% yield) upon rotary evaporation; TLC: 5 % EtOAc in hexanes, R_f = 0.5; visualized with UV. ^{1}H NMR (500 MHz, DMSO-d_6, 25 °C): δ 7.90 (dd, J = 7.8, 1.9 Hz, 2H), 7.86 – 7.77 (m, 4H), 7.68 – 7.54 (m, 3H). ^{13}C NMR (125 MHz, DMSO-d_6, 25 °C): δ 151.8, 150.8, 132.6, 131.9, 129.6, 125.0, 124.5, 122.7. HRMS (ESI) m/z: $[M+H]^+$ calcd for $C_{12}H_9BrN_2$, 261.0027; found 261.0018.

***bis(perfluorophenyl)(phenyl)phosphine oxide* (*S1*)**: $C_{18}H_5F_{10}OP$

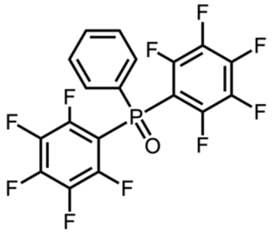

Isolated during the synthesis of compound **5b**. Purification was performed by CombiFlash column chromatography on silica gel using a gradient of 0-50% EtOAc in hexanes over 40 minutes. The product eluted at 21% EtOAc in hexanes, affording white solid (220 mg, 64% yield) upon rotary evaporation; TLC: 50% EtOAc in hexanes, R_f = 0.3; visualized with UV. ^{1}H NMR (500 MHz, DMSO-d_6, 25 °C): δ 7.87 (dd, J = 15.1, 7.7 Hz, 2H), 7.78 (t, J = 7.6 Hz, 1H), 7.65 (dt, J = 11.3, 5.3 Hz, 2H). *Note: because long range C-F, C-P, and P-F couplings can be hard to distinguish, only $^1J_{C\text{-}F}$ couplings are assigned. Other resolved coupling constants are reported but not assigned to F or P coupling.* ^{13}C NMR (125 MHz, DMSO-d_6, 25 °C): δ 146.69 (dm, $J_{C\text{-}F}$ = 252.3 Hz), 144.19 (dm, $J_{C\text{-}F}$ = 258.3 Hz), 137.56 (dm, $J_{C\text{-}F}$ = 244.5 Hz), 133.94, 130.58 (d, J = 12.3 Hz), 130.6 (d, J = 121 Hz) 129.24 (apparent dm, J=14 Hz), 106.97 (d, J = 97.6 Hz). ^{19}F NMR (470 MHz, DMSO-d_6, 25 °C): δ -131.7 (m, 4F), -144.9 (m, 2F), -159.3 (ddd, J = 43.5, 20.6, 10.3 Hz, 4F). ^{31}P NMR (202 MHz, DMSO-d_6, 25 °C): δ 7.21. HRMS (APCI) m/z: $[M+H]^+$ calcd for $C_{18}H_6F_{10}OP$, 458.9997; found 458.9975.

15. References

(1) Oka, N.; Yamada, T.; Sajiki, H.; Akai, S.; Ikawa, T. Aryl Boronic Esters Are Stable on Silica Gel and Reactive under Suzuki–Miyaura Coupling Conditions. *Org. Lett.* **2022**, *24* (19), 3510–3514. https://doi.org/10.1021/acs.orglett.2c01174.

(2) Aubineau, T.; Laurent, J.; Olanier, L.; Guérinot, A. Design, Characterization and Evaluation of a Lab-Made Photoreactor: A First Step Towards Standardized Procedures in Photocatalysis. *Chemistry–Methods* **2023**, *3* (11), e202300002. https://doi.org/10.1002/cmtd.202300002.

(3) Molander, G. A.; Cavalcanti, L. N. Nitrosation of Aryl and Heteroaryltrifluoroborates with Nitrosonium Tetrafluoroborate. *J. Org. Chem.* **2012**, *77* (9), 4402–4413. https://doi.org/10.1021/jo300551m.

(4) Prakash, G. K. S.; Gurung, L.; Schmid, P. C.; Wang, F.; Thomas, T. E.; Panja, C.; Mathew, T.; Olah, G. A. *Ipso*-Nitrosation of Arylboronic Acids with Chlorotrimethylsilane and Sodium Nitrite. *Tetrahedron Letters* **2014**, *55* (12), 1975–1978. https://doi.org/10.1016/j.tetlet.2014.01.138.

(5) Zhao, D.; Johansson, M.; Bäckvall, J.-E. In Situ Generation of Nitroso Compounds from Catalytic Hydrogen Peroxide Oxidation of Primary Aromatic Amines and Their One-Pot Use in Hetero-Diels–Alder Reactions. *European Journal of Organic Chemistry* **2007**, *2007* (26), 4431–4436. https://doi.org/10.1002/ejoc.200700368.

16. NMR Spectra

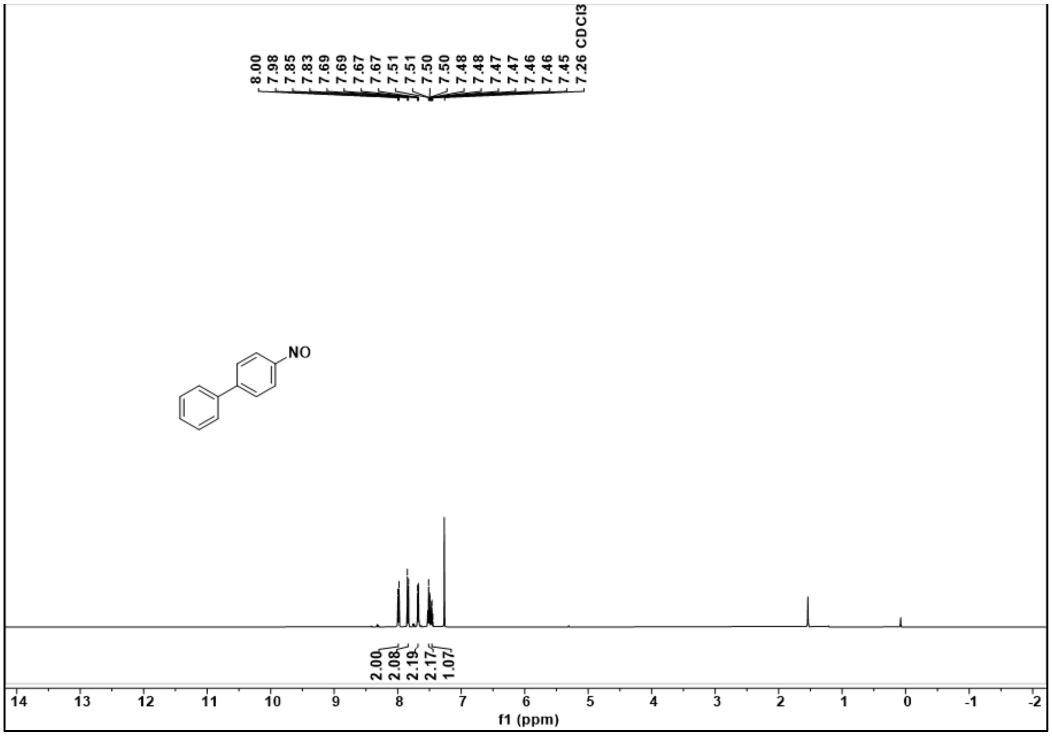

^{1}H NMR spectrum of **2b** ($CDCl_3$, 500 MHz)

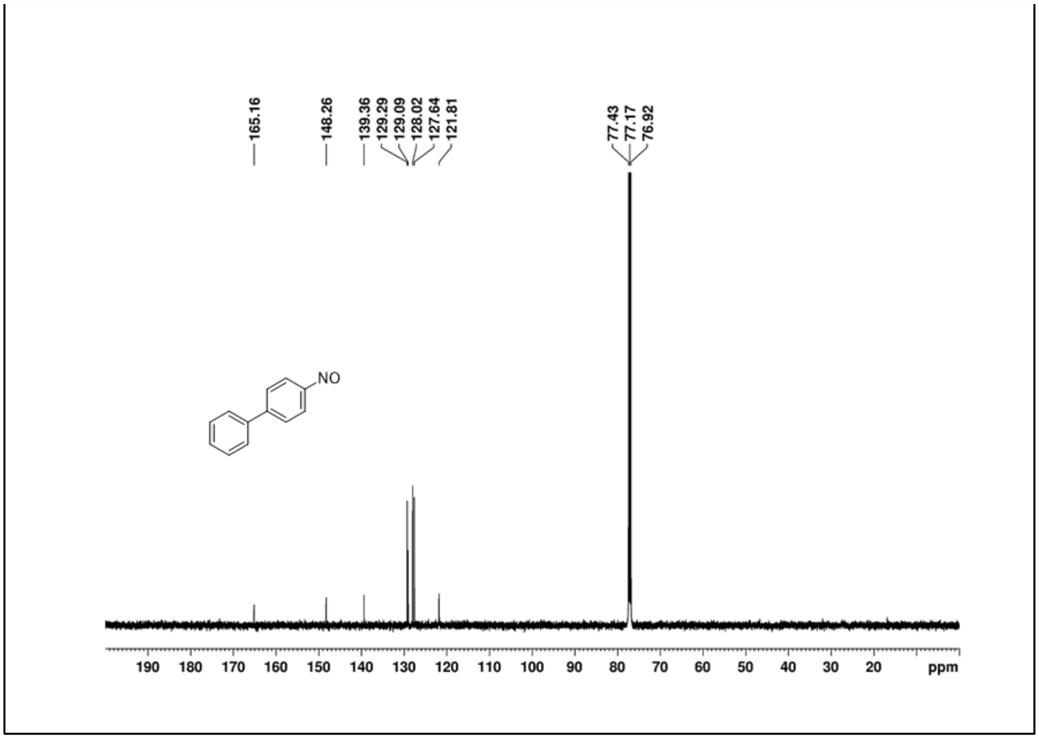

^{13}C NMR spectrum of **2b** ($CDCl_3$, 125 MHz)

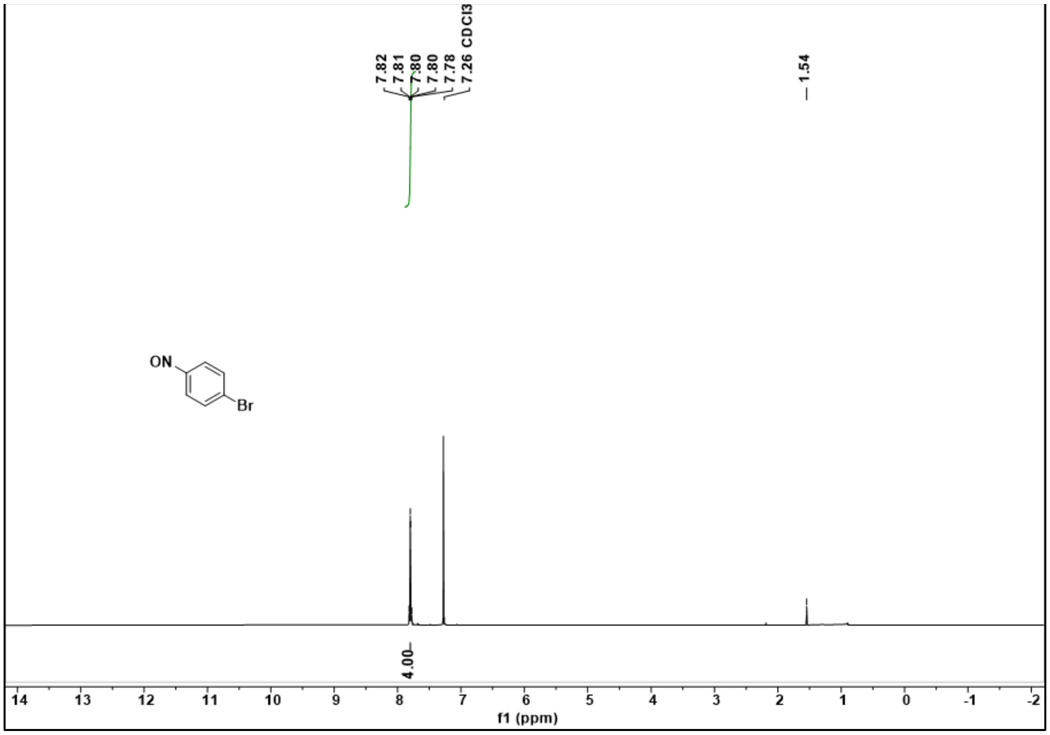

^{1}H NMR spectrum of **3b** ($CDCl_3$, 500 MHz)

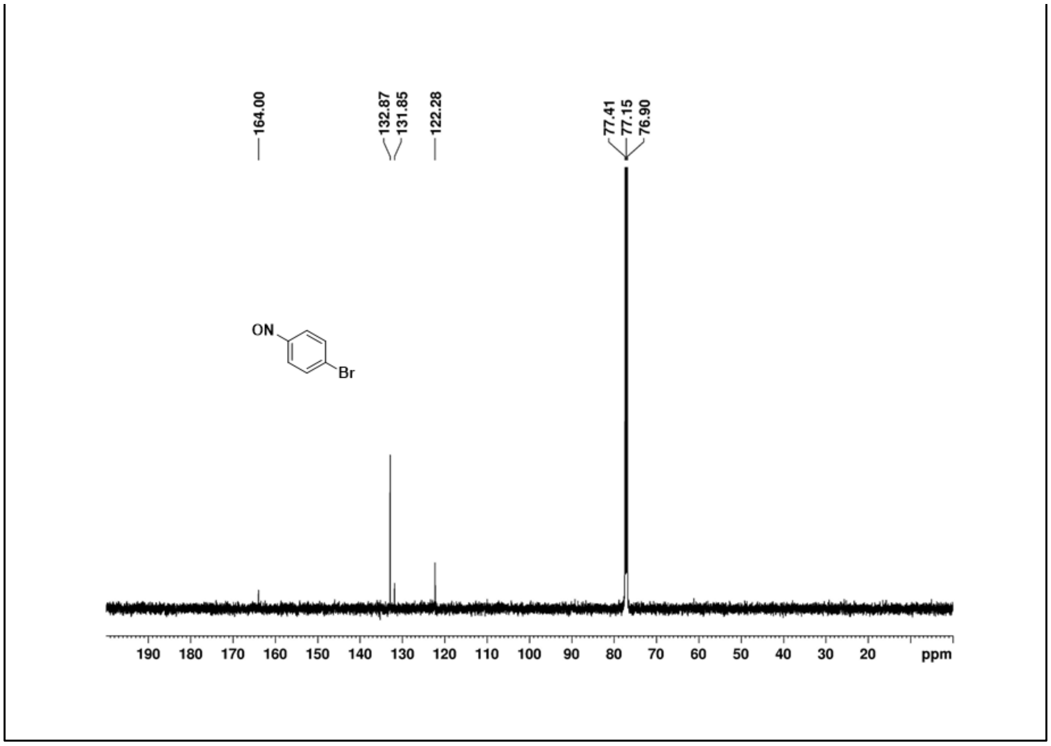

^{13}C NMR spectrum of **3b** ($CDCl_3$, 125 MHz)

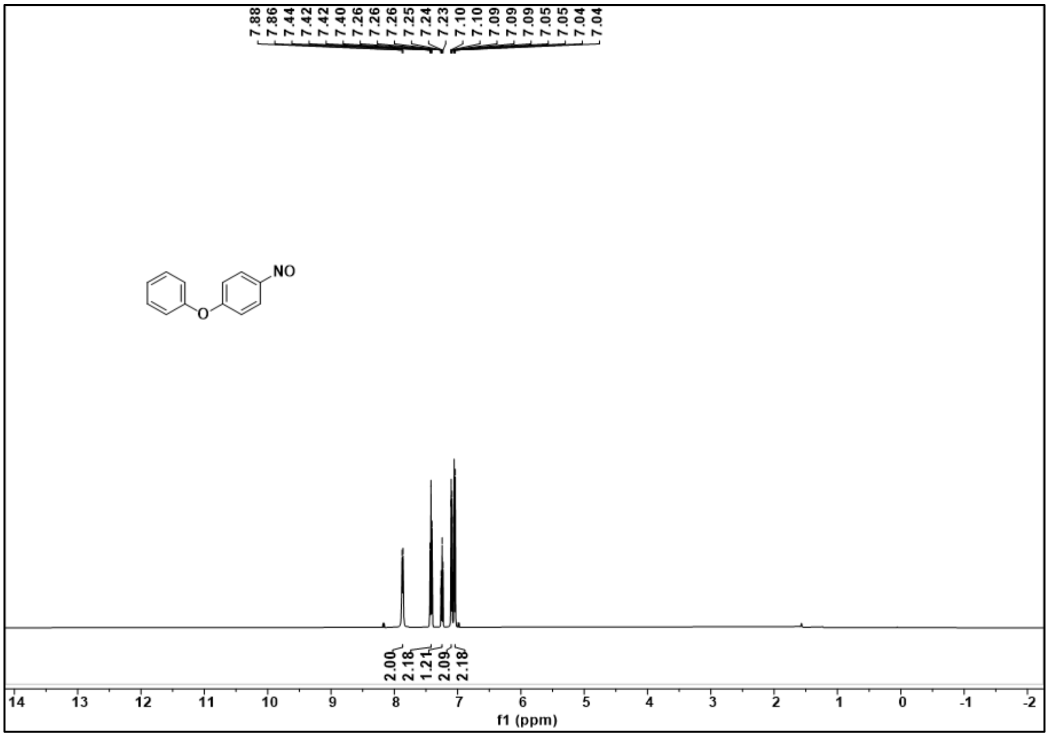

^{1}H NMR spectrum of **4b** ($CDCl_3$, 500 MHz)

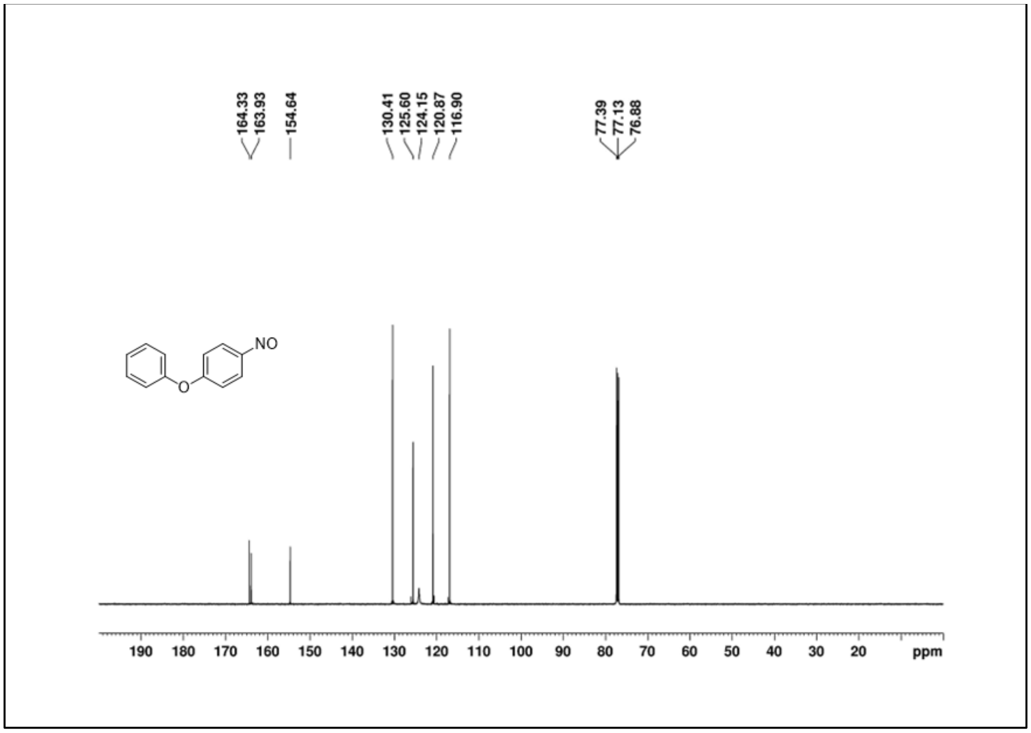

^{13}C NMR spectrum of **4b** ($CDCl_3$, 125 MHz)

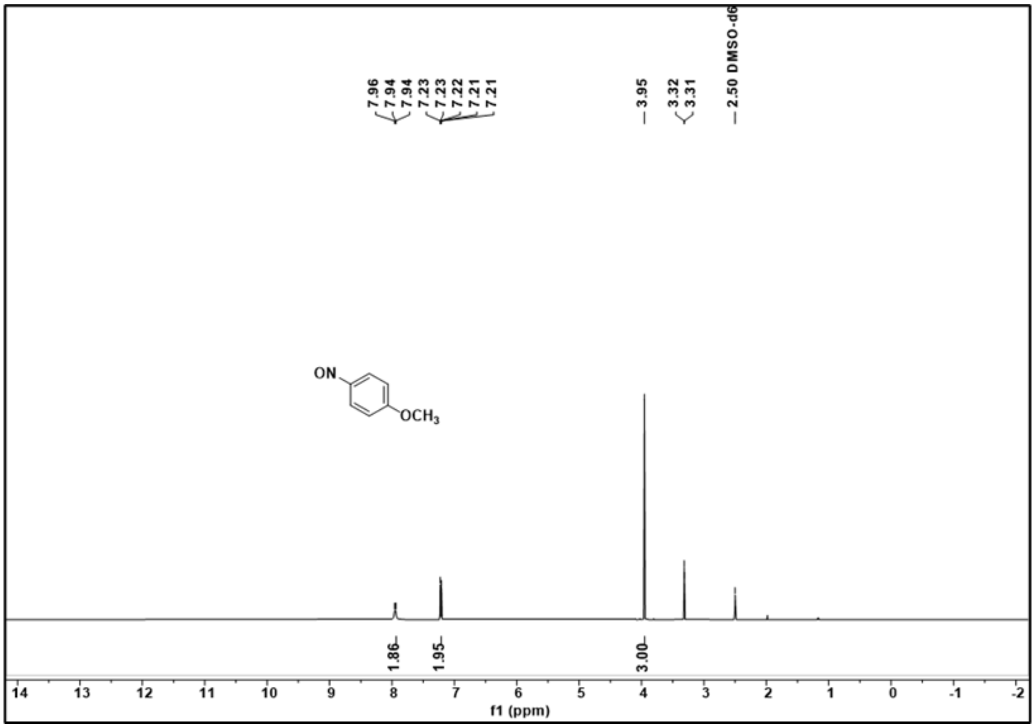

^{1}H NMR spectrum of **5b** (DMSO-d_6, 500 MHz)

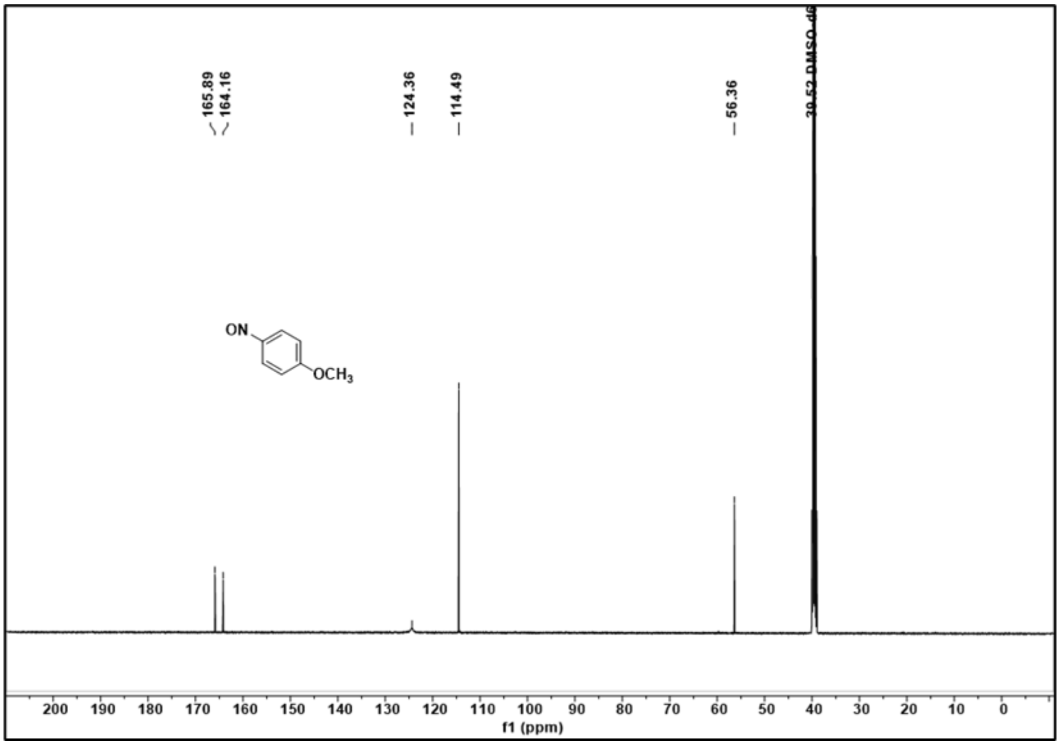

^{13}C NMR spectrum of **5b** (DMSO-d_6, 125 MHz)

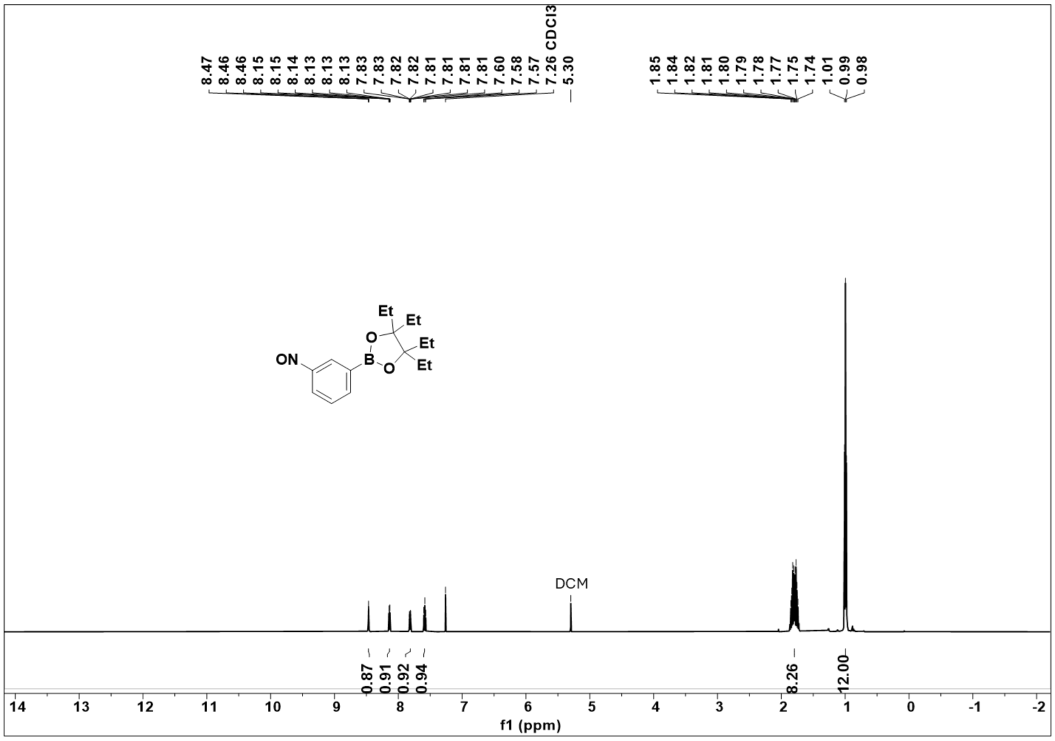

^{1}H NMR spectrum of **6b** ($CDCl_3$, 500 MHz)

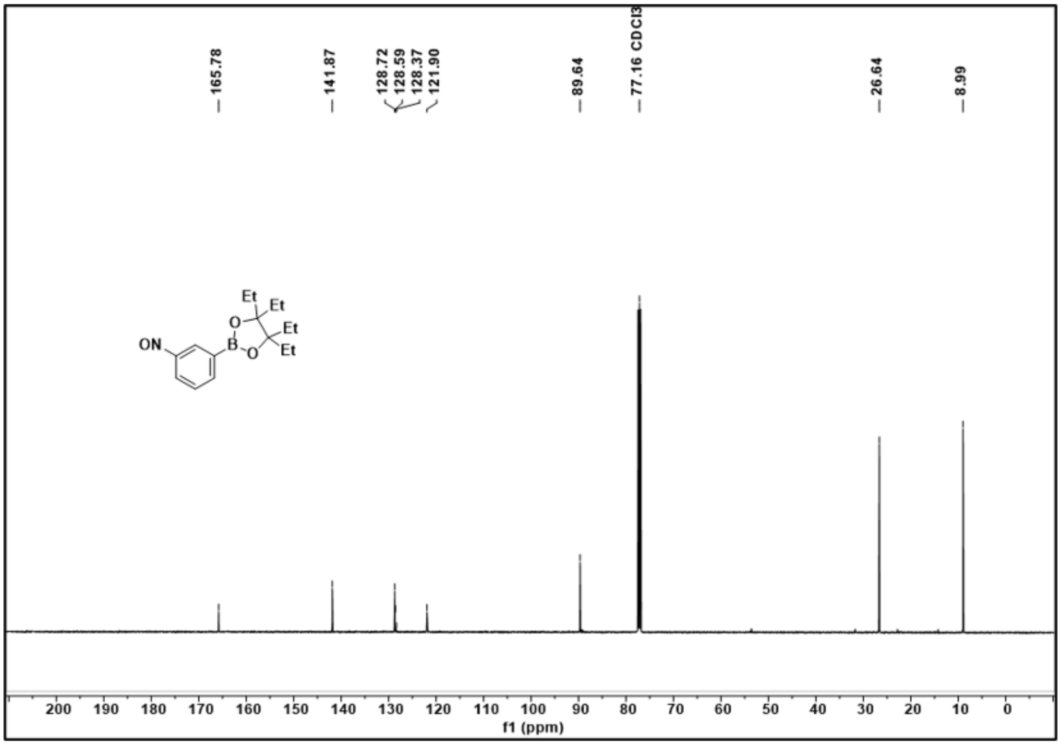

^{13}C NMR spectrum of **6b** ($CDCl_3$, 125 MHz)

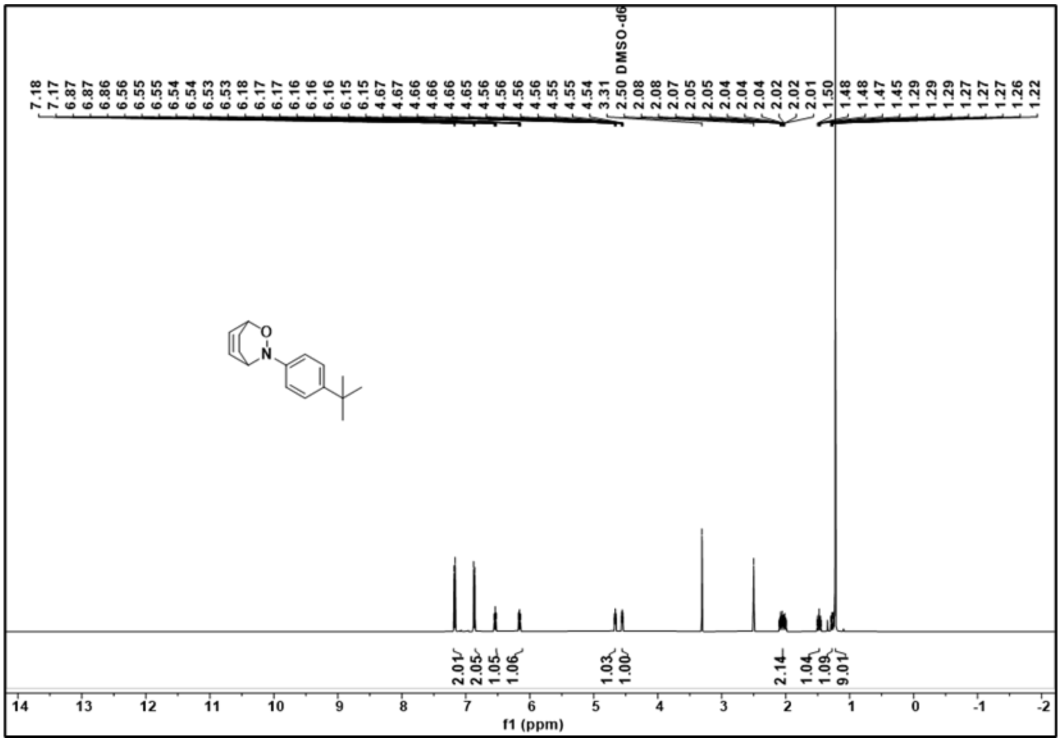

^{1}H NMR spectrum of **7b** (DMSO-d_6, 500 MHz)

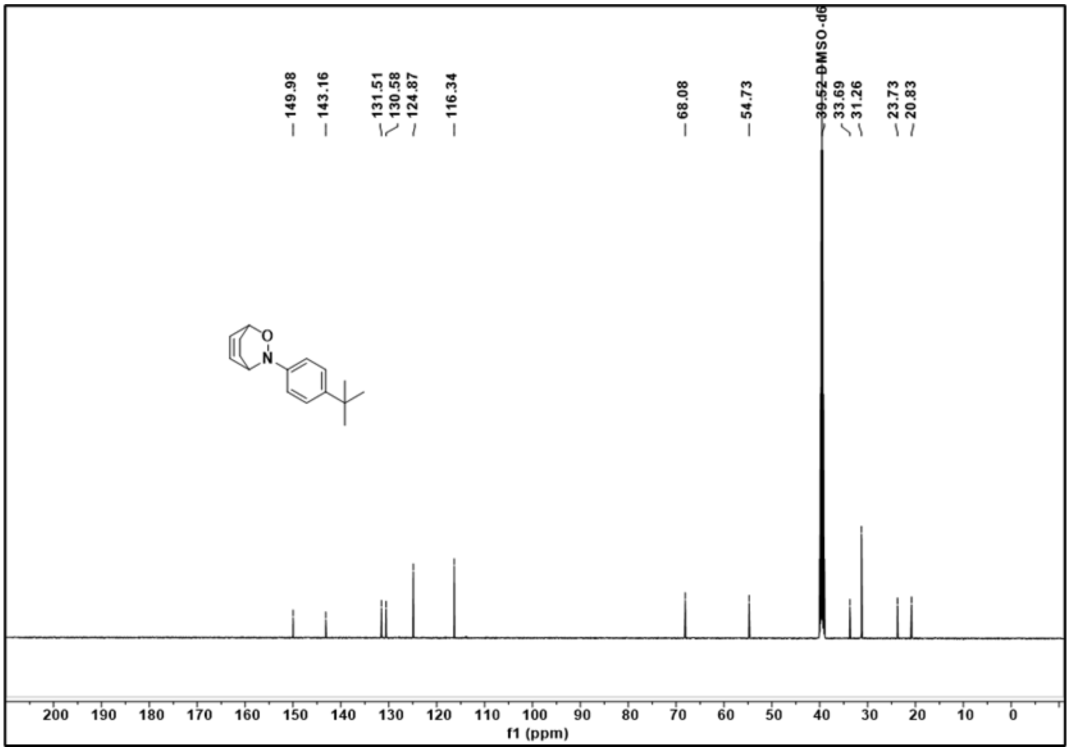

^{13}C NMR spectrum of **7b** (DMSO-d_6, 125 MHz)

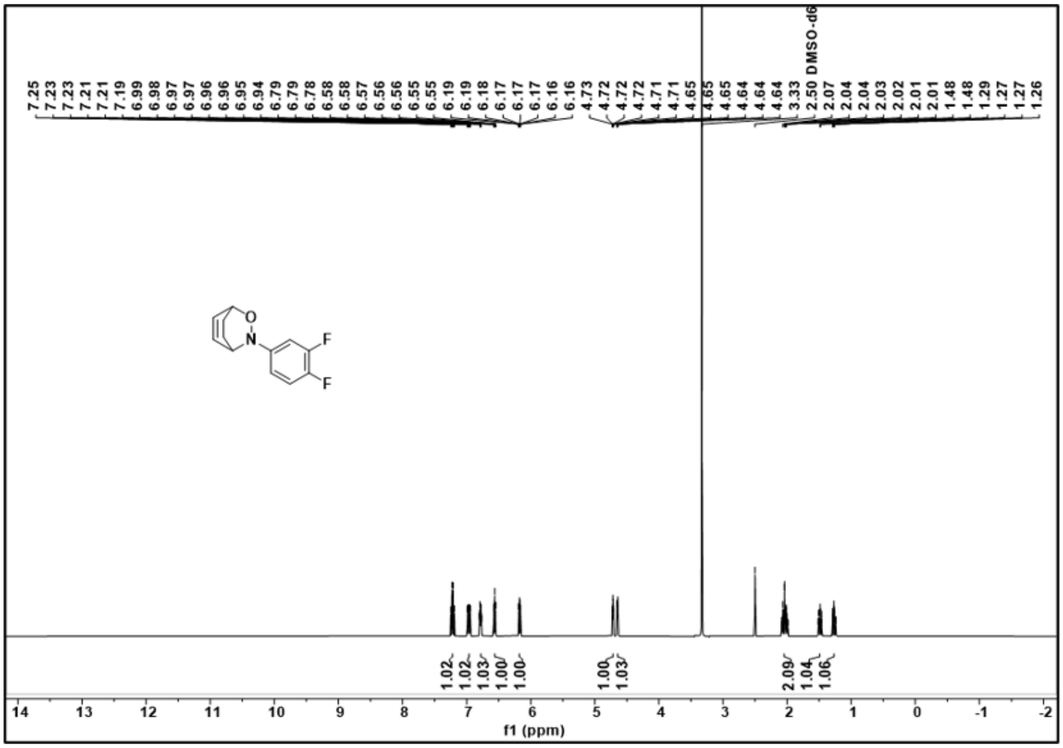

^{1}H NMR spectrum of **8b** (DMSO-d6, 500 MHz)

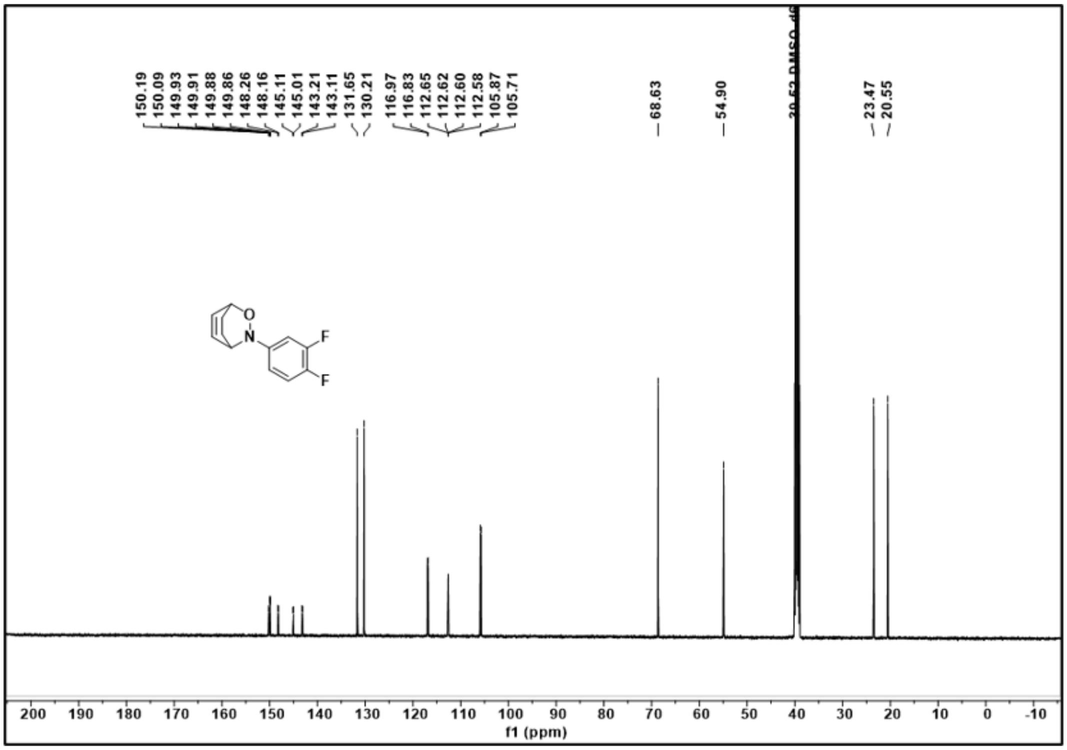

^{13}C NMR spectrum of **8b** (DMSO-d_6, 125 MHz)

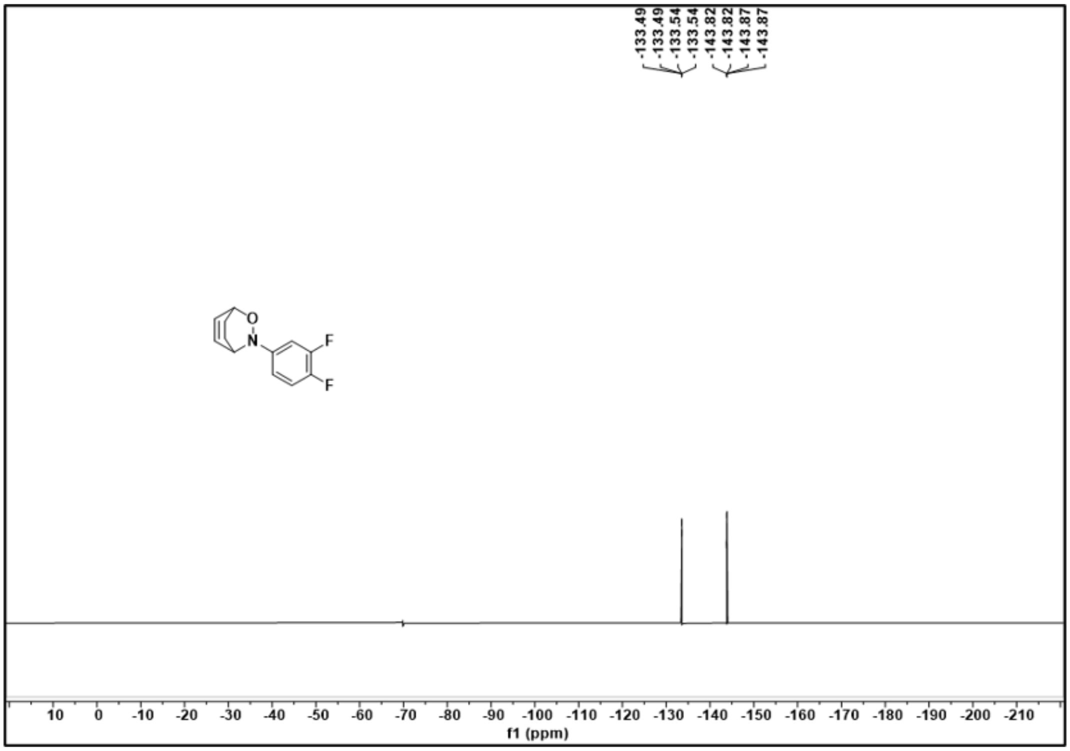

^{19}F NMR spectrum of **8b** (DMSO-d_6, 470 MHz)

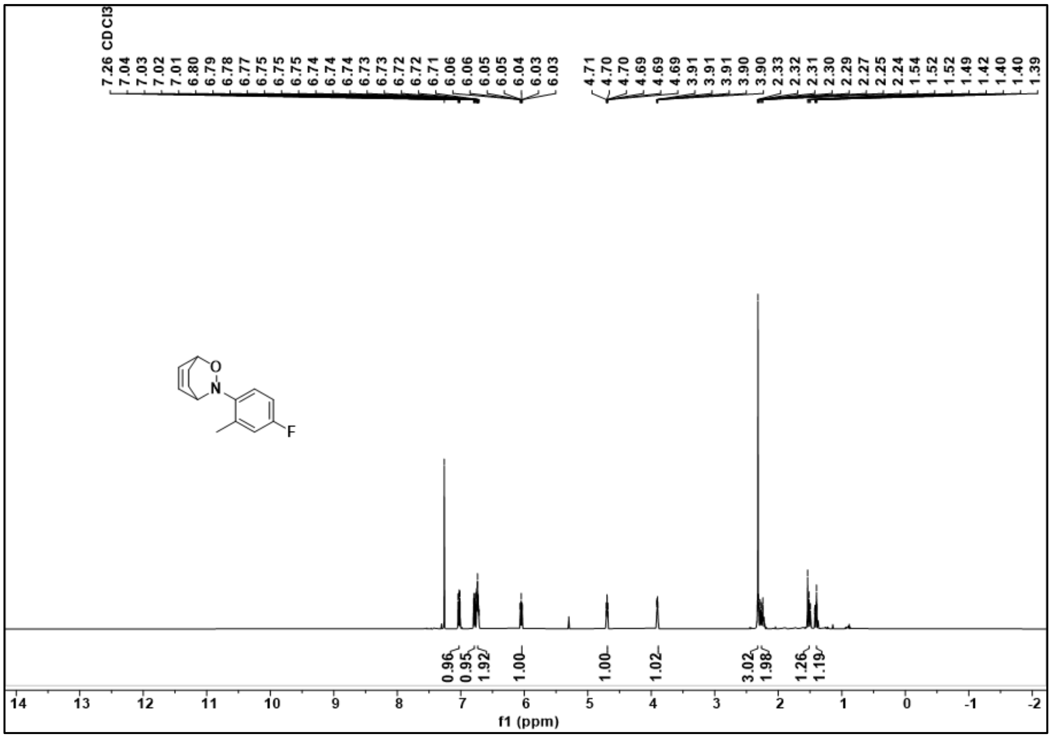

^{1}H NMR spectrum of **9b** ($CDCl_3$, 500 MHz)

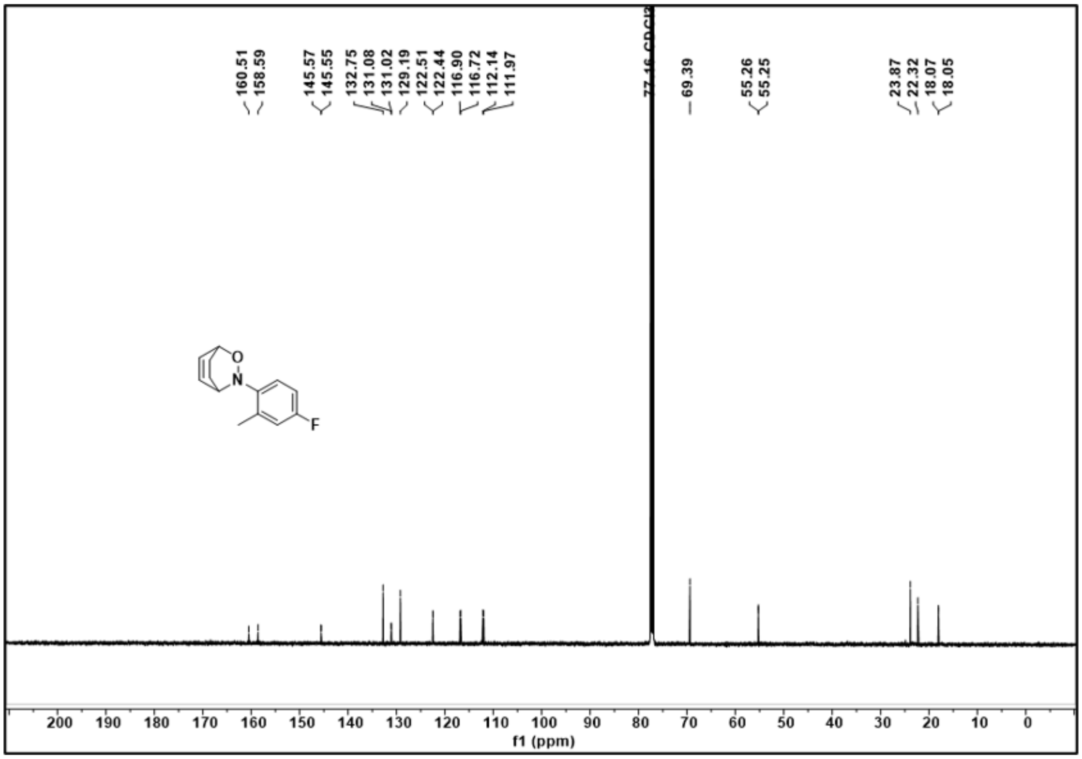

^{13}C NMR spectrum of **9b** (CDCl3, 125 MHz)

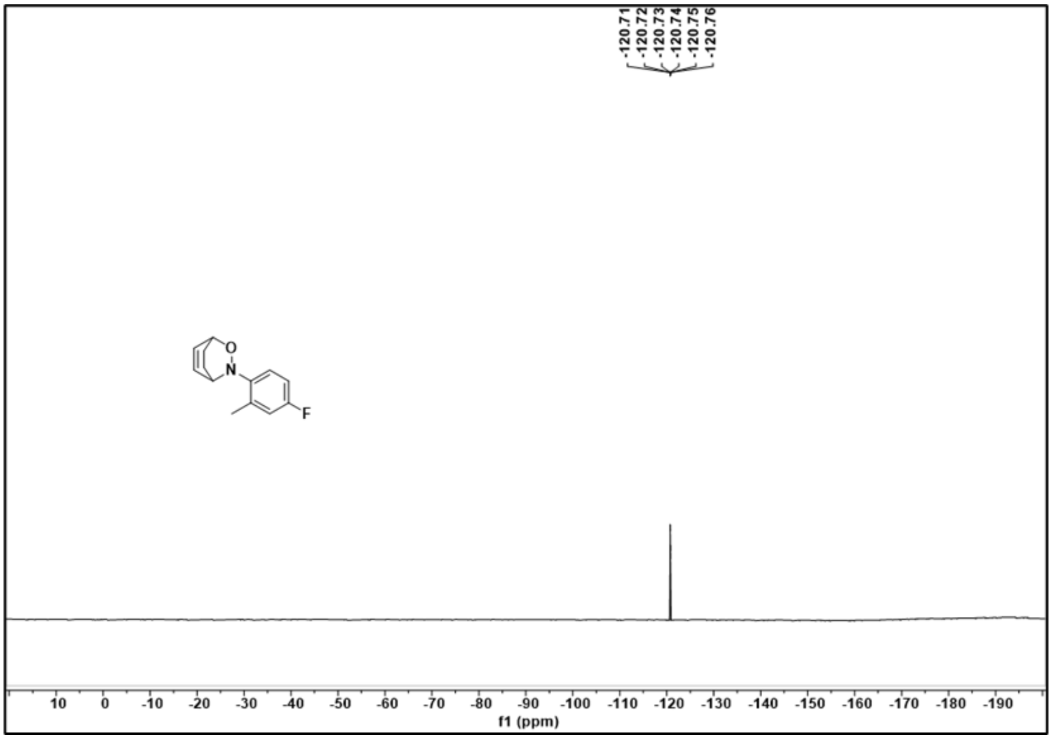

^{19}F NMR spectrum of **9b** (CDCl3, 470 MHz)

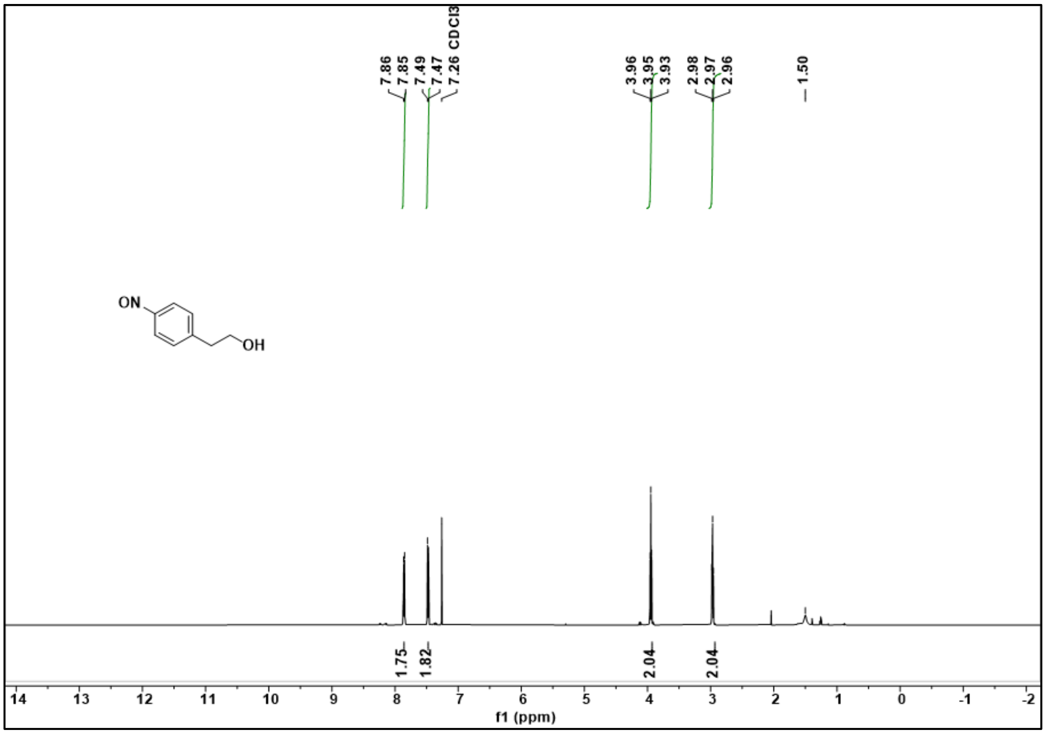

^{1}H NMR spectrum of **10b** ($CDCl_3$, 500 MHz)

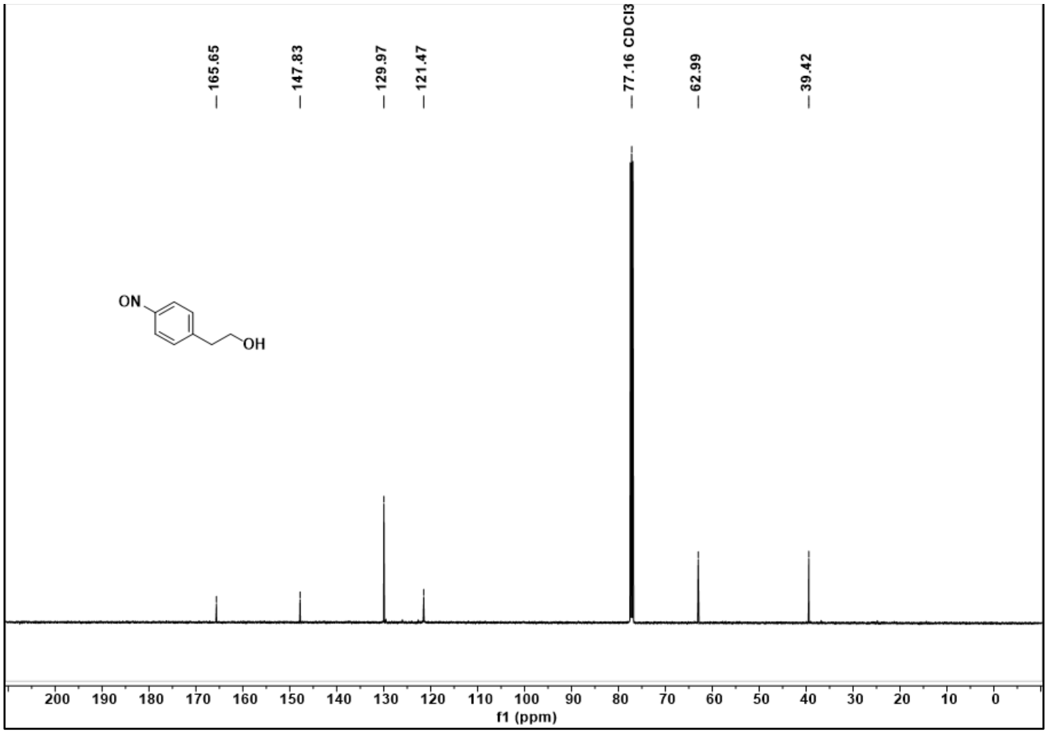

^{13}C NMR spectrum of **10b** ($CDCl_3$, 125 MHz)

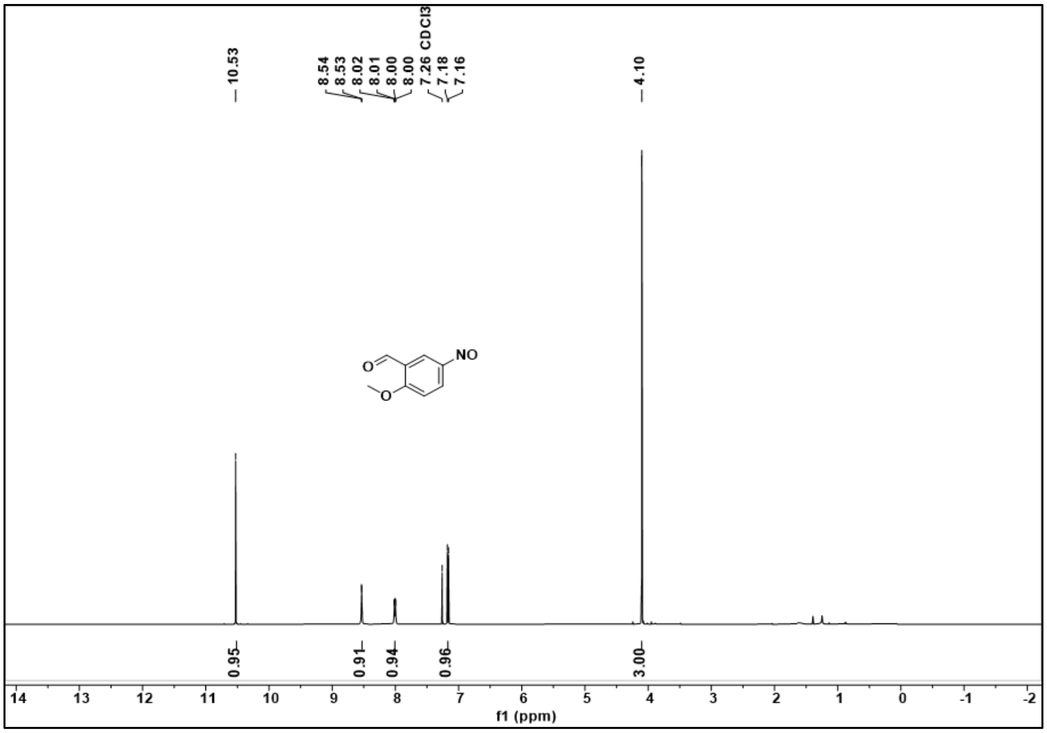

^{1}H NMR spectrum of **11b** ($CDCl_3$, 500 MHz)

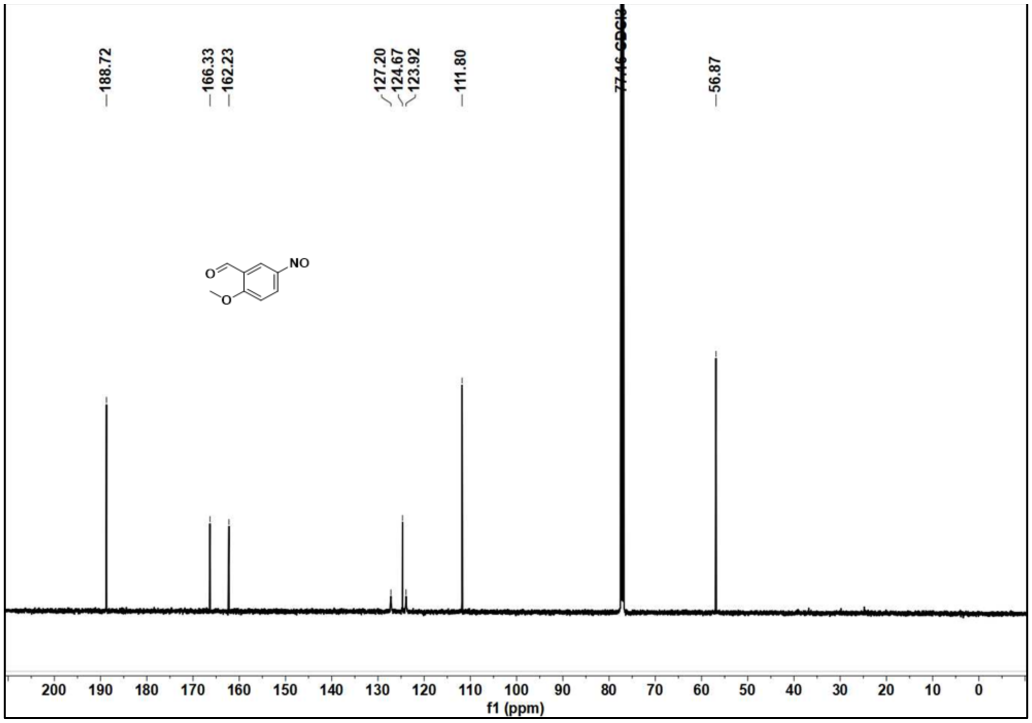

^{13}C NMR spectrum of **11b** ($CDCl_3$, 125 MHz)

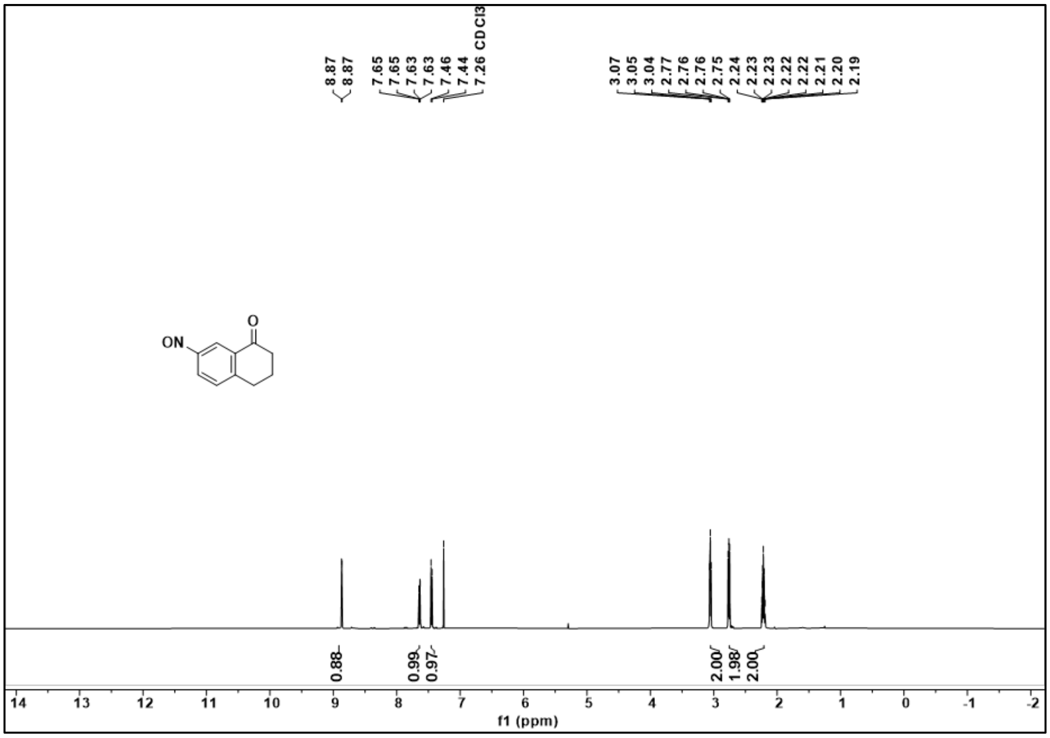

^{1}H NMR spectrum of **12b** ($CDCl_3$, 500 MHz)

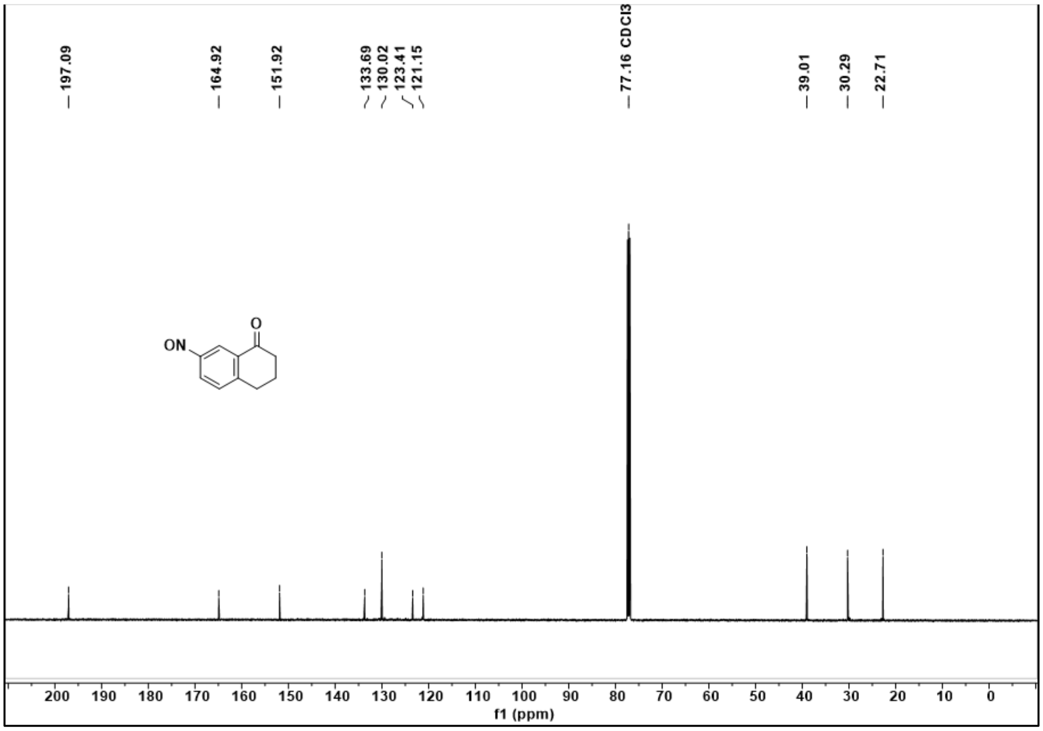

^{13}C NMR spectrum of **12b** ($CDCl_3$, 125 MHz)

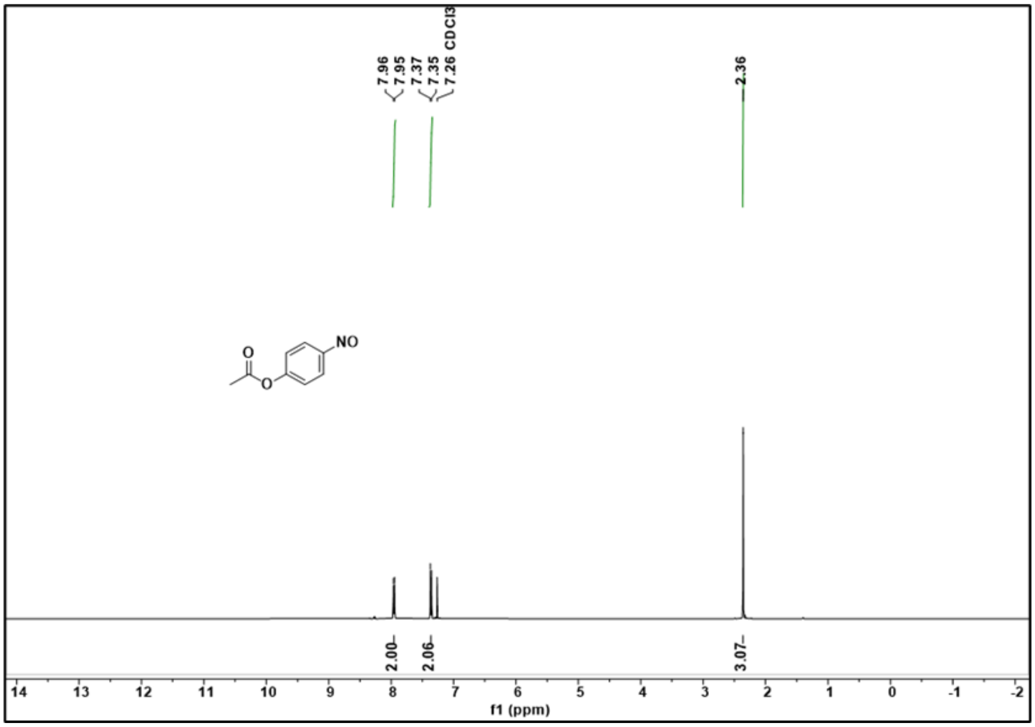

^{1}H NMR spectrum of **13b** ($CDCl_3$, 500 MHz)

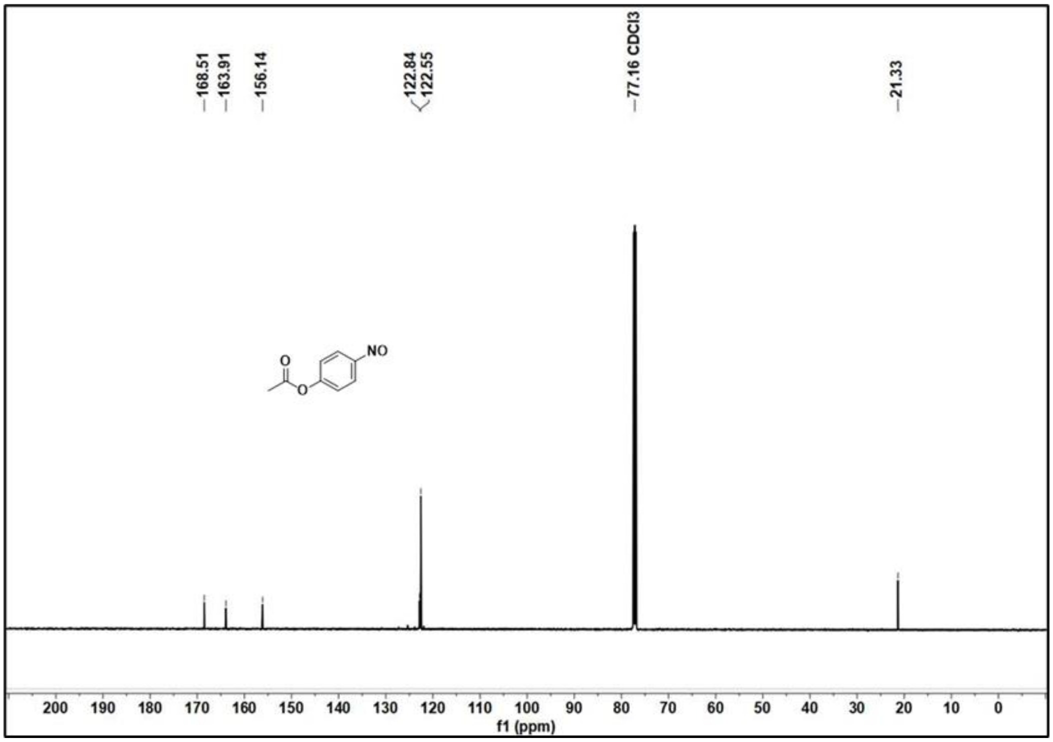

^{13}C NMR spectrum of **13b** ($CDCl_3$, 125 MHz)

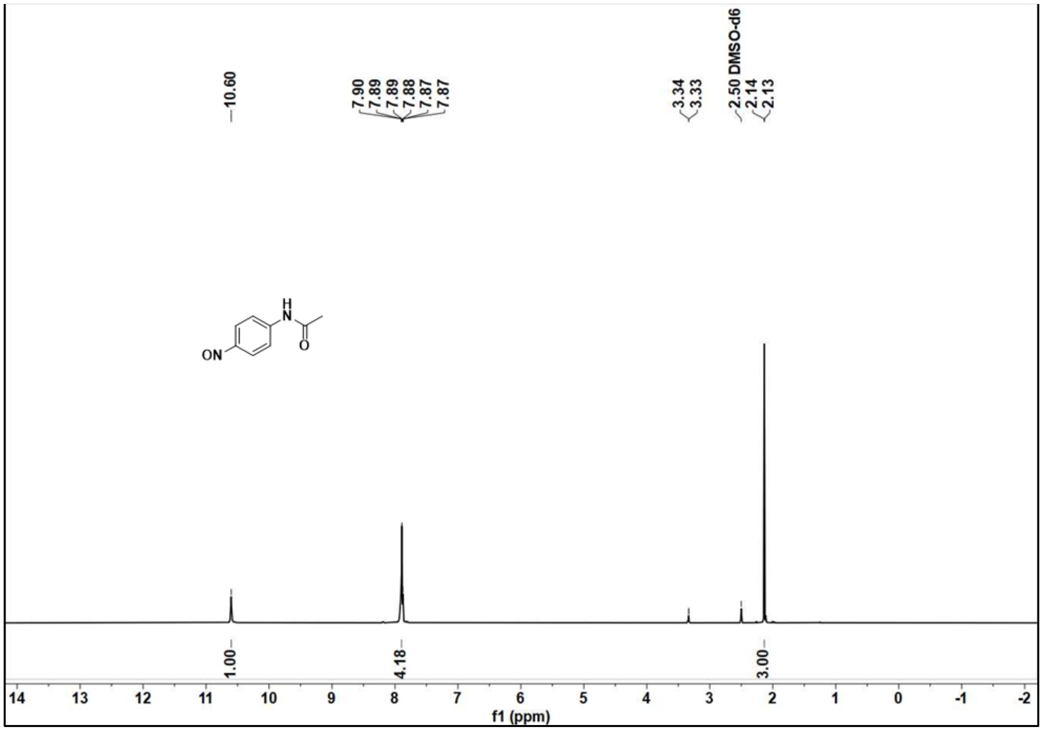

^{1}H NMR spectrum of **14b** (DMSO-d_6, 500 MHz)

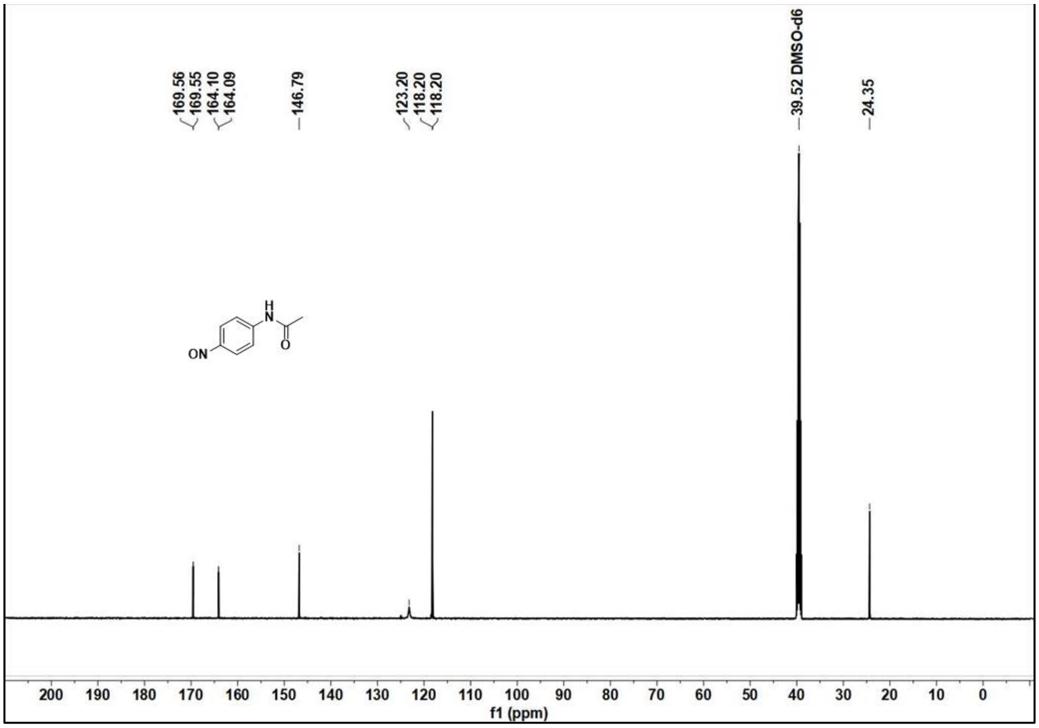

^{13}C NMR spectrum of **14b** (DMSO-d_6, 125 MHz)

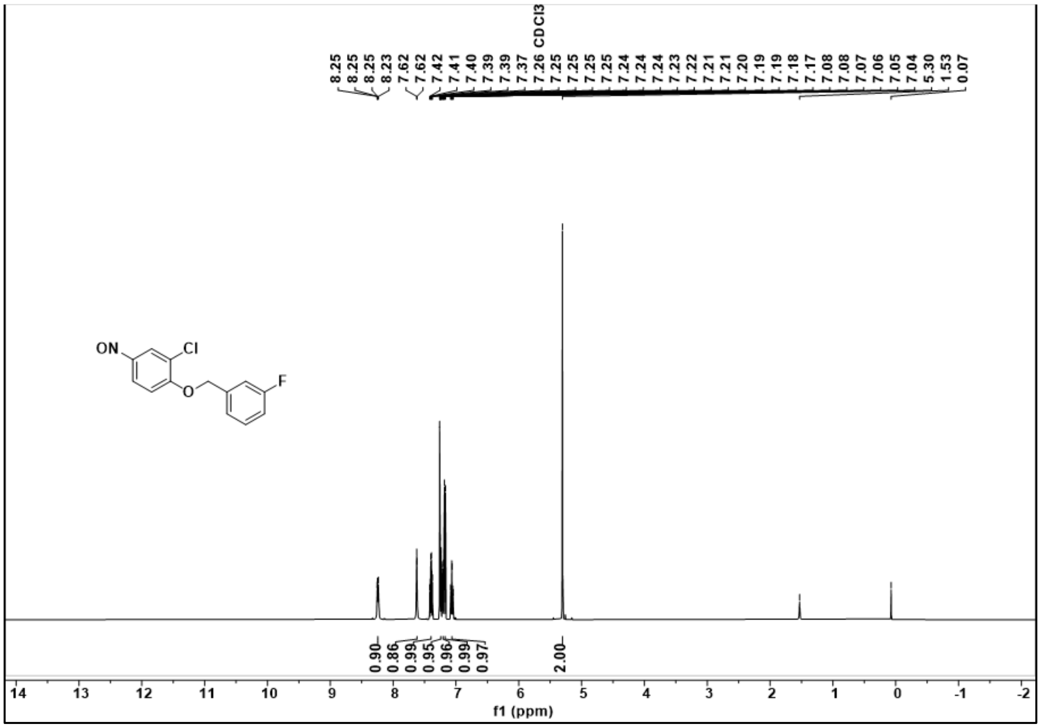

^{1}H NMR spectrum of **15b** ($CDCl_3$, 500 MHz)

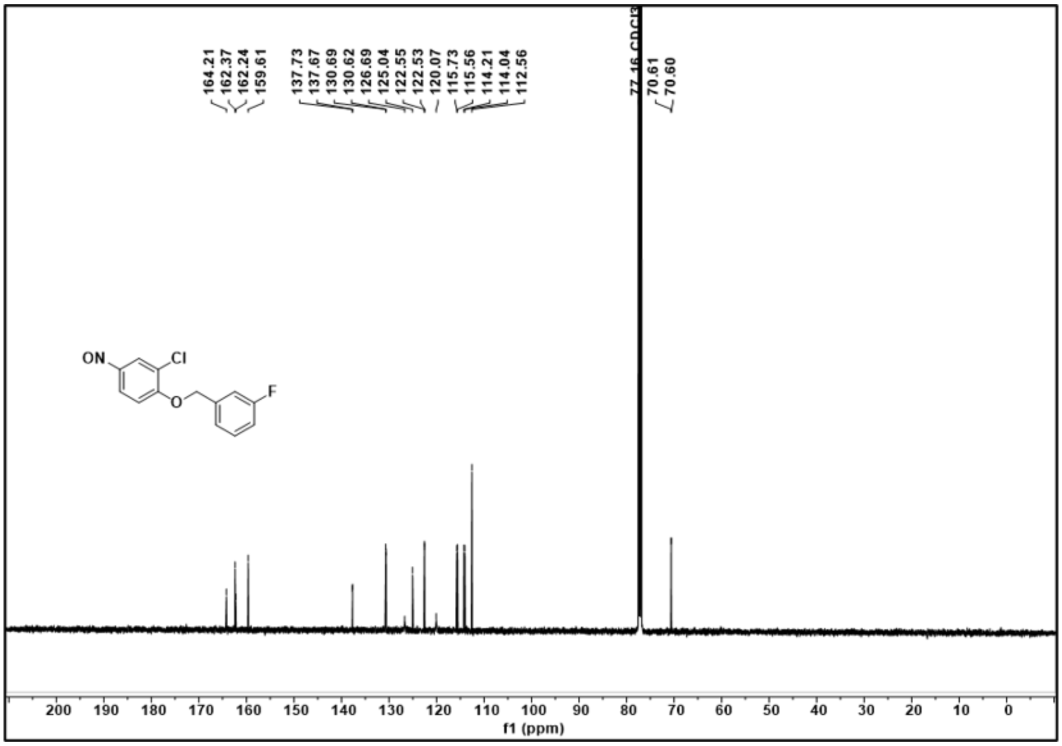

^{13}C NMR spectrum of **15b** ($CDCl_3$, 125 MHz)

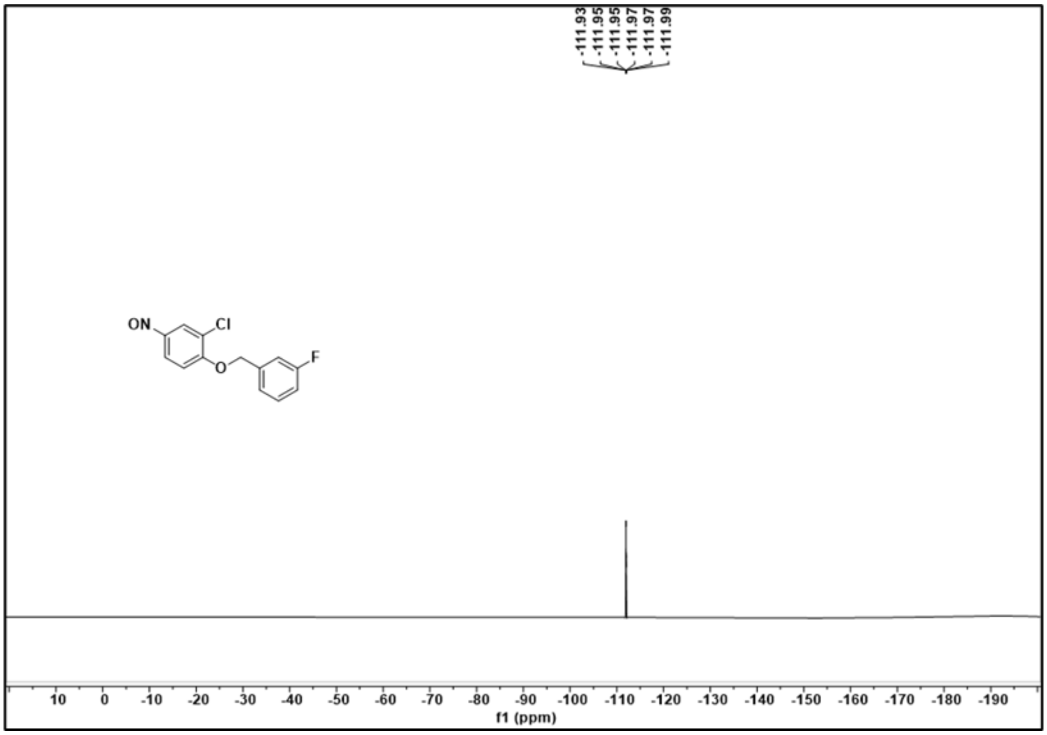

^{19}F NMR spectrum of **15b** ($CDCl_3$, 470 MHz)

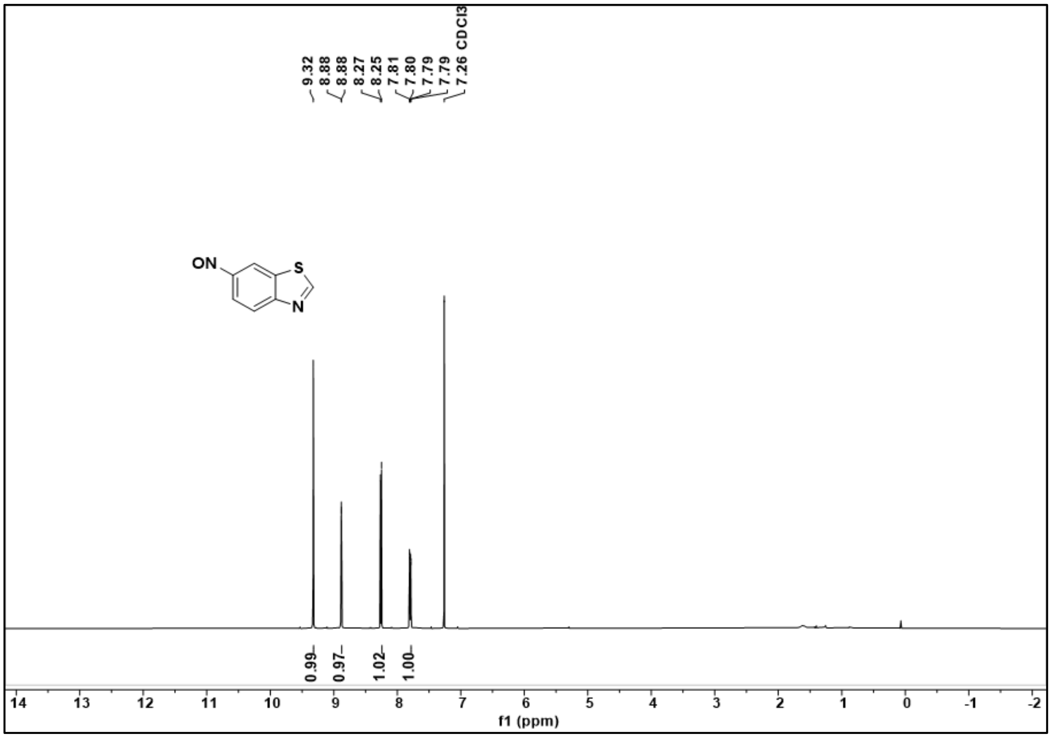

^{1}H NMR spectrum of **16b** ($CDCl_3$, 500 MHz)

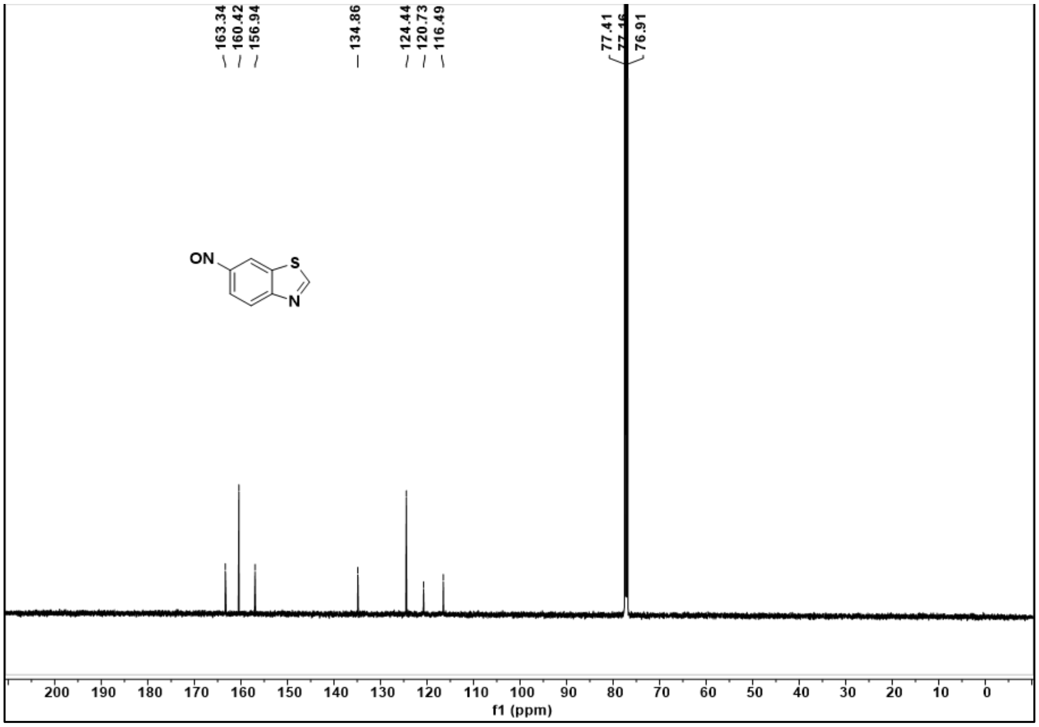

^{13}C NMR spectrum of **16b** ($CDCl_3$, 125 MHz)

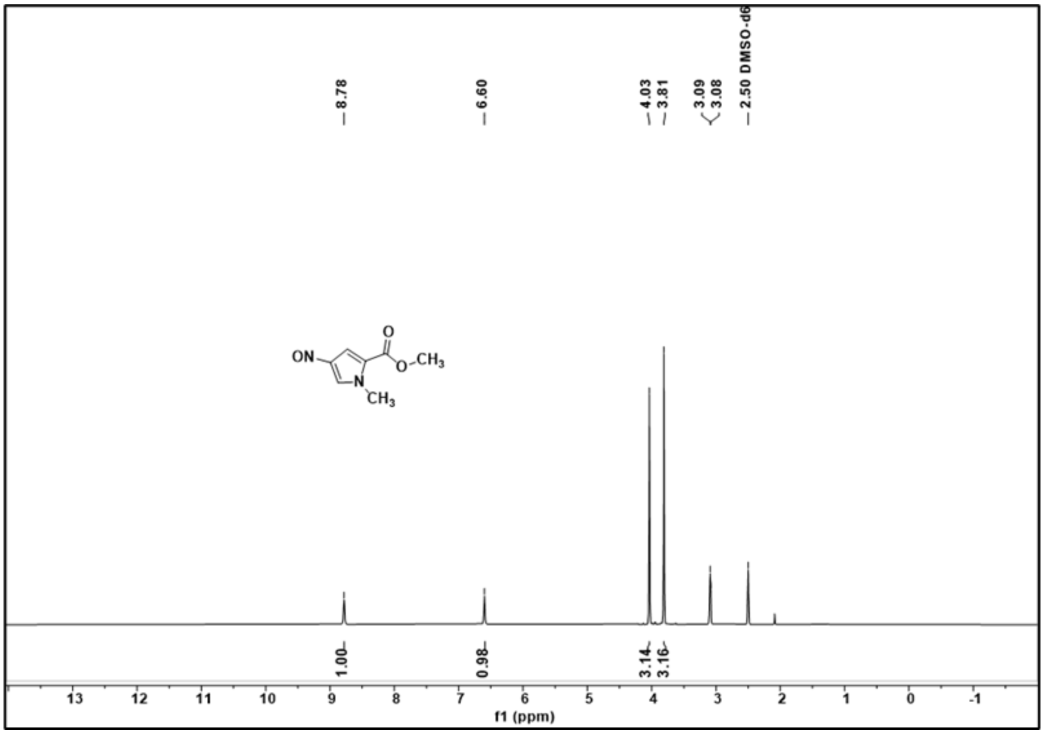

^{1}H NMR spectrum of **17b** (DMSO-d_6, 400 MHz)

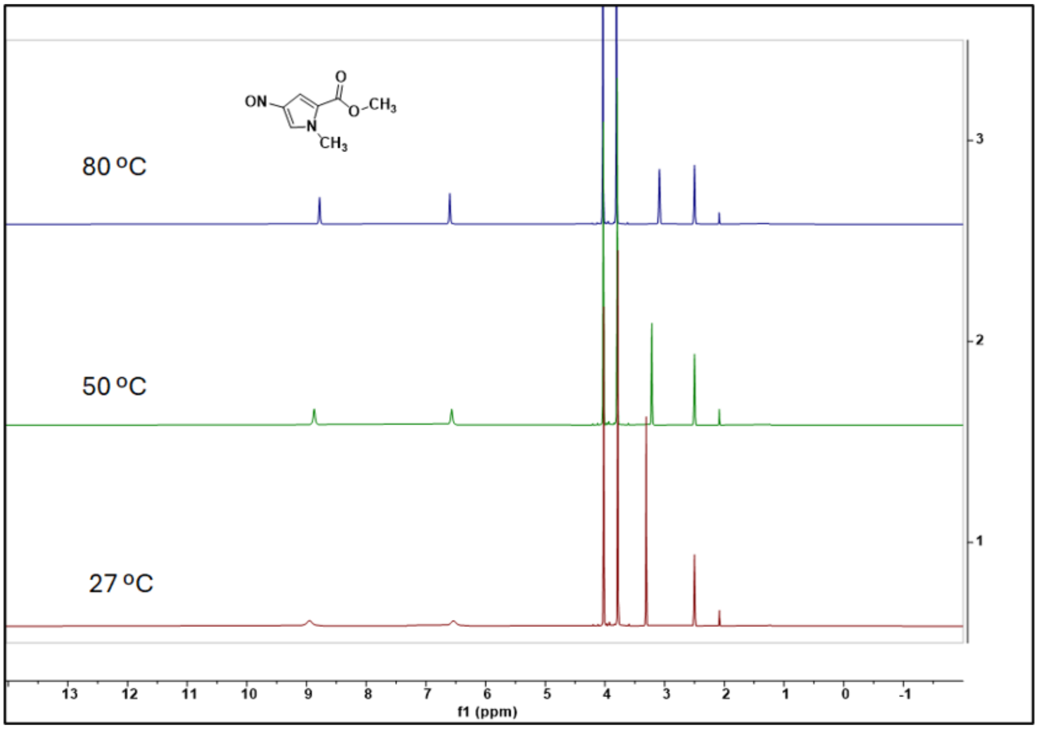

Variable-temperature ^{1}H NMR spectrum of **17b** (DMSO-d_6, 400 MHz)

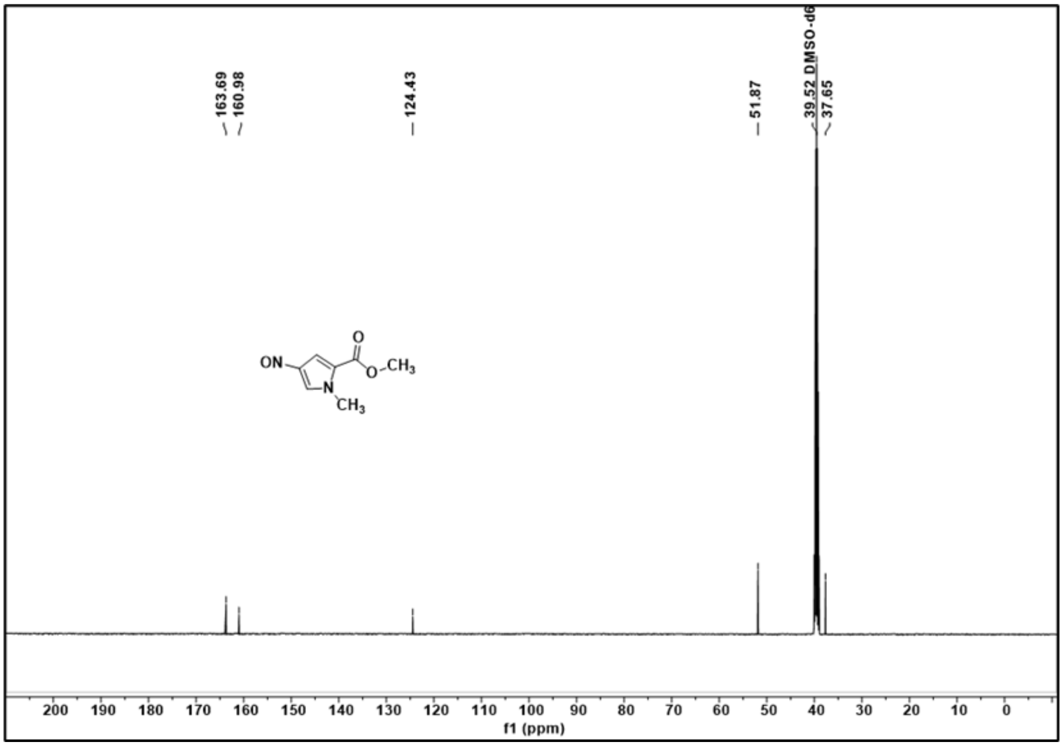

^{13}C NMR spectrum of **17b** (DMSO-d_6, 125 MHz)

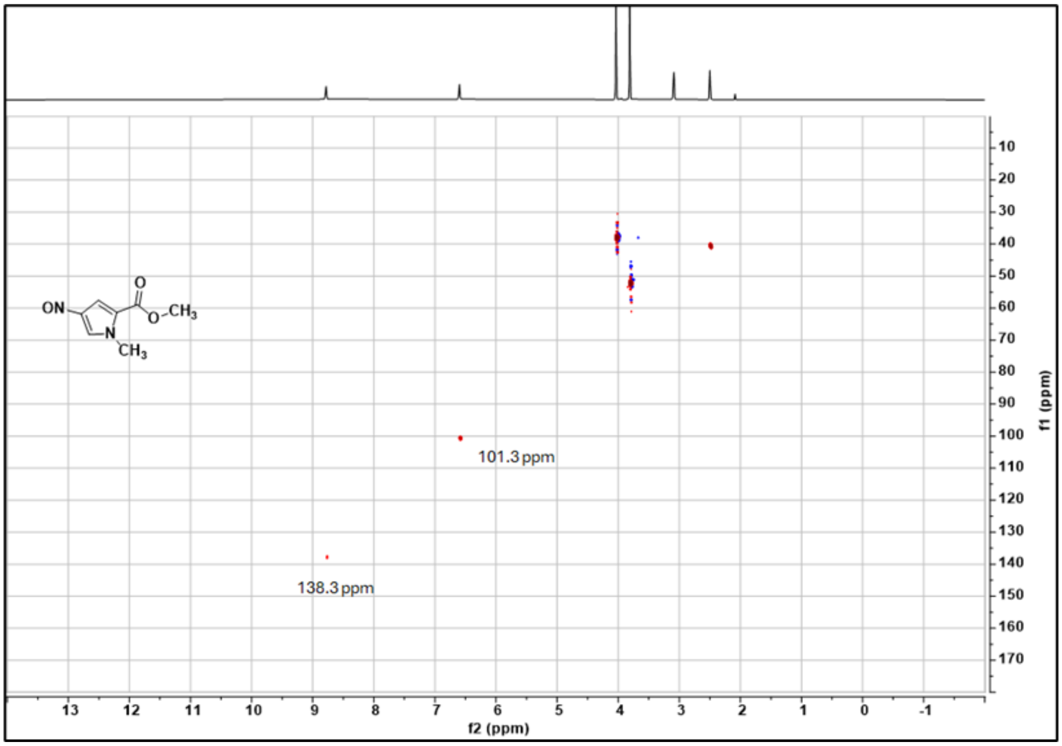

HSQC NMR spectrum of 1**7b** (DMSO-d$_6$, 400 MHz)

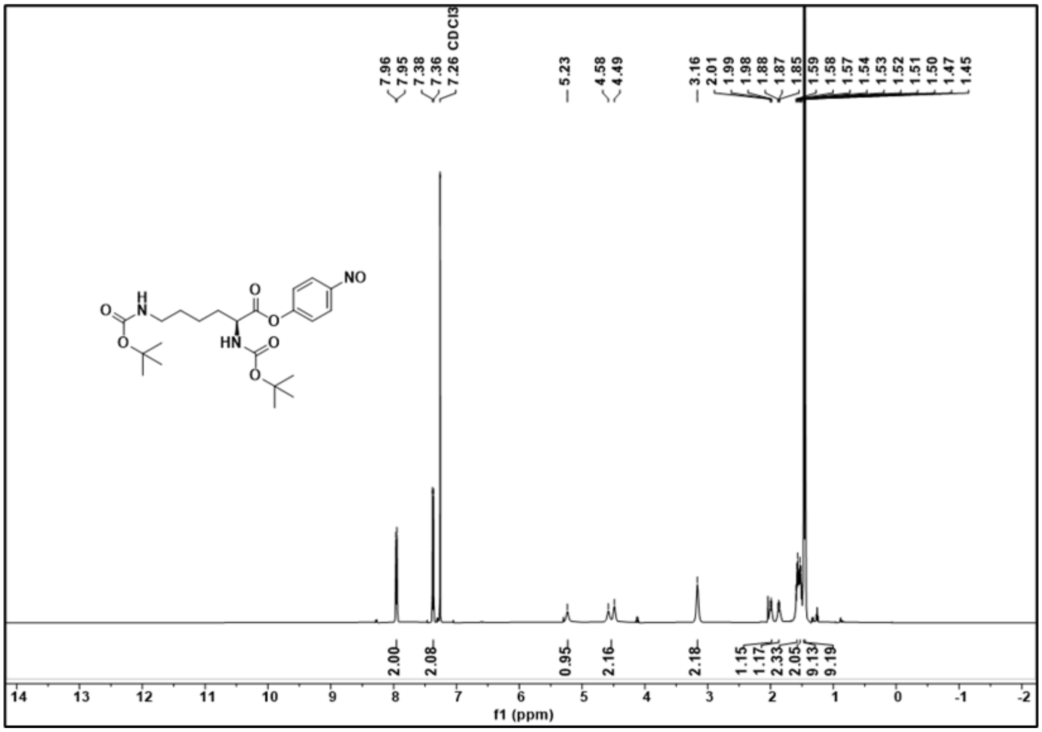

^{1}H NMR spectrum of **18b** (CDCl$_3$, 500 MHz)

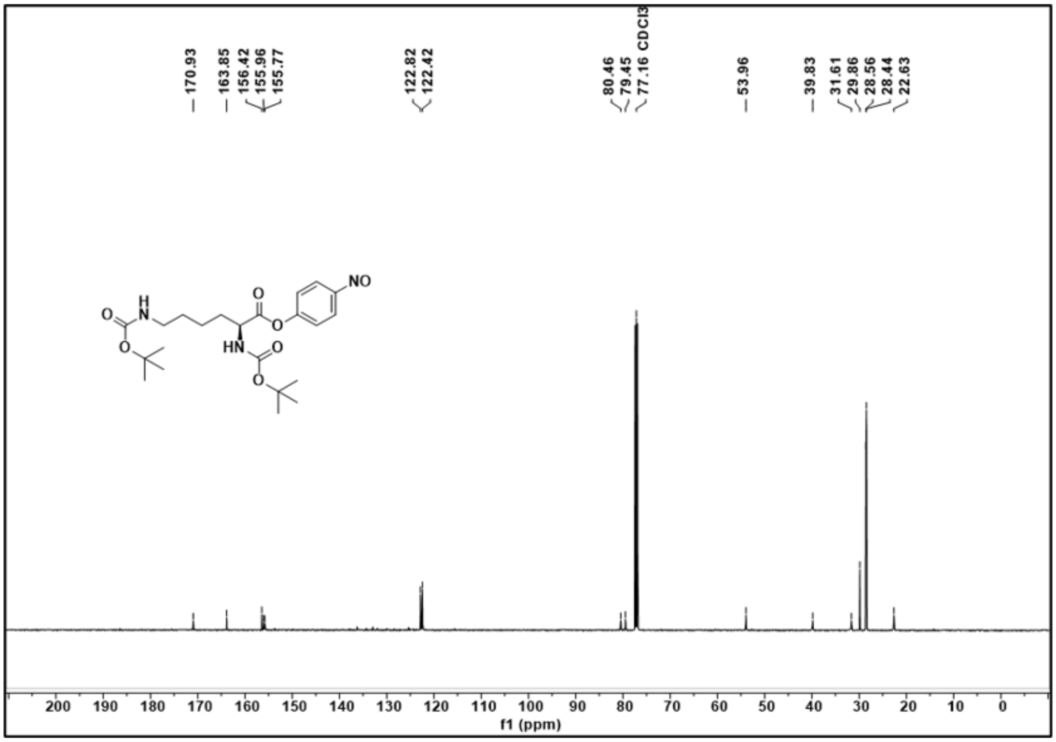

^{13}C NMR spectrum of **18b** ($CDCl_3$, 125 MHz)

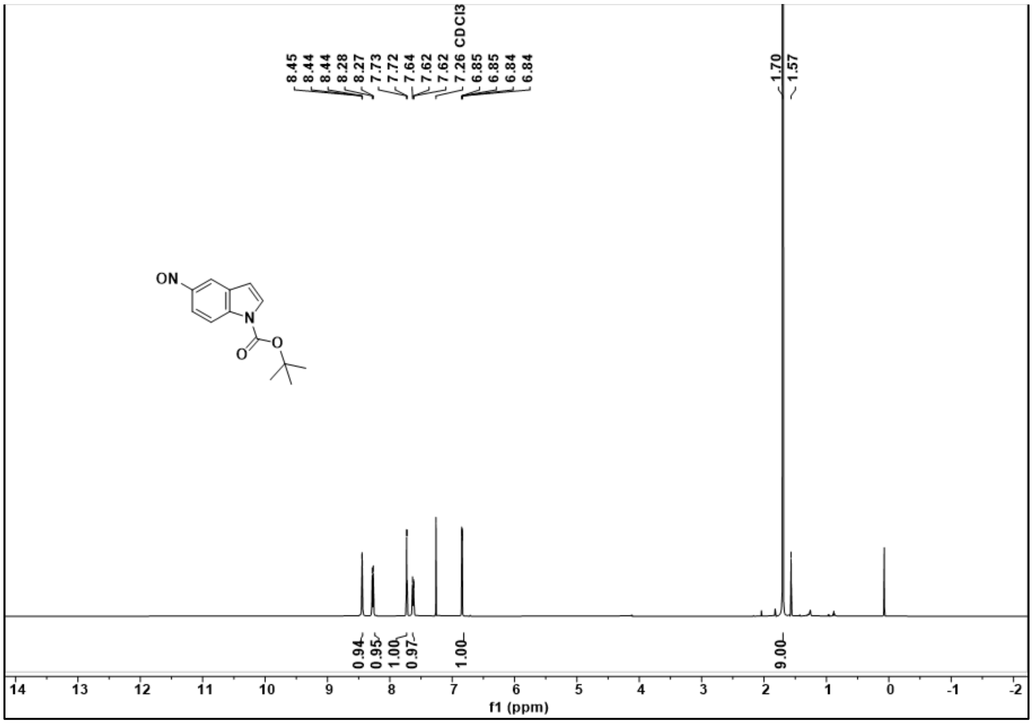

^{1}H NMR spectrum of **19b** ($CDCl_3$, 500 MHz)

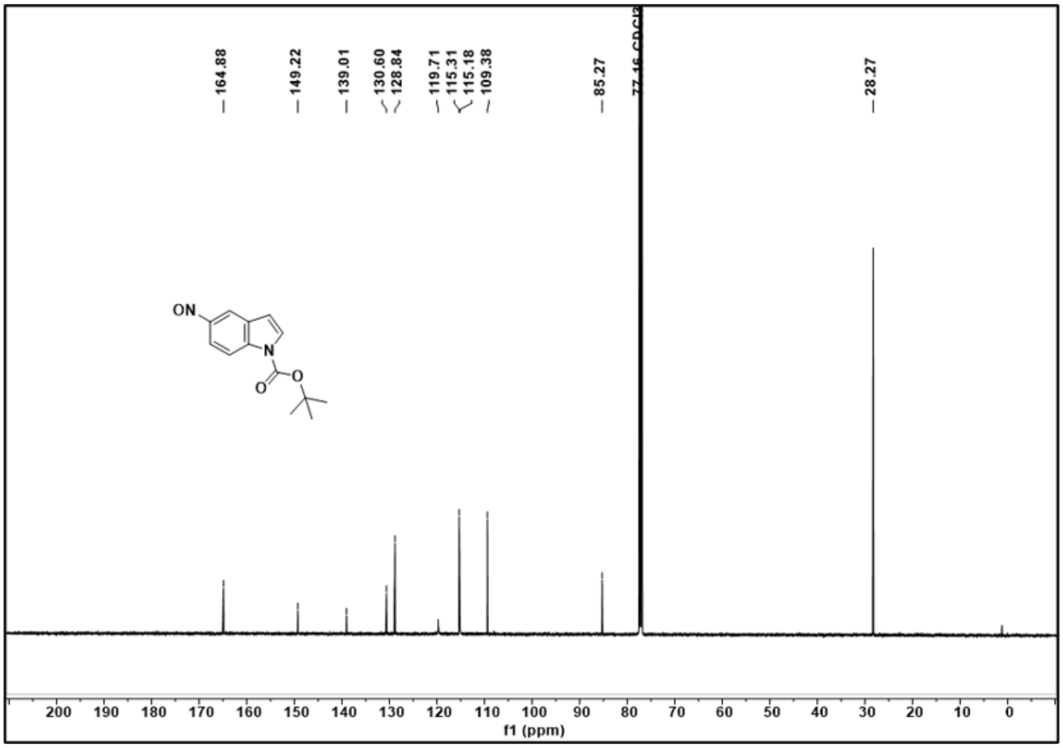

^{13}C NMR spectrum of **19b** ($CDCl_3$, 125 MHz)

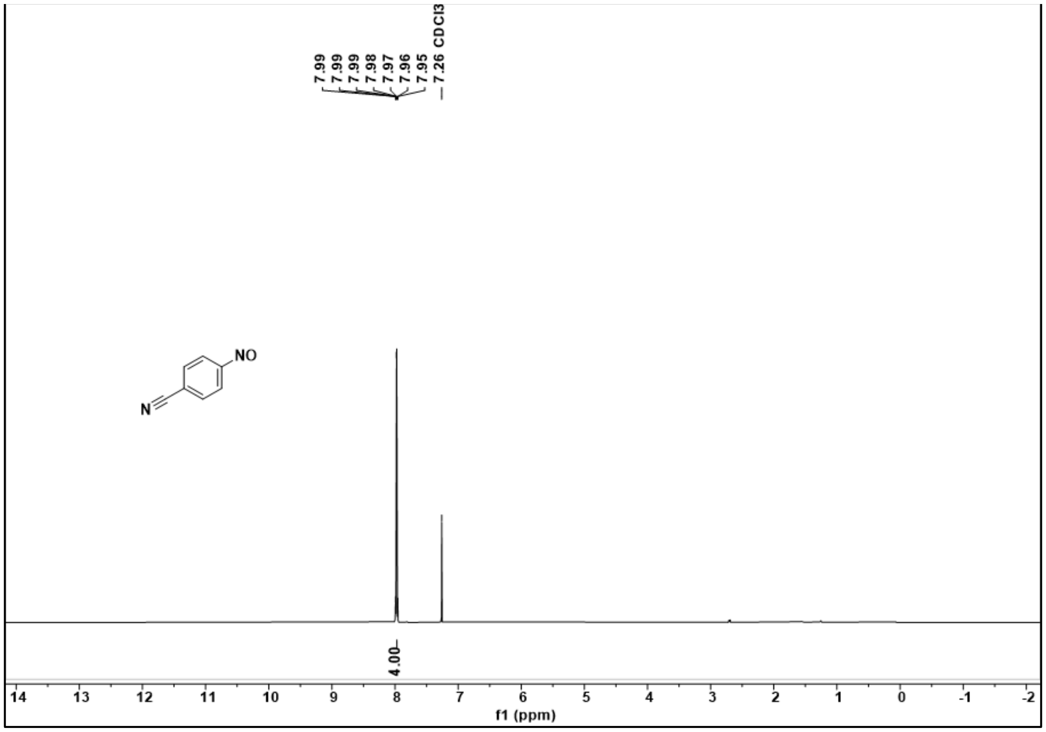

^{1}H NMR spectrum of **20b** ($CDCl_3$, 500 MHz)

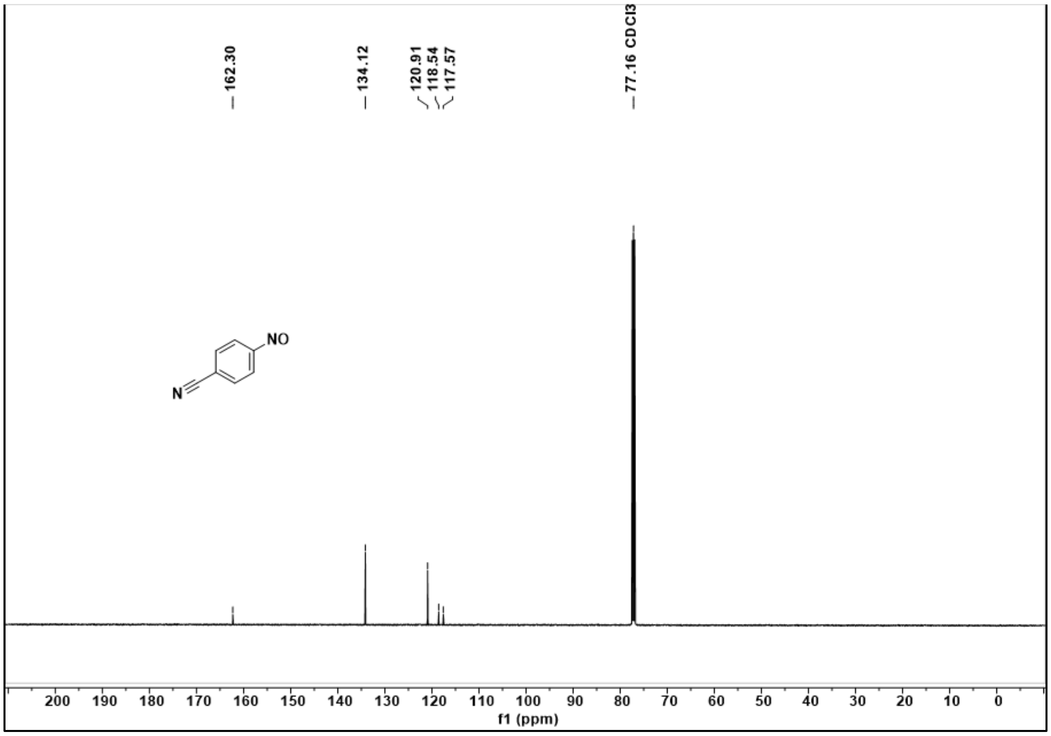

^{13}C NMR spectrum of **20b** ($CDCl_3$, 125 MHz)

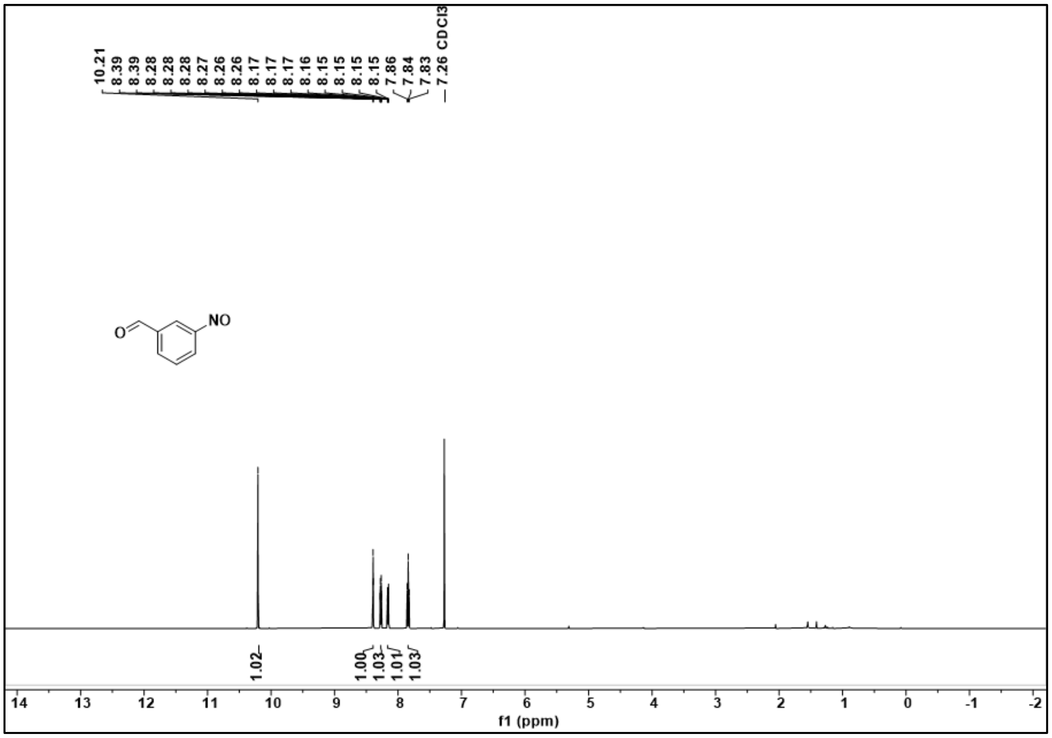

^{1}H NMR spectrum of **21b** ($CDCl_3$, 500 MHz)

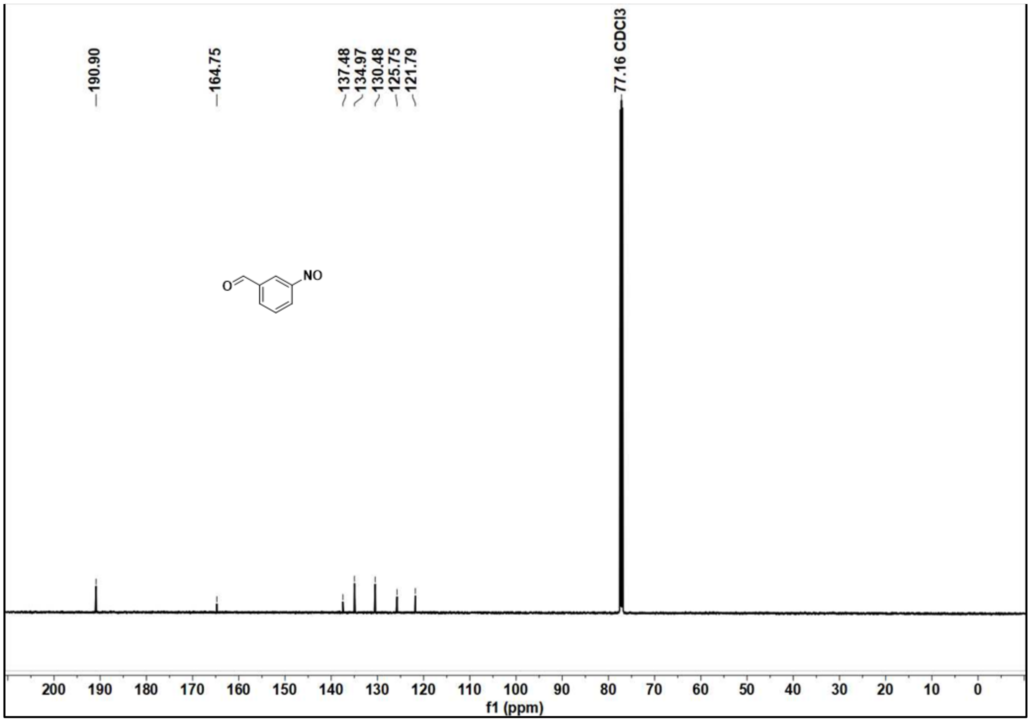

^{13}C NMR spectrum of **21b** ($CDCl_3$, 125 MHz)

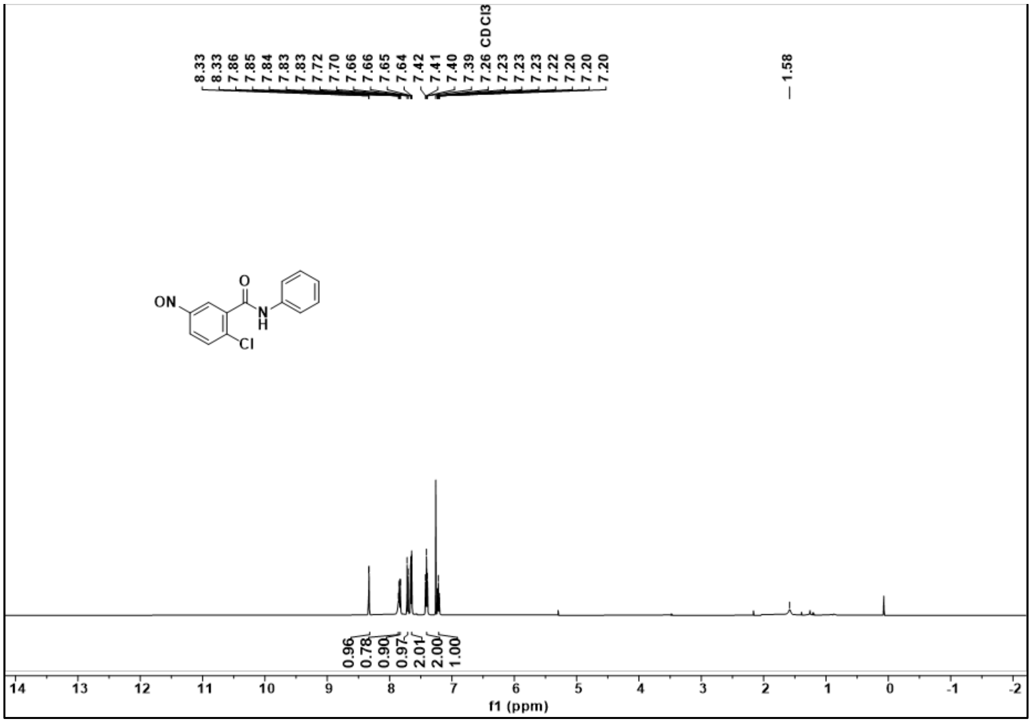

^{1}H NMR spectrum of **22b** ($CDCl_3$, 500 MHz)

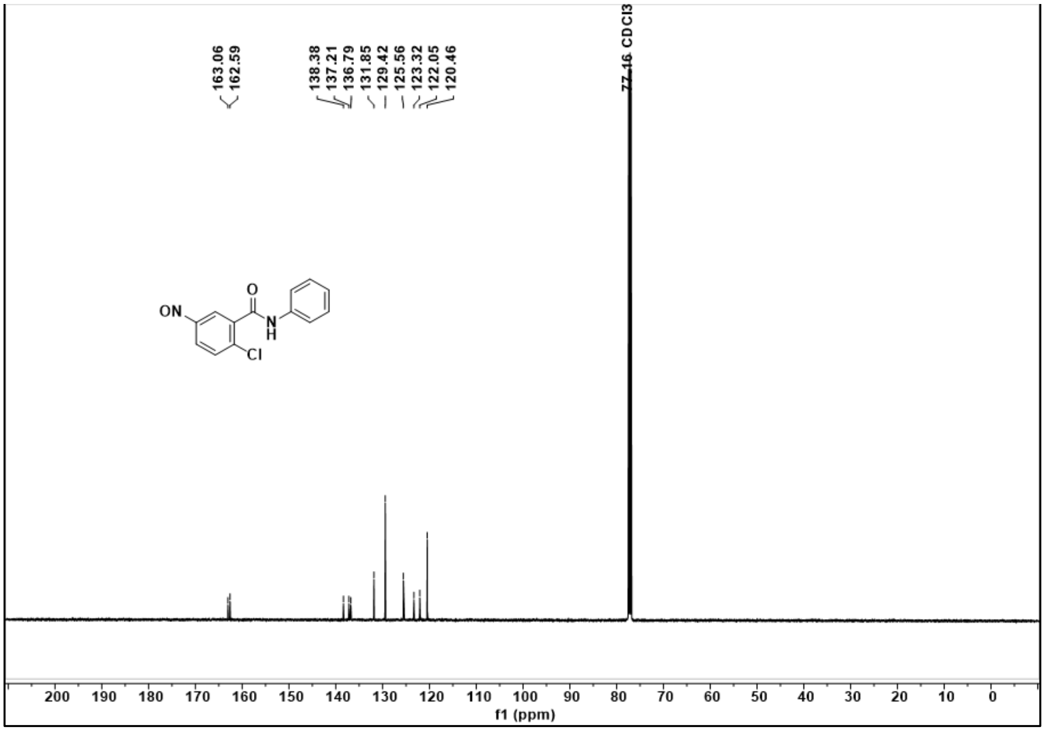

^{13}C NMR spectrum of **22b** ($CDCl_3$, 125 MHz)

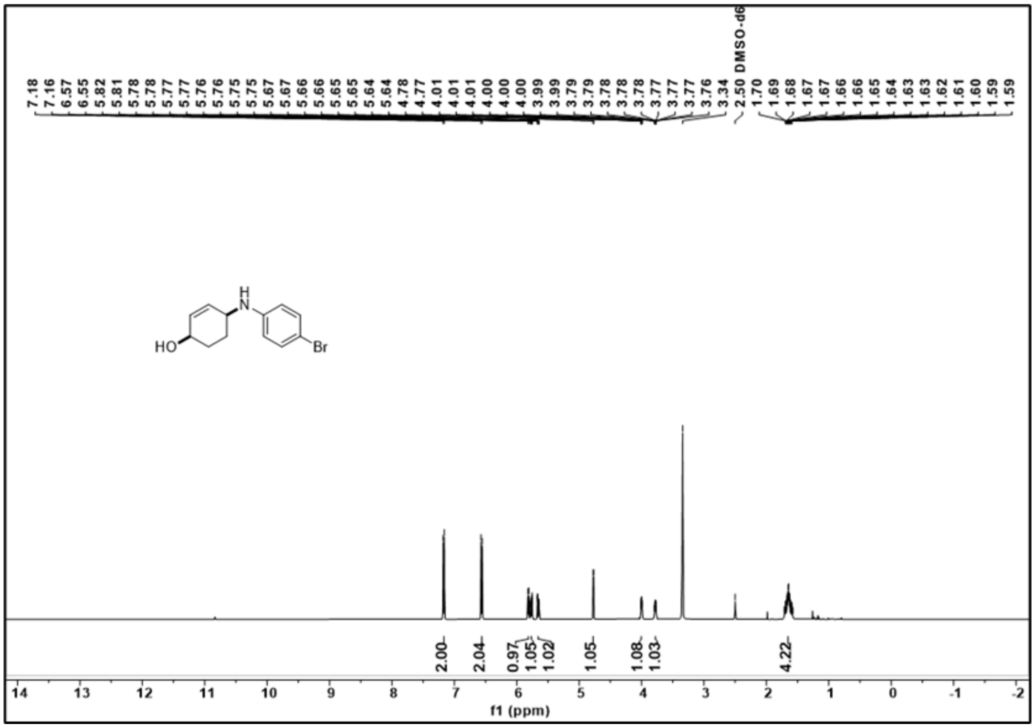

^{1}H NMR spectrum of **23** (DMSO-d_6, 500 MHz)

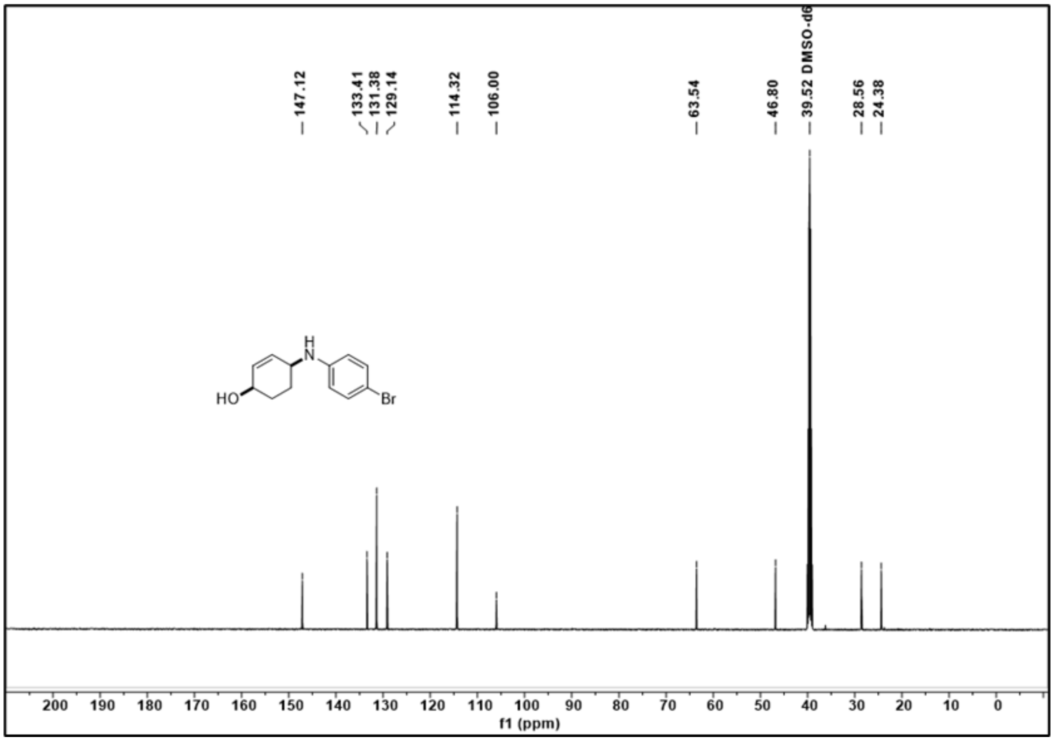

^{13}C NMR spectrum of **23** (DMSO-d$_6$, 125 MHz)

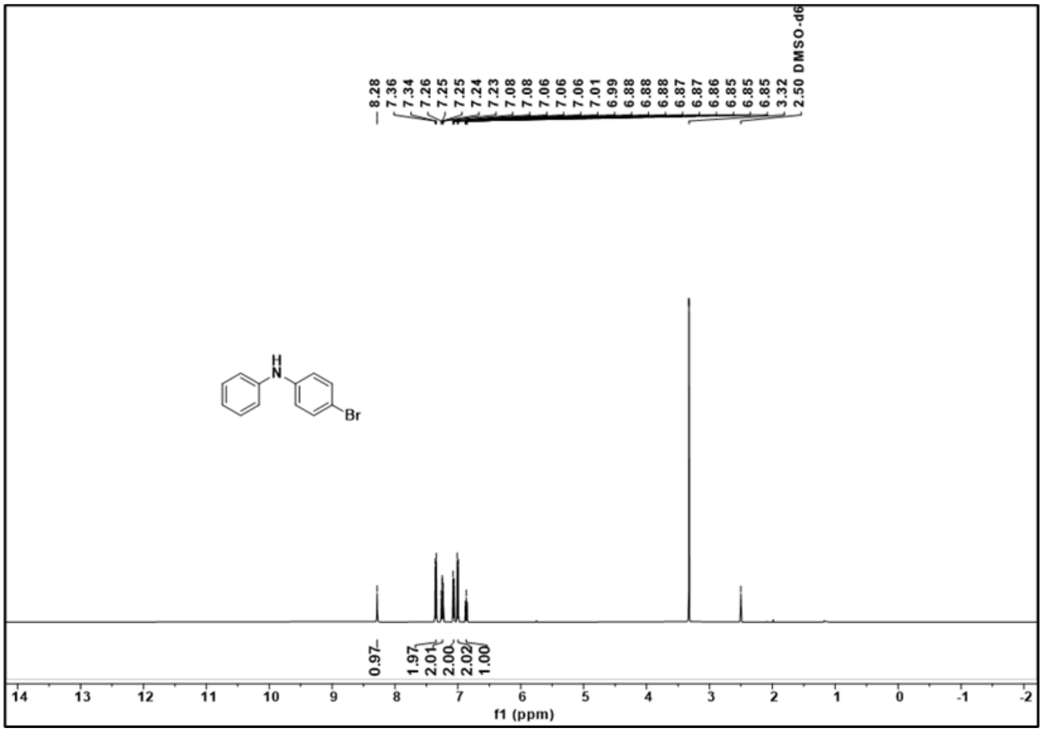

^{1}H NMR spectrum of **24** (DMSO-d$_6$, 500 MHz)

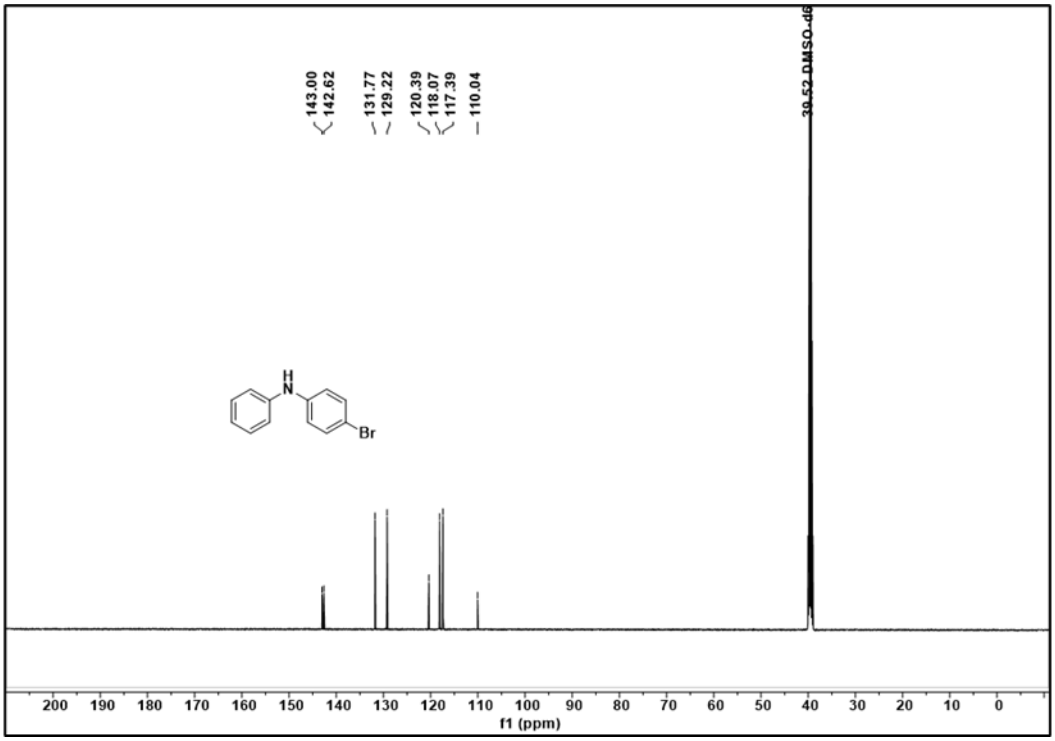

^{13}C NMR spectrum of **24** (DMSO-d$_6$, 125 MHz)

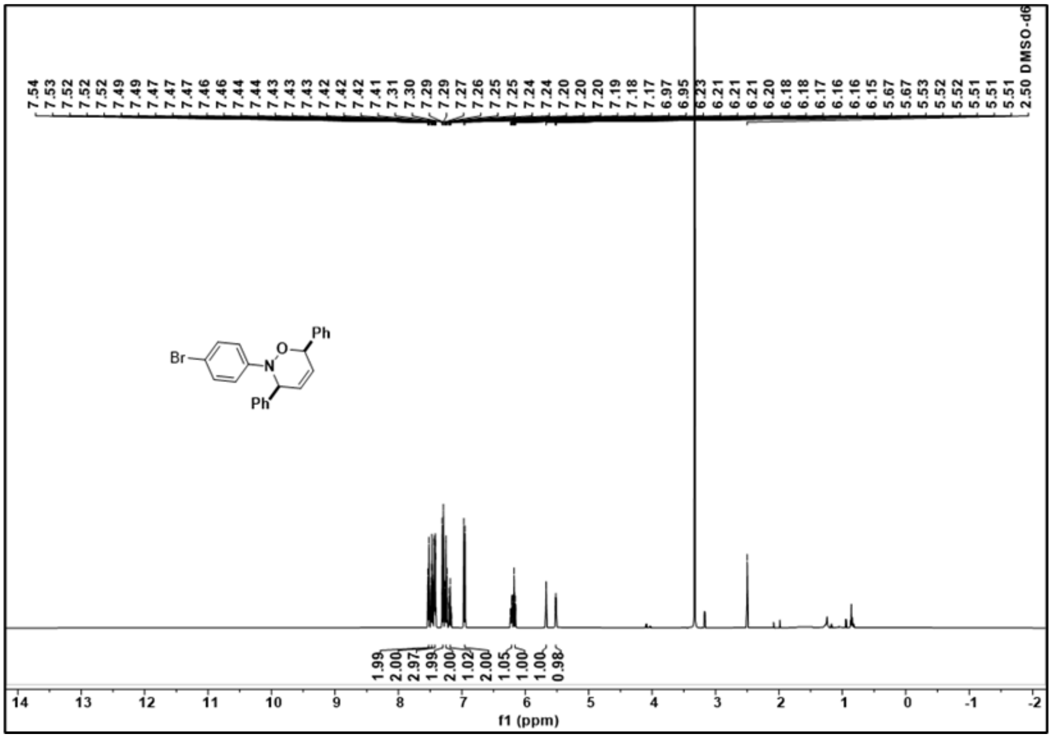

^{1}H NMR spectrum of **25** (DMSO-d$_6$, 500 MHz)

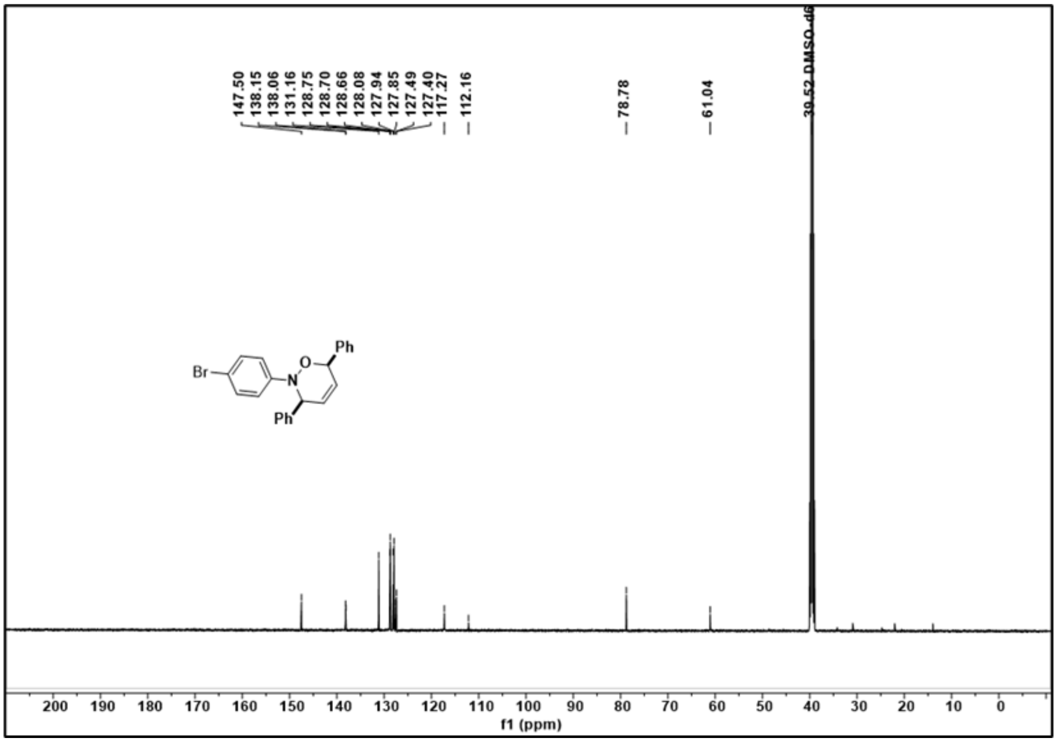

^{13}C NMR spectrum of **25** (DMSO-d$_6$, 125 MHz)

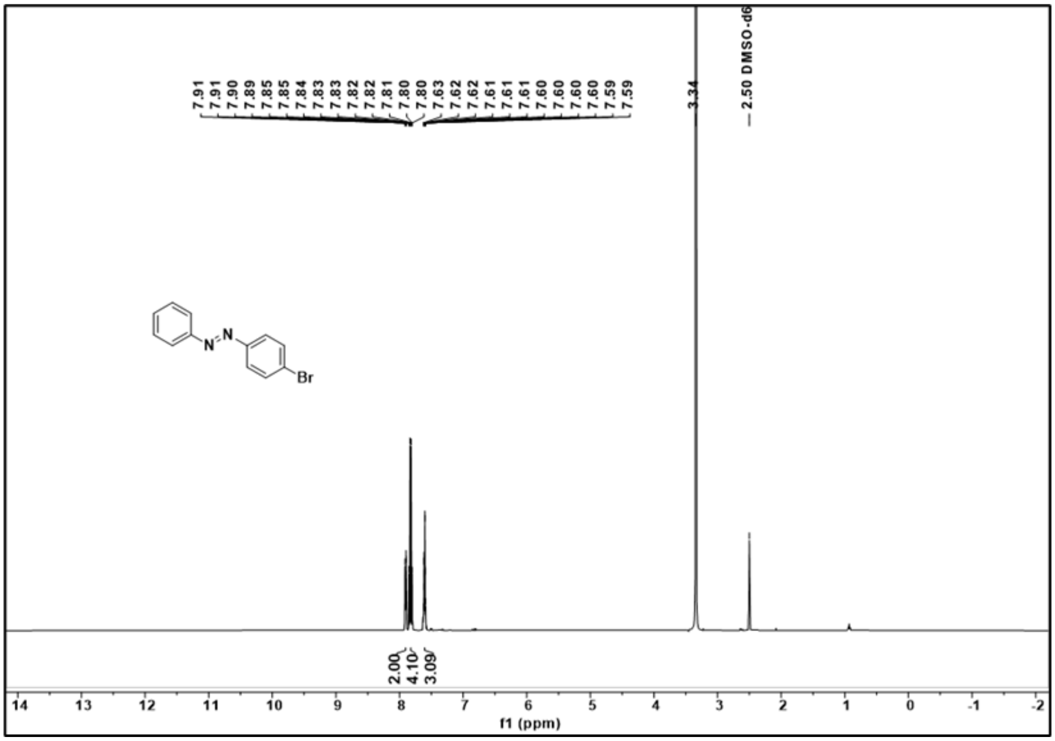

^{1}H NMR spectrum of **26** (DMSO-d$_6$, 500 MHz)

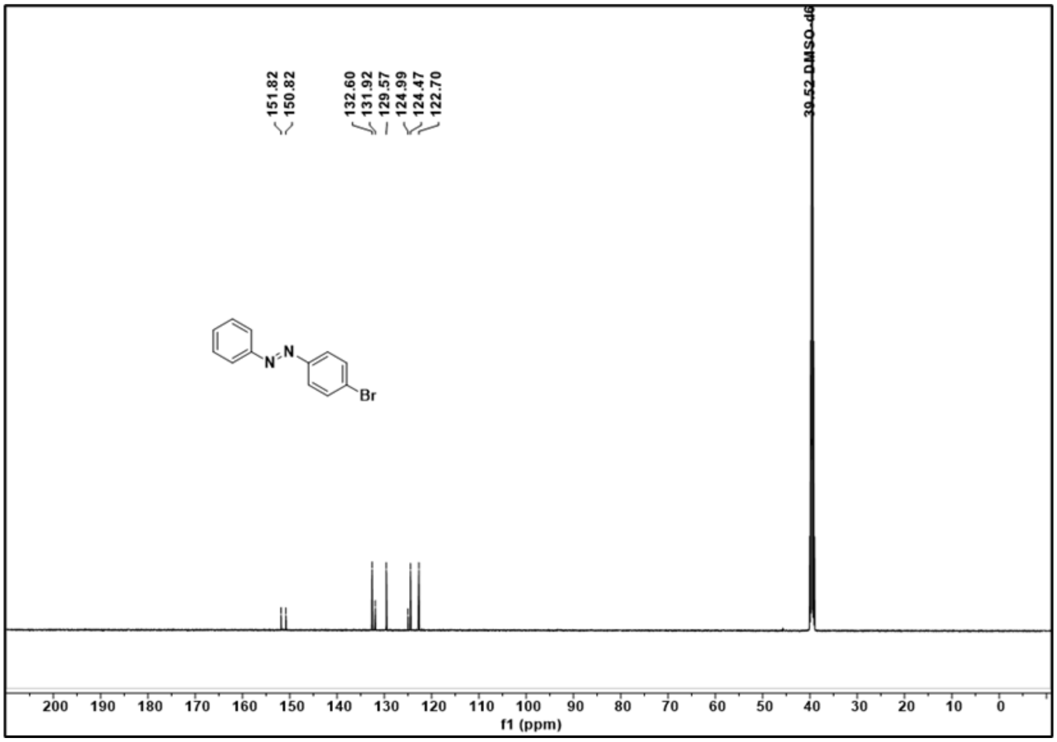

^{13}C NMR spectrum of **26** (DMSO-d$_6$, 125 MHz)

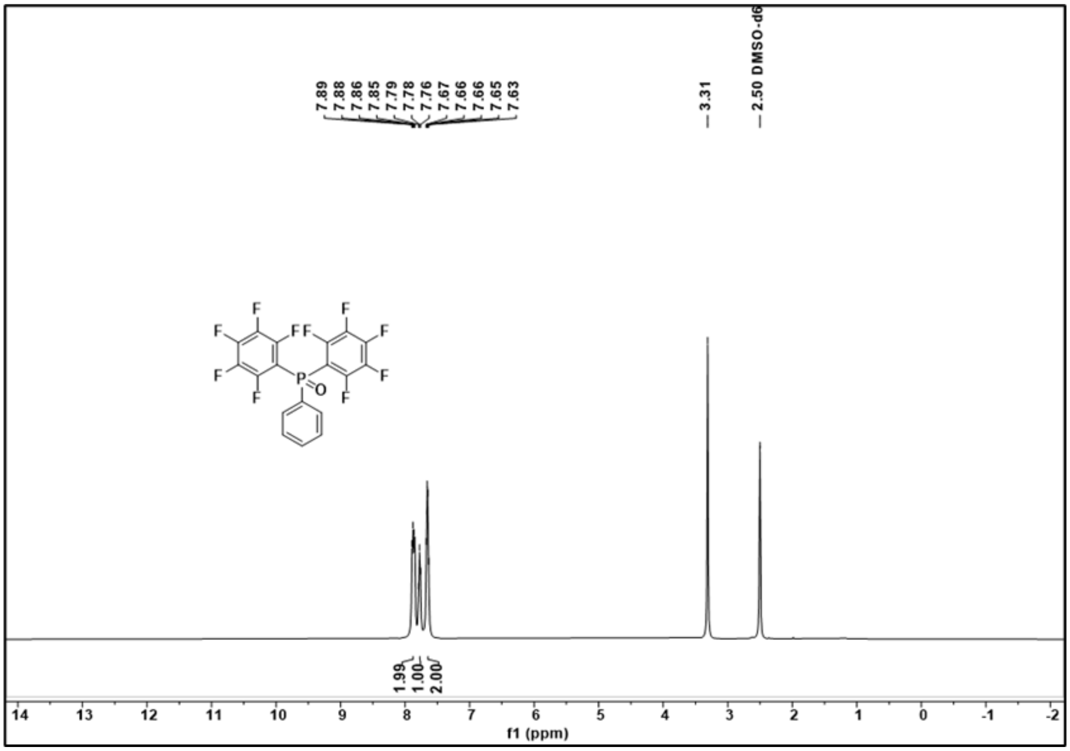

^{1}H NMR spectrum of **S1** (DMSO-d$_6$, 500 MHz)

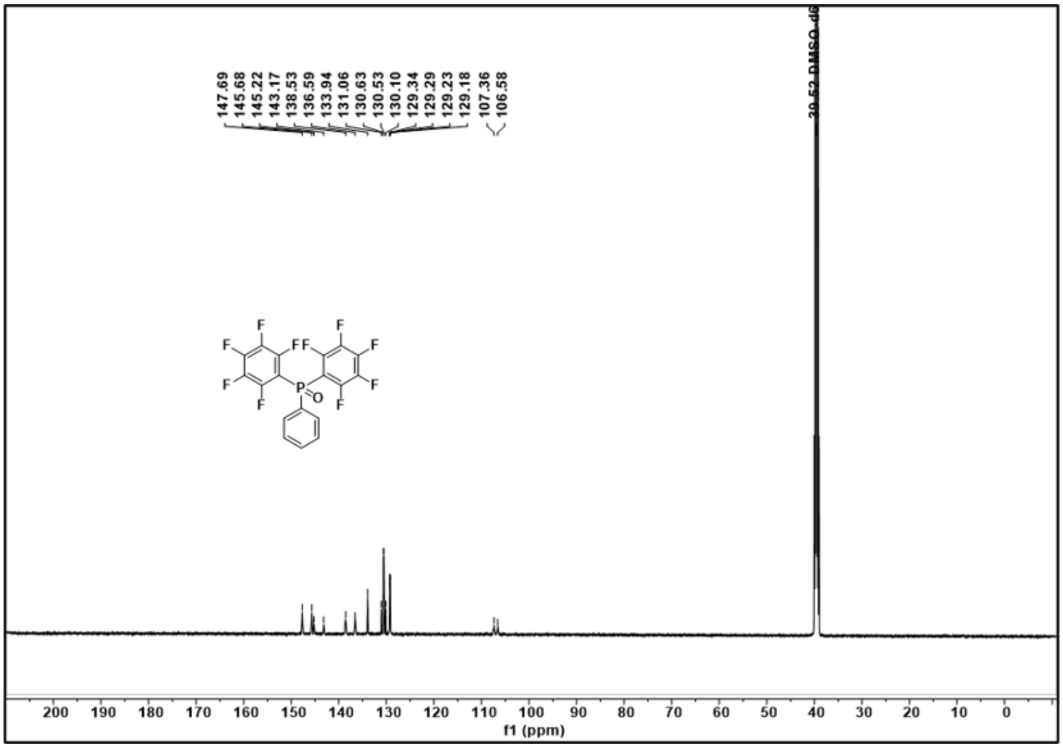

^{13}C NMR spectrum of **S1** (DMSO-d_6, 125 MHz)

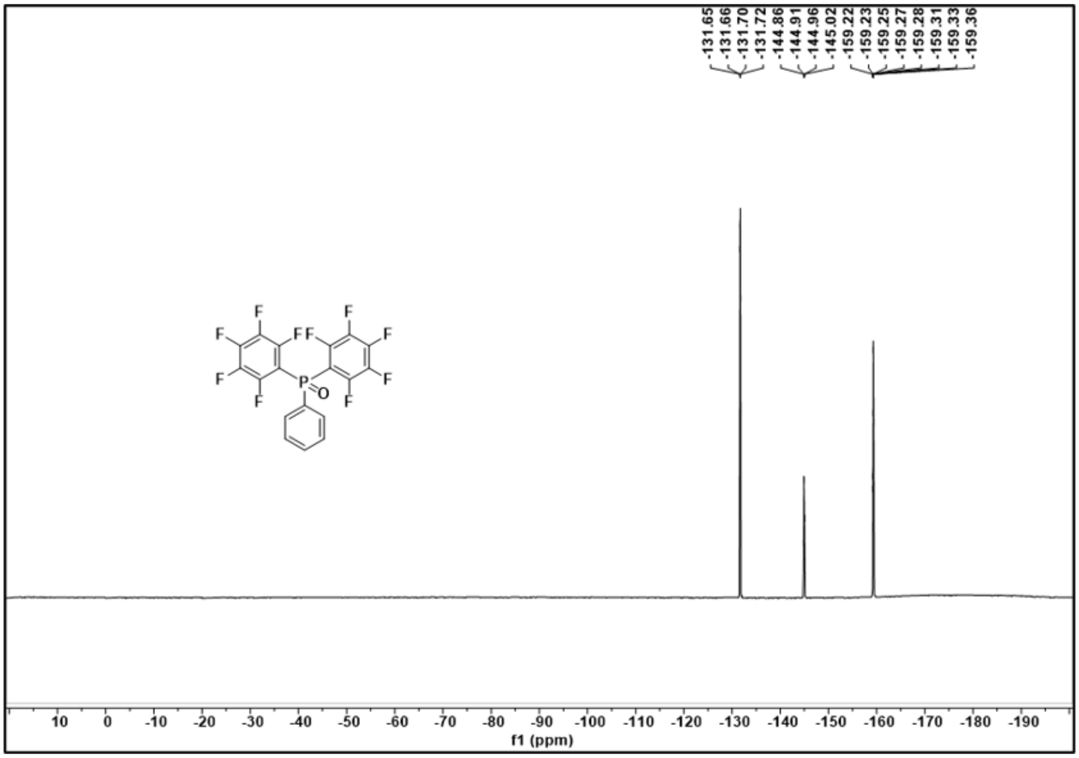

^{19}F NMR spectrum of **S1** (DMSO-d_6, 470 MHz)

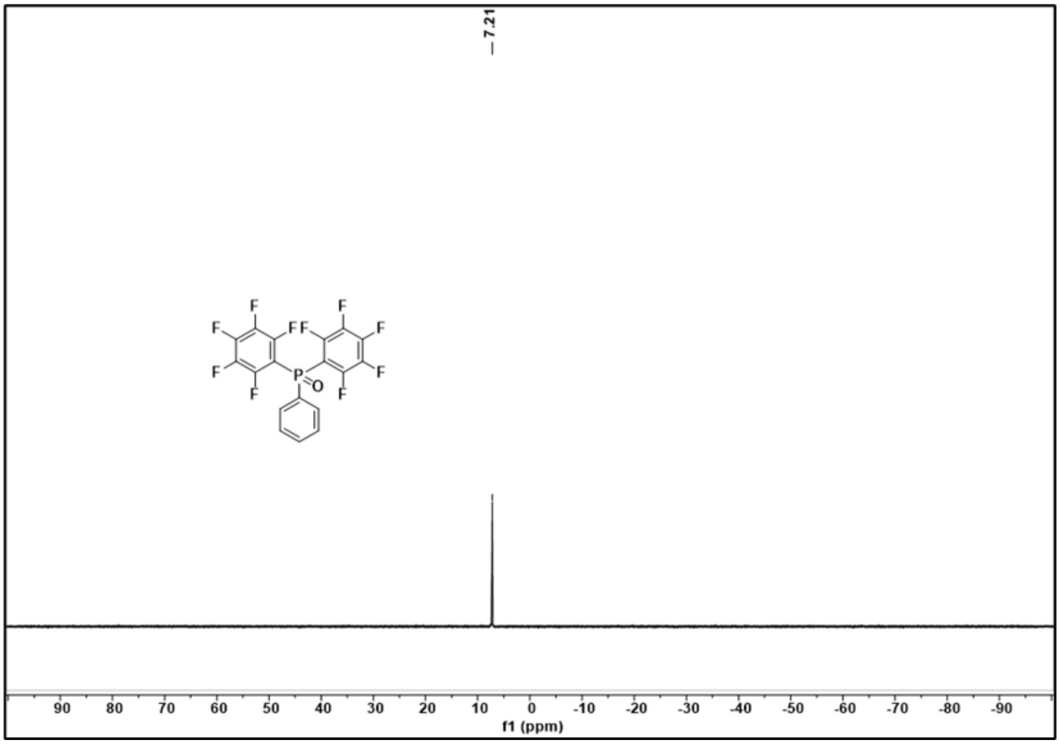

^{31}P NMR spectrum of **S1** (DMSO-d_6, 202 MHz)
